# Early secretory antigen target of 6-kDa of *Mycobacterium tuberculosis* inhibits macrophage apoptosis and host defense via TLR2

**DOI:** 10.1186/s12931-025-03210-z

**Published:** 2025-04-09

**Authors:** Lin Zhang, Fang Fang, Danrui Liu, Geman Xia, Tong Feng, Jingzhu Lv, Jinying Qi, Tengteng Li, Hui Liu, Tao Xu, Fengjiao Wu, Chuanwang Song, Wei Li, Xiaojing Wang, Xianyou Chang, Hongtao Wang, Ting Wang, Zhongqing Qian

**Affiliations:** 1 Anhui Provincial Key Laboratory of Immunology in Chronic Diseases, Anhui Provincial Key Laboratory of Infection and Immunology, and Department of Laboratory Medicine, Bengbu Medical University, Bengbu, Anhui China; 2 Anhui Clinical and Preclinical Key Laboratory of Respiratory Disease, Department of Respiration, First Affiliated Hospital, Bengbu Medical University, Bengbu, Anhui China; 3 The Infectious Disease Hospital of Bengbu City, Bengbu, Anhui China; 4 Department of Internal Medicine, College of Medicine-Phoenix, University of Arizona, Phoenix, AZ USA; 5 Center for Translational Science, Florida International University, Port Saint Lucie, FL USA; 6 Yiwu Traditional Chinese Medicine Hospital, Jinhua, Zhejiang China

**Keywords:** ESAT-6, Apoptosis, TLR2, Macrophage, *M. tb*

## Abstract

*Mycobacterium tuberculosis* (*M. tb*) is an intracellular pathogen adept at evading the human immune system through a variety of mechanisms. During infection, *M. tb* secretes numerous virulence factors, including the 6 kDa early secretory antigen target (ESAT-6), which is produced by the ESX-1 secretion system. ESAT-6 plays a crucial role in host–pathogen interactions, either independently or in association with culture filtrate protein 10 (CFP-10). While some research has investigated the role of ESAT-6 in *M. tb* pathogenicity and vaccine development, its precise contribution to immune evasion and the cellular mechanisms involved remain poorly understood. To address this, we used cultured THP-1(A) macrophages to characterize the effects of secreted ESAT-6 on cellular host defenses and apoptosis. We found that ESAT-6 (5 μg/ml) inhibited *M. tb*-induced apoptosis in THP-1(A) macrophages by suppressing Toll-like receptor 2 (TLR2) through the Caspase-9/Caspase-3 pathway. Additionally, ESAT-6 reduced phagocytosis of *M. tb* by THP-1(A) macrophages by downregulating the production of interleukin-10 (IL-10), tumor necrosis factor-alpha (TNF-α), and interleukin-12 (IL-12). Furthermore, ESAT-6 diminished the bactericidal activity of macrophages by inducing reactive oxygen species (ROS) production. In parallel, our in silico analysis of differentially expressed genes in dendritic cells (DCs) infected with Bacille Calmette-Guérin (BCG) strains, with or without the region of difference-1 (RD1) gene, strongly suggests that ESAT-6, located within the RD1 region, modulates host defense functions and apoptosis in DCs during BCG infection. Collectively, these findings indicate that ESAT-6 plays a pivotal role in modulating the innate immune response of macrophages against *M. tb* by regulating macrophage recognition, phagocytosis, bactericidal activity, and apoptosis. Our study provides valuable insights into potential molecular targets for the development of innovative vaccines and therapeutic strategies against *M. tb*.

**Supplementary Information:**

The online version contains supplementary material available at 10.1186/s12931-025-03210-z.

## Introduction

Tuberculosis (TB) remains a significant global health challenge, contributing substantially to illness and mortality worldwide [1]. In 2007, TB overtook acquired immunodeficiency syndrome (AIDS) as the leading cause of death from infectious diseases. Current statistics indicate that this preventable and curable disease still afflicts more than 10 million people every year. The COVID-19 pandemic has further exacerbated the situation, reversing years of progress in the fight against TB. In 2022, 7.5 million people were newly diagnosed with TB, which causes almost twice as many deaths as HIV/AIDS [1, 2]. Presently, the primary strategies for combating TB are limited to antibiotic treatments, while there has been no breakthrough in the development of new TB vaccines for many years. The lack of effective immune-based protection underscores the urgent need for further research into the virulence mechanisms *of M. tb*, the pathophysiology of the disease, and the creation of innovative vaccines to combat TB [3].

**Fig. 1:**
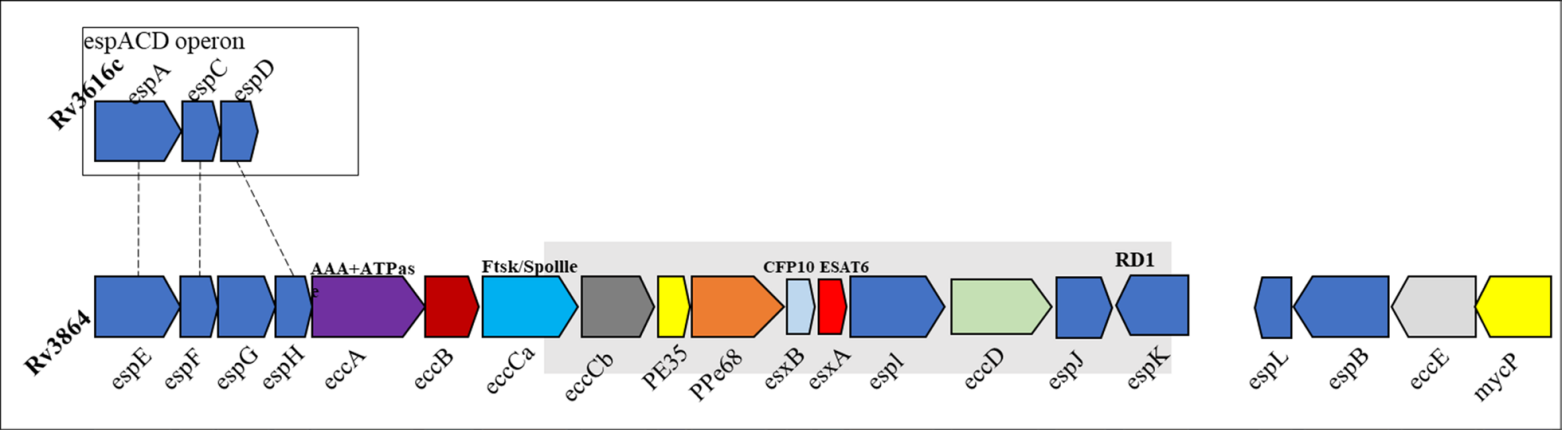
Genomic location of ESAT6 in the RD1 region of *M. tb* (Rv3616c and Rv3684). The grey shaded part is RD1 region

The 6-kDa early secretory antigen target (ESAT-6) is a virulent protein secreted by the unique type VII secretion system ESX-1 within the region of difference-1 (RD1) gene cluster of *M. tb* [4]. Notably, ESAT-6, along with its partner protein culture filtrate protein-10 (CFP-10) (Rv3874 and Rv3875), is secreted by the highly virulent H37Rv strain (Fig. 1) but not by the attenuated H37Ra strain. Early studies have highlighted the significant role of ESAT-6 in the pathogenesis of *M. tb* [5]. Interestingly, restoring ESAT-6 secretion in the Bacillus Calmette-Guérin (BCG) vaccine strain enhanced the immunogenicity of BCG but also increased its virulence [6]. These findings led us to hypothesize that ESAT-6 plays a critical role in the evasion of host immunity by *M. tb*.

Toll-like receptors (TLRs) are essential components of the innate immune system, playing a critical role in the inflammatory response to invading pathogens. In the context of *M. tb* infection, numerous studies have demonstrated that Toll-like receptor 2 (TLR2) [7–9] and Toll-like receptor 4 (TLR4) [8–10] are crucial for the recognition of *M. tb* and the initiation of the inflammatory defense response [11, 12]. As frontline defenders, macrophages can phagocytose *M. tb*, leading to the inhibition or killing of the pathogen, a process that is at least partially mediated by TLR activation. However, *M. tb* has evolved various strategies to evade the microbicidal responses of macrophages. These strategies include inhibiting phagosome maturation [13], modulating the production of pro- and anti-inflammatory cytokines [14, 15], and suppressing apoptosis [16]. Among these, the suppression of apoptosis via TLRs [17] is a key strategy employed by virulent strains of *M. tb*. Unlike necrosis, which can lead to bacterial proliferation, dissemination, local inflammation, and tissue damage [18], apoptosis plays a protective role by promoting antigen presentation and restricting the release of intracellular bacteria [19], thereby enhancing the host’s immune defense.

During *M. tb* infection, pathogen-induced apoptosis in macrophages is crucial for controlling the infection, as it activates both innate and adaptive immune responses through distinct molecular pathways [20, 21]. Virulent strains of *M. tb*, however, suppress apoptosis in host macrophages, allowing the bacteria to survive and replicate within the host cells. Given this context, it is essential to investigate whether the secreted virulent protein ESAT-6 interferes with macrophage apoptosis and to elucidate the precise molecular mechanisms involved.

Bioinformatic and cell biology analysis tools were employed to investigate the involvement of ESAT-6 in the immune evasion mechanisms of *M. tb*. The functional impact of ESAT-6 on host cell defense and apoptosis was characterized using in vitro experiments with THP-1(A) macrophages. Our findings revealed that ESAT-6, at an optimal concentration of 5 μg/ml, inhibited H37Ra-induced apoptosis in THP-1(A) macrophages through the TLR2-mediated Caspase-3/Caspase-9 pathways. Additionally, ESAT-6 modulated the immune defense of macrophages against *M. tb* by inhibiting the production of key cytokines, including interleukin-10 (IL-10), interleukin-12 (IL-12), and tumor necrosis factor-alpha (TNF-α). To validate these results, our in silico analyses confirmed that the RD1 region, which includes the ESAT-6 gene, differentially regulates host genes involved in tuberculosis and apoptosis pathways (Supplementary Figs. S1, S2). This study sought to confirm the role of ESAT-6 in *M. tb* evasion of macrophage-mediated immune responses.

Our research elucidates the molecular mechanisms by which *M. tb* evades host immune responses, particularly in the context of macrophage-mediated innate immunity. These insights could enhance the theoretical understanding of tuberculosis pathogenesis and contribute to the development of innovative therapeutic interventions and more effective vaccines for TB patients.

## Results

### ESAT-6 inhibits apoptosis of THP-1(A) macrophages via TLR2

**Fig. 2:**
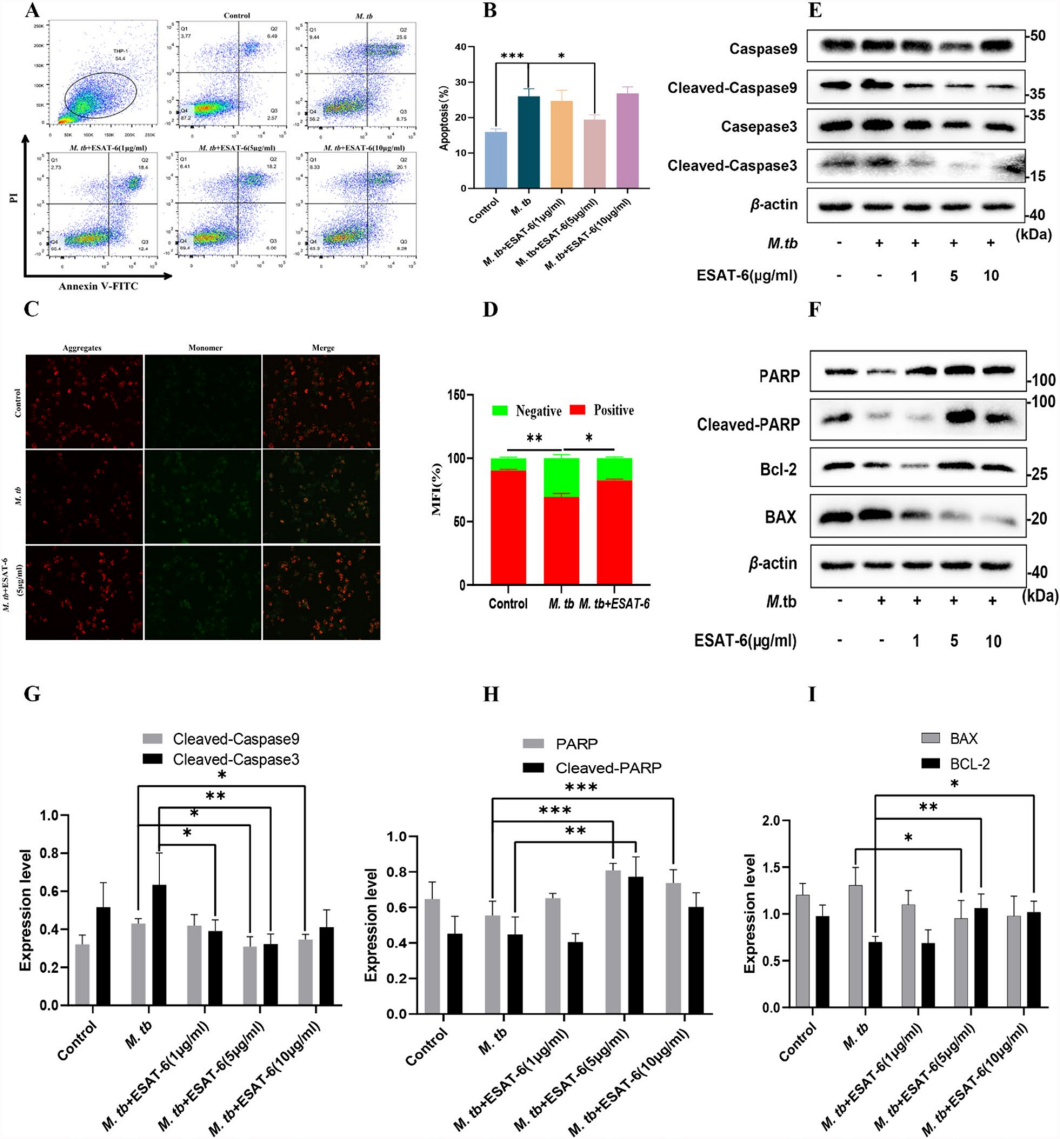
Recombinant ESAT-6 protein inhibits *M. tb*-induced apoptosis of THP-1(A) via Caspase 9/Caspase 3. **A** The effects of *M. tb* and ESAT-6 on macrophages. Cellular apoptosis was evaluated by flow cytometry. THP-1(A) cells (1 × 10^6^) were co-incubated with *M. tb* or/and different concentrations of ESAT-6 for 24 h. The dose-dependent response of EAST-6 on macrophage apoptosis was detected by the Annexin V FITC/PI method. **B** Quantitative data of apoptosis in ESAT-6 and/or mycobacteria stimulated macrophages are shown. **C** THP-1(A) cells (1 × 10^6^) were co-incubated with *M. tb* or/and different concentrations of ESAT-6 for 24 h. Cells received ESAT-6 and *M. tb* were stained with JC-1 for mitochondrial membrane potential measurement. A representative group image of stained cells was observed under an inverted fluorescence microscope. **D** Quantitative data of MFI in ESAT-6 and/or mycobacteria stimulated macrophages are shown. After different concentrations of ESAT-6 were simultaneously added with *M. tb* on THP-1 (A) cells for 24 h, the cells were analyzed by Western blotting for **E** Casepase 3, cleaved-Caspase 3, Caspase 9, and cleaved-Caspase 9 protein levels, **F** PARP and it’s activated form, **F** BAX, and Bcl-2 were analyzed by Western blotting in THP-1(A) cells challenged with *M. tb*. (G-I) Densitometric quantification of representative blots relative to *β*-actin levels. Data are representative of at least three different experiments. Results are mean ± SEM. *p < 0.05,**p < 0.01 and ***p < 0.001, respectively, compared with the *M. tb* group

**Fig. 3:**
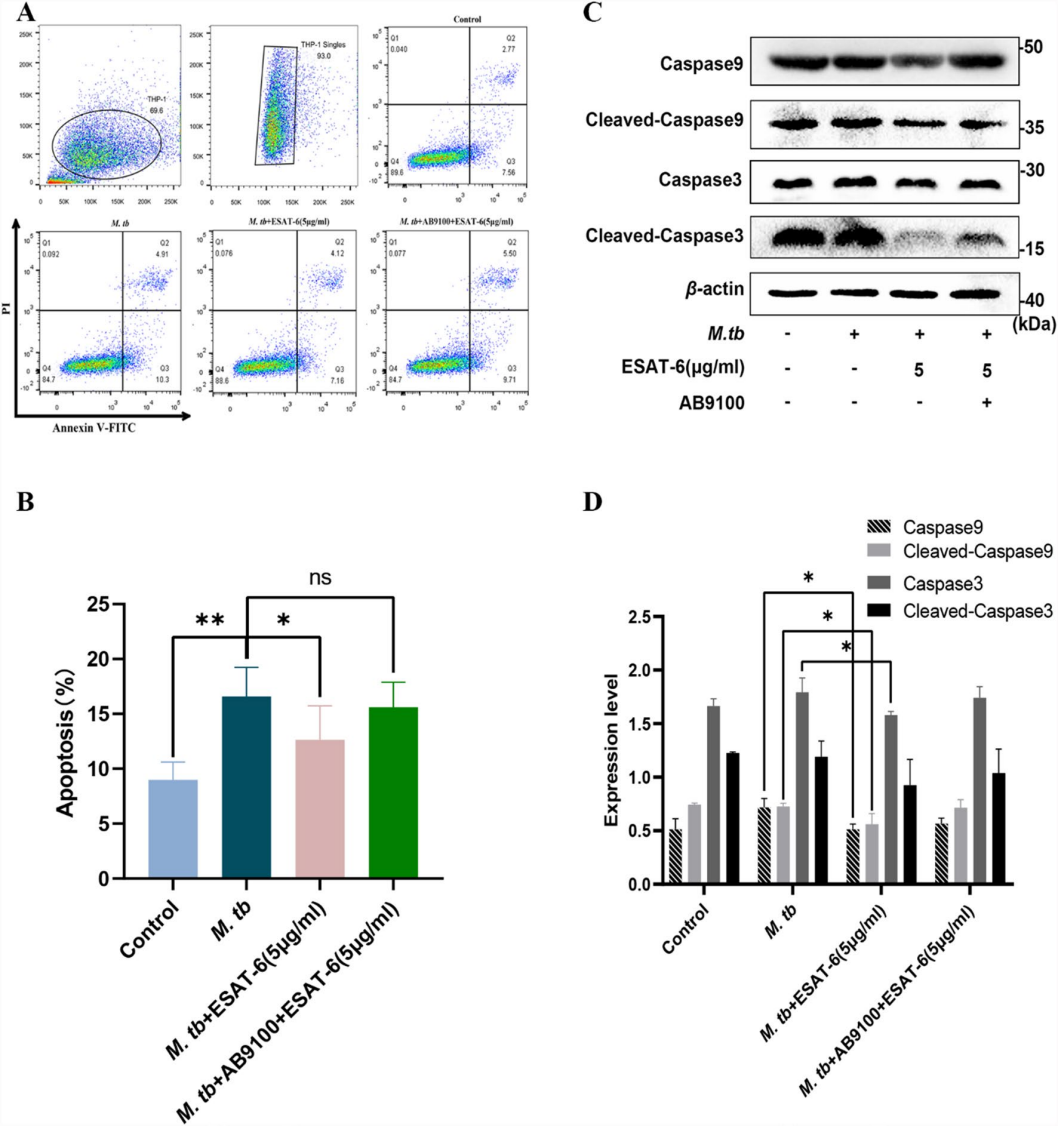
ESAT-6 inhibits apoptosis of THP-1（A）macrophages via TLR2-Caspase 9/Caspase 3. **A** ESAT-6 inhibits apoptosis of THP-1 macrophages via TLR2. H37Ra was infected with THP-1(A) for one hour then treated with blocking anti-TLR2 antibody (AB9100, USA) for 30 min, and finally treated with ESAT-6 (5 μg/ml) for 24 h. Macrophages apoptosis was detected by Annexin V FITC/PI in flow cytometry. **B** Quantitative data of apoptosis in ESAT-6 and/or mycobacteria/AB9100 stimulated macrophages are shown. **C**, **D** ESAT-6 inhibits apoptosis of THP-1 macrophages via Caspase 9/Caspase3. The cells with the same treatment were analyzed by Western blotting for Casepase3, cleaved-Caspase3, Caspase9, and cleaved-Caspase9 protein levels. Densitometric quantification of representative blots relative to *β*-actin levels. Results are shown as mean ± SEM. *p < 0.05,**p < 0.01, and ***p < 0.001, respectively, compared with the *M. tb* group

Attenuated *M. tb* strains have been shown to induce higher levels of apoptosis in alveolar macrophages compared to their virulent counterparts [22]. ESAT-6, a protein secreted by the virulent H37Rv strain, is notably absent in the secretion profile of the attenuated H37Ra strain [23]. We investigated the effects of ESAT-6 and H37Ra on apoptosis in THP-1(A) cells using flow cytometry. Our results showed that *M. tb* infection significantly increased apoptosis in THP-1(A) cells compared to the untreated control group (Fig. 2A). However, when ESAT-6 (5 μg/ml) was added to the infected cells, this increase in apoptosis was notably reversed (Fig. 2A). Interestingly, a higher concentration of ESAT-6 (10 μg/ml) resulted in a higher apoptotic rate than the 5 μg/ml dose, suggesting potential cellular toxicity at elevated ESAT-6 levels (Fig. 2B). We also confirmed that ESAT-6 does not cause cytotoxicity in this concentration range (Supplementary Fig. S3), while can slightly induce apoptosis without *M. tb* challenge (Supplementary Fig. S4). *M. tb* infection also caused mitochondrial dysfunction, as evidenced by a decrease in mitochondrial membrane potential, a key marker of apoptosis (Fig. 2C). The addition of exogenous ESAT-6 significantly mitigated this reduction in mitochondrial membrane potential (Fig. 2D). To confirm that ESAT-6 inhibits apoptosis via TLR2, we used the selective TLR2 blocking antibody AB9100. Treatment with AB9100 significantly reversed the anti-apoptotic effect of ESAT-6 compared to the ESAT-6 group without the antibody (Fig. 3A, B). While AB9100 did not alter *M. tb*-modulated apoptosis without ESAT-6 (Supplementary Fig. S5). These findings confirm that ESAT-6 inhibits macrophage apoptosis through the TLR2 pathway.

### ESAT-6 inhibits *M. tb*-induced macrophage apoptosis via Caspase 9/Caspase 3

After confirming that ESAT-6 inhibits apoptosis in macrophages infected with *M. tb*, we further investigated its effects on apoptosis-related protein markers (Fig. 2E, F). Activation of Caspase 9 and Caspase 3, typically triggered by *M. tb*, was significantly reduced by treatment with 5 μg/ml ESAT-6 (Fig. 2E, G). Additionally, we assessed the levels of cleaved-PARP, Bcl-2, and BAX (Fig. 2F, H, I). Our results showed that ESAT-6 markedly inhibited the expression of apoptotic protein markers cleaved-PARP and BAX, while simultaneously inducing the expression of the anti-apoptotic marker Bcl-2 in THP-1(A) macrophages exposed to *M. tb*. Moreover, the use of TLR2-specific blocking antibodies partially inhibited the effects of ESAT-6 (Fig. 3C, D), confirming that the anti-apoptotic action of ESAT-6 operates via the Caspase 9/Caspase 3 pathway and involves TLR2 signaling in this process.

### ESAT-6 downgrades *M. tb*-induced TLR2 expression and macrophages’ phagocytosis

**Fig. 4:**
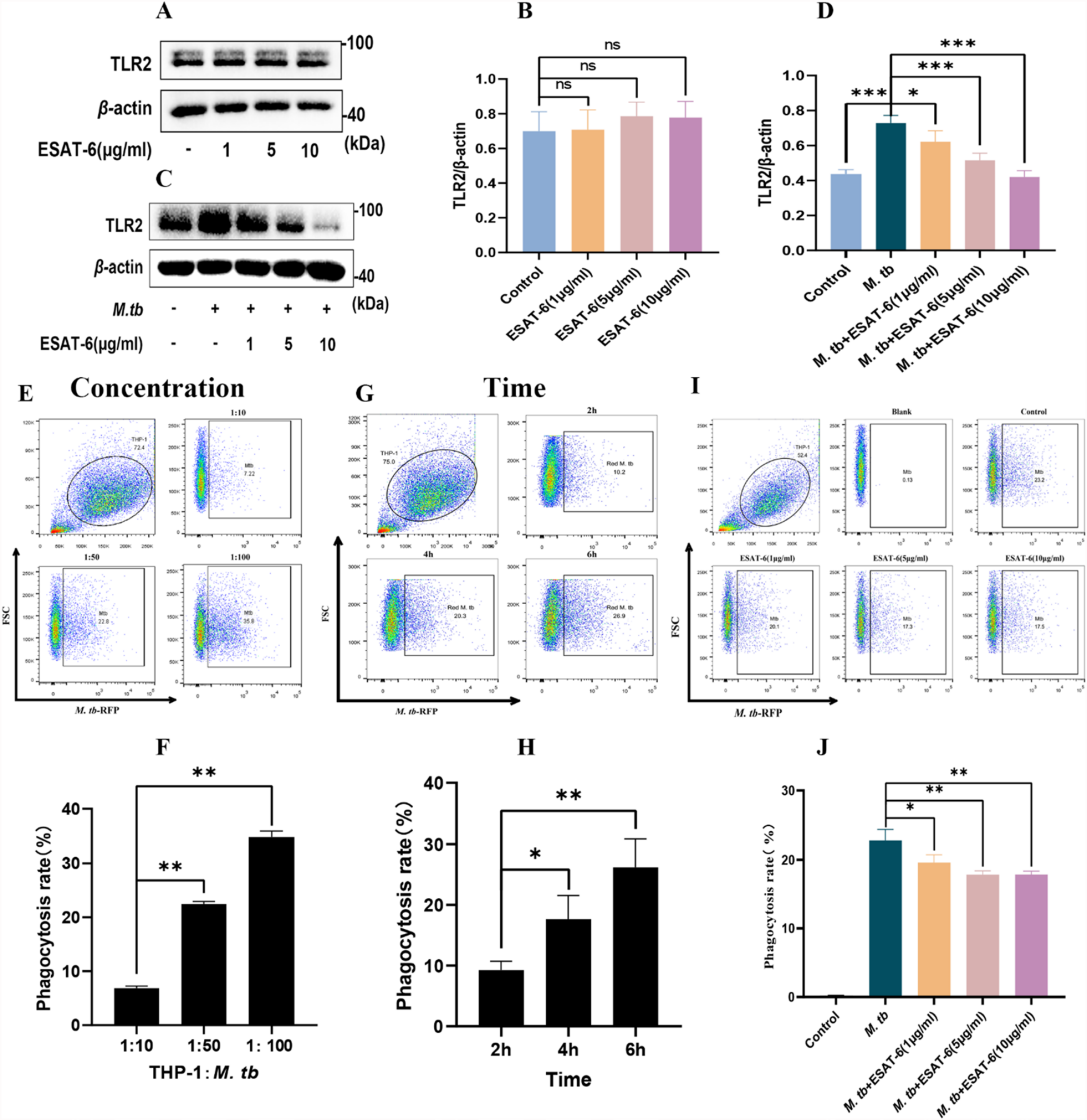
ESAT-6 inhibits TLR2 expression and phagocytosis of *M. tb* in THP-1 macrophage. **A**, **B** The effect of different concentrations of ESAT-6 alone on TLR2 protein levels in macrophages. THP-1(A) cells (1 × 10^6^) were co-incubated with different concentrations of ESAT-6 for 24 h, then total protein was extracted and TLR2 expression level was detected by WB. Densitometric quantification of representative blots were performed relative to *β*-actin levels. **C**, **D** ESAT-6 inhibits *M. tb*-induced expression of TLR2 in a dose-dependent manner. THP-1(A) cells (1 × 10^6^) were co-incubated with *M. tb* and different concentrations of ESAT-6 for 24 h. Densitometric quantification of representative blots were performed relative to *β*-actin levels. **E**, **F** The RFP-*M. tb* dose-dependent phagocytosis curve at four hours. **G**, **H** The RFP-*M. tb* time-dependent phagocytosis curve at MOI = 50. **I** THP-1(A) was co-incubated with different concentrations of ESAT-6 for 24 h followed by RFP-expressing H37Ra phagocytosis for 4 h. **J** Dose–response curves for EAST-6 inhibition of macrophage phagocytosis were examined by flow cytometry. Data are representative of at least three different experiments. Results are mean ± SEM. *p < 0.05, **p < 0.01 and ***p < 0.001

Macrophage phagocytosis could serve as an indirect indicator of the effectiveness of the body’s immune response [24]. Building on our findings that ESAT-6 inhibits macrophage apoptosis via TLR2, we further explored the effects of ESAT-6 on TLR2 protein expression and macrophage phagocytosis. Our results showed that ESAT-6 did not alter basal TLR2 expression levels (Fig. 4A, B). However, while *M. tb* significantly upregulated TLR2 expression, ESAT-6 inhibited this *M. tb*-induced TLR2 expression in a dose-dependent manner (Fig. 4C, D). Given that TLR2 is a crucial pattern recognition receptor in *M. tb* and plays a significant role in the phagocytic function of macrophages, we examined the impact of ESAT-6 on macrophage phagocytosis.

To do this, we constructed concentration- (Fig. 4E, F) and time-dependent (Fig. 4G, H) curves to determine the optimal conditions for phagocytosis assays, identifying an MOI of 50 and a 4-h post-infection window as the most suitable parameters (Fig. 4E–H). Under these conditions, our data demonstrated that ESAT-6 significantly inhibited macrophage phagocytosis in a dose-dependent manner (Fig. 4I, J). Based on these findings, we hypothesize that ESAT-6 impairs macrophage phagocytosis by downregulating TLR2 expression.

### ESAT-6 inhibits ROS and the killing of *M. tb* by macrophages

**Fig. 5:**
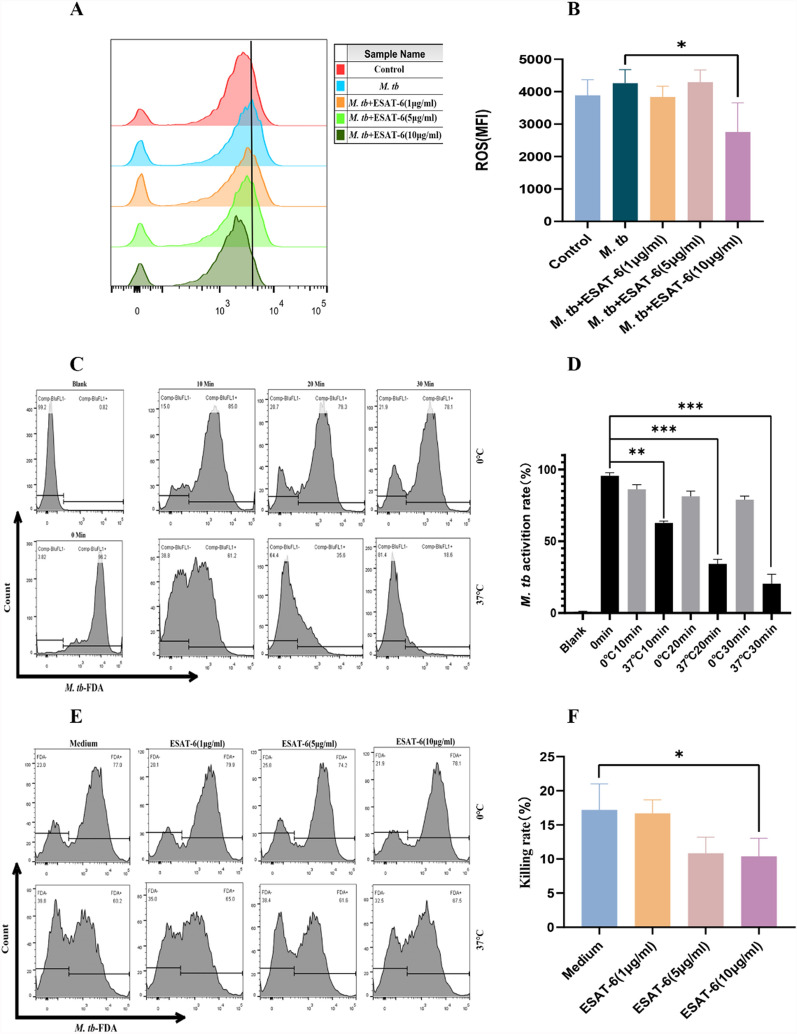
ESAT-6 reduced ROS production and decreased the killing function of THP-1(A). **A** ESAT-6 reduces ROS in *M. tb*-infected cells. THP-1(A) cells (1 × 10^6^) were co-incubated with *M. tb* and different concentrations of ESAT-6 for 4 h and then ROS levels were detected using DCFH-DA. Results were analyzed for MFI results using FlowJo. **B** Quantitative data of ROS in ESAT-6 and/or mycobacteria stimulated macrophages are shown. **C** Time and temperature normal kill curve of THP-1(A) cells. The 0 °C and 37 °C groups were set up at 10, 20 and 30 min. The 0 °C groups were used to eliminate the *M. tb* killed during the first 30 min of phagocytosis. **D** Quantitative data of Time and temperature normal kill curve of THP-1(A) cells are shown. **E** ESAT-6 significantly inhibited the killing function of THP-1(A) cells. **F** The histogram represents the percentage of fluorescence at 0 °C minus the percentage of fluorescence at 37 °C. Results are shown as mean ± SEM. *p < 0.05,**p < 0.01 and ***p < 0.001

During a defensive immune response, macrophages protect the host by limiting the growth of *M. tb* through the production of reactive oxygen species (ROS) during early infection [25]. Consistently, our results showed that *M. tb* infection slightly increased ROS production in THP-1(A) macrophages. However, ESAT-6 was found to significantly reduce ROS production in *M. tb*-infected THP-1(A) macrophages (Fig. 5A, B). We then investigated the bactericidal activity of macrophages in the presence of ESAT-6. As expected, THP-1(A) cells exhibited almost no killing activity at 0 °C compared to 37 °C (Fig. 5C, D). Our findings confirmed that macrophages killed *M. tb* in a time-dependent manner at 37 °C (Fig. 5C, D). The 0 °C group served as a control to eliminate any baseline killing rate of the cells against *M. tb* during the initial 30 min of phagocytosis. To evaluate the effect of ESAT-6 on macrophage bactericidal activity, we selected the optimal time point of 20 min. Our results indicated that ESAT-6 significantly downregulated the ability of macrophages to kill *M. tb* (Fig. 5E, F). These findings suggest that ESAT-6 suppresses host ROS production and bactericidal activity, thereby weakening the immune defenses against *M. tb*.

### ESAT-6 suppresses the production of IL-12, TNF-α, and IL-10 via TLR2

**Fig. 6:**
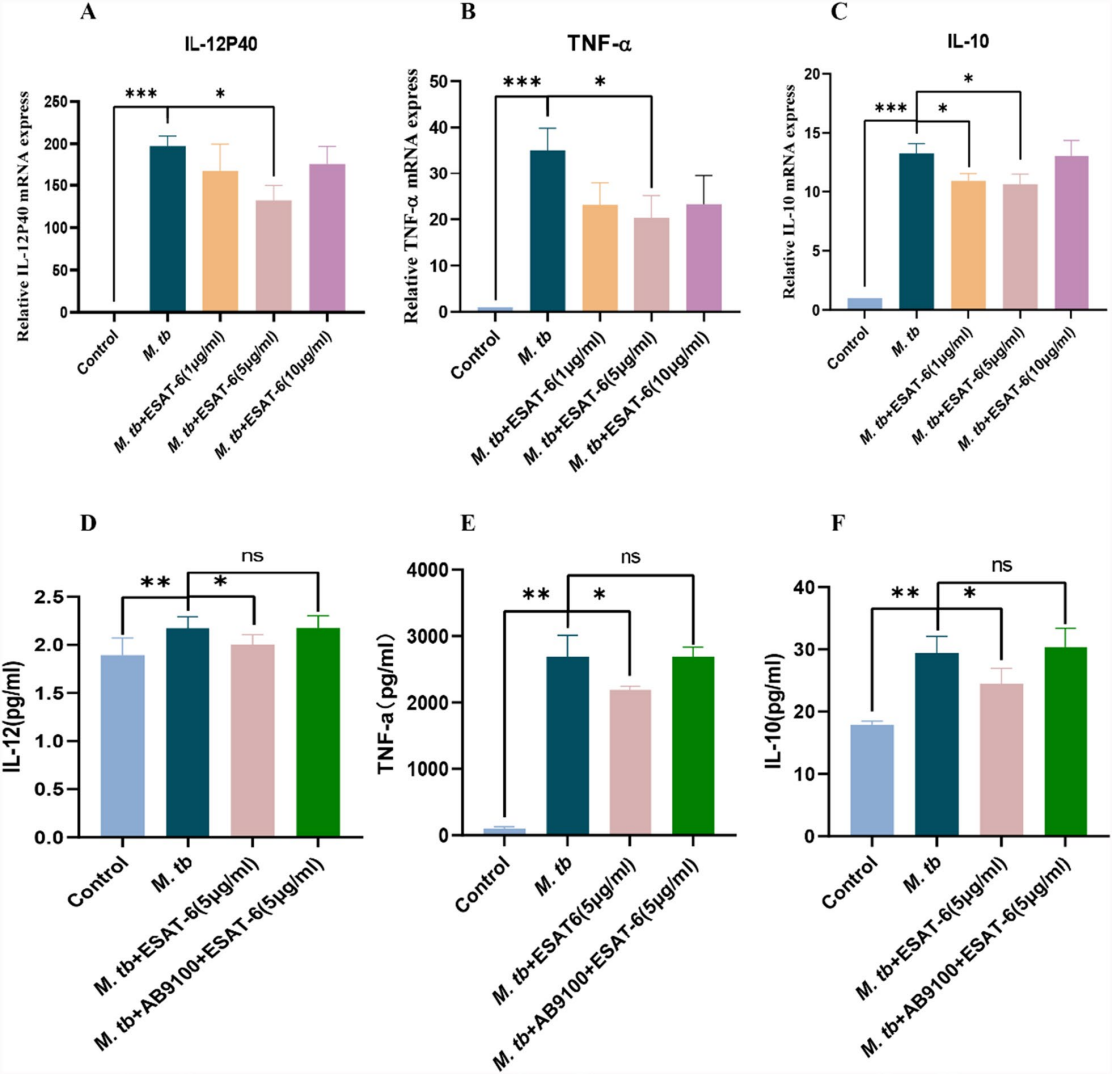
ESAT-6 inhibits the expression of cytokines IL-12, TNF-α, and IL-10 in THP-1 macrophages via TLR2. **A**–**C** Total RNA was extracted from THP-1(A) cells 24 h after infection of *M. tb*, *M. tb* with different concentrations of ESAT-6. mRNA levels of IL-12, TNF- α, and IL-10 were detected by real-time fluorescence quantitative PCR. **D**, **E** THP-1(A) cells were infected by H37Ra for one hour, then treated with blocking anti-TLR2 antibody for 30 min, and finally treated with 5 μg/ml ESAT-6 for 24 h. Protein levels of IL-12, TNF-α, and IL-10 were detected by ELISA. The experiments were repeated at least three times and the data represented in the figure are the mean ± SEM of three independent experiments. *, **, *** and **** indicate p < 0.05, p < 0.01, p < 0.001 and p < 0.0001, respectively, compared to the *M. tb*-infected group

We next investigated the regulatory effects of ESAT-6 on cytokine production in *M. tb*-infected THP-1(A) cells using RT-qPCR (Fig. 6A–C) and ELISA (Fig. 6D–F). Compared to the control group, *M. tb* infection significantly increased the transcription levels of IL-12p40, TNF-α, and IL-10. However, treatment with ESAT-6 (5 μg/ml) effectively countered these increases in both assays (Fig. 6). To determine whether ESAT-6 induces cytokine suppression via TLR2, we treated THP-1(A) cells with a TLR2-selective blocking antibody. As shown in Fig. 6D–F, ESAT-6 significantly suppressed the levels of IL-12, TNF-α, and IL-10. However, this suppressive effect was notably inhibited by the TLR2-selective blocking antibody AB9100. These results indicate that ESAT-6 suppresses the production of IL-12, TNF-α, and IL-10 in *M. tb*-infected THP-1(A) macrophages through a TLR2-dependent mechanism.

### ESAT-6-containing RD1 region in BCG affects host cell defense gene expression

By comparing human primary dendritic cells exposed to normal BCG with those exposed to engineered BCG containing a complemented RD1 region (BCGRD1), we identified 738 differentially expressed genes (DEGs) in the dataset GSE62423 (Supplementary Fig. S1A). After filtering (adj. P.Val < 0.05, |log(FC)|> 1), the number of DEGs was reduced to 338 (Supplementary Fig. S1B, C). The inclusion of the RD1 region significantly suppressed gene expression in host cells compared to normal BCG (Supplementary Fig. S1C). Notably, these DEGs were highly interactive with transcription factors involved in immune regulation, such as STAT1, IFIT1, and DDX58 (Supplementary Fig. S1D).

KEGG pathway analysis revealed that the RD1-driven DEGs had a substantial impact on defense-related pathways, including the NOD-like receptor pathway, Jak-STAT pathway, and TLR signaling pathway (Supplementary Fig. S1E). Additionally, these genes influenced tuberculosis-related pathways (Supplementary Fig. S1E). Gene ontology enrichment analysis indicated that the RD1 region affects host cell metabolism, GTPase-related signal transduction, and metallopeptidase activity (Supplementary Fig. S1F).

These findings underscore the central role of the RD1 region, which houses ESAT-6, in regulating immune functions within host cells. ESAT-6 appears to modulate host cell functions through established tuberculosis-related pathways, such as inhibiting macrophage apoptosis via the TLR2-mediated Caspase 9/Caspase 3 pathway (Supplementary Fig. S2). These results are consistent with our cell biology observations.

## Discussion

**Fig. 7:**
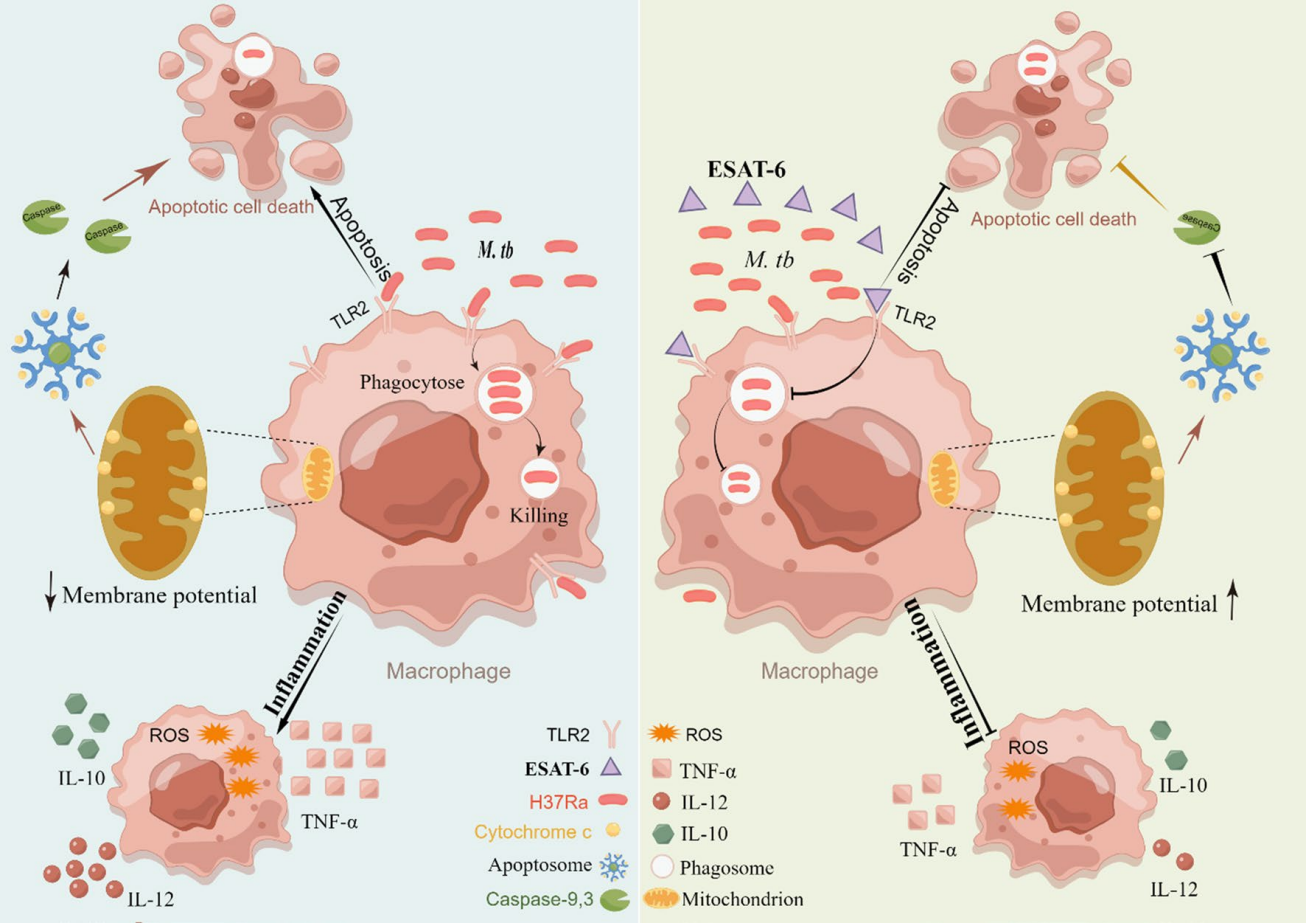
The schematic representation of ESAT-6 inhibited apoptosis, cytokines, phagocytosis and killing capacity of macrophages via TLR2

In this study, motivated by bioinformatic evidence suggesting that ESAT-6 plays a role in host defense against *M. tb* (Supplemental Figures S1-S2), we investigated the function of ESAT-6, a bacterial protein and virulence factor, in immune evasion and regulation within an in vitro model of *M. tb* immune interaction. Our findings revealed that ESAT-6 significantly inhibited host cell apoptosis, bacterial killing, and cytokine expression in THP-1(A) macrophages exposed to the H37Ra strain (Fig. 7). These effects were found to be primarily mediated through TLR2. In silico analyses indicated that ESAT-6, particularly the RD1 region, has a substantial impact on host cell immunity against tuberculosis. Consistent with this, our in vitro experiments confirmed that ESAT-6 suppresses apoptosis and reduces the expression of IL-10, TNF-α, and IL-12 in THP-1(A) macrophages via TLR2 signaling, confirmed by AB9100 (TLR2 antibody) and heat-inactivated ESAT-6 (Supplementary Fig. S6). Additionally, ESAT-6 was shown to inhibit apoptosis induced by H37Ra through the endogenous Caspase 9/Caspase 3 pathway. Our results revealed the selective role of ESAT-6 on macrophage immunity, potentially highlighting critical insights for tuberculosis vaccine development. Moreover, we applied varying concentrations of ESAT-6 to macrophages, observing that at an optimal concentration, ESAT-6 inhibited apoptosis even in the context of infection, suggesting a dose-dependent mechanism that modulates host–pathogen interactions. Lastly, our findings support the hypothesis that ESAT-6 assists the bacterium in immune escape by interfering with macrophage function, furthering its survival and persistence. Collectively, these results suggest that ESAT-6 facilitates the immune evasion of *M. tb* by inhibiting macrophage apoptosis, cytokine production, and phagocytic activity, thereby aiding in the pathogen’s survival and persistence (Fig. 7).

Macrophages are often regarded as the first line of defense against *M. tb* and are believed to facilitate the transport of the bacteria into the bloodstream [26]. Apoptosis of macrophages is a critical host defense mechanism against intracellular infections [27]. Our study demonstrated that ESAT-6 reduces *M. tb* H37Ra-induced apoptosis in THP-1(A) macrophages via TLR2. Western blotting revealed that ESAT-6 inhibits macrophage apoptosis through the Caspase-9/Caspase-3 pathway, aligning with our bioinformatics analysis. However, the literature presents a nuanced view of ESAT-6’s role. It has been shown that ESAT-6, a virulence factor secreted by the RD1 region of virulent *M. tb* strains, can induce apoptosis in host cells independently [28, 29]. For instance, ESAT-6 has been demonstrated to activate Caspase-9 and Caspase-3 in bone marrow-derived macrophages (BMDM) through TLR2 [29]. Additionally, Yang et al. reported that ESAT-6 induces macrophage apoptosis by targeting the miRNA155-SOCS1 pathway [30]. Aguilo. et al. demonstrated that ESAT-6 protein secreted by the ESX-1 system promotes apoptosis and facilitates bacterial delivery between macrophages in mice [43].

The discrepancies in these findings may arise from differences in experimental conditions. Unlike the isolated effects of *M. tb* on macrophages, our results show that an optimal dose of ESAT-6 inhibits *M. tb*-induced Caspase-9 and Caspase-3 expression. This may indicate that virulent strains of *M. tb*, which secrete ESAT-6, can significantly suppress macrophage apoptosis compared to attenuated strains that do not produce ESAT-6. Furthermore, the observed increase in apoptosis at higher concentrations of ESAT-6 in THP-1(A) macrophages may also contribute to these differences. As a note, in addition to the recombinant ESAT-6 used in the current study, a validation study with H37Rv-ΔRD1 strain, a variant of the virulent H37Rv strain that lacks the entire RD1 region, where ESAT-6 gene is located, will be beneficial to confirm a key role of ESAT-6 in cellular immunity by comparing to the wild-type H37Rv.

TLRs are crucial for bridging non-specific and specific immune responses, serving as key pattern recognition receptors (PRRs) [31]. The six amino acids at the C-terminus of ESAT-6 have been shown to interact with the extracellular domain (ECD) of TLR2 [5]. ESAT-6 stimulates TNF-α secretion from macrophages and IL-6 and TGFβ secretion from dendritic cells through TLR2 [29]. In our study, we observed a significant increase in TLR2 expression in macrophages infected with *M. tb*, indicating that macrophages upregulate TLR2 to enhance recognition of the pathogen. However, ESAT-6 was found to inhibit this upregulation, suggesting that ESAT-6 downregulates TLR2 expression, thereby impairing macrophage differentiation and potentially contributing to immune evasion.

Macrophages are specialized antigen-presenting cells (APCs) with phagocytosis being one of their most critical functions [32]. *M. tb* typically enters alveolar macrophages through either receptor-mediated or non-specific cytocytosis. Our previous research demonstrated that TLR4 plays a crucial role in the phagocytic activity of THP-1(A) cells [33]. In this study, we observed that ESAT-6 significantly inhibited macrophage phagocytosis in a dose-dependent manner when compared to the *M. tb* control group. Interestingly, this inhibition coincided with a suppression of *M. tb*-induced TLR2 expression by ESAT-6. We hypothesize that ESAT-6 facilitates immune evasion by inhibiting TLR2 expression, thereby reducing macrophage recognition and phagocytic activity.

Reactive oxygen species (ROS) and nitric oxide (NO) are critical components of the host’s antimicrobial defense mechanisms, particularly in macrophages [34]. ROS play a key role in controlling bacterial growth during intracellular infections by oxidizing the cysteine -SH moieties in bacterial proteins [35]. Some studies have shown that ESAT-6 can suppress LPS-induced NF-κB-dependent gene expression by inhibiting ROS production [35]. In our research, we found that ROS levels were elevated in *M. tb*-infected macrophages, contributing to the killing of engulfed bacteria. However, the control group also exhibited high baseline ROS levels, which we speculate might be due to the pre-induction of THP-1 cells with PMA or the use of an attenuated rather than a virulent strain of *M. tb*. ESAT-6 was found to reduce *M. tb*-induced ROS generation in macrophages, which appears to be crucial for the bacteria’s survival by preventing macrophage-mediated killing. Our killing assay confirmed that ESAT-6 inhibits the bactericidal activity of macrophages, highlighting its critical role in the immune evasion of *M. tb*.

Macrophages are generally categorized into two subtypes: (1) classically activated macrophages (M1-type), which are pro-inflammatory and secrete cytokines like IL-12 and TNF-α, and (2) alternatively activated macrophages (M2-type), known for their immunomodulatory and anti-inflammatory functions, producing TGF-β and IL-10 [36]. Animal studies have shown that IL-12 is essential for controlling *M. tb* infection, as its absence allows the pathogen to proliferate unchecked [37]. Several studies suggest that ESAT-6 can inhibit the LPS-induced production of IL-12p40 [38]. Meanwhile, Pathak et al. also separately confirmed that there is ESAT-6 binding specifically to TLR2 for this cause [38]. This is consistent with our results. In our research, we observed a significant increase in cytokine levels in the *M. tb*-infected group, with TNF-α showing the most pronounced elevation. We hypothesize that TNF-α, as an exogenous activator of apoptosis, may play a role in the apoptosis induced by *M. tb*. However, ESAT-6 was found to suppress the *M. tb*-induced expression of IL-12, TNF-α, and IL-10 via TLR2, indicating that ESAT-6 can dampen the immune response of macrophages [39, 40]. The inhibition of TNF-α expression by ESAT-6 may represent an additional mechanism by which it prevents macrophage apoptosis. Notably, ESAT-6 alone has been shown to significantly elevate the levels of IL-10, TNF-α, and IL-12 in macrophages. This may explain the increased expression of these cytokines in the high-dose ESAT-6 group in our study. Overall, we propose that ESAT-6 evades host immune defenses by interfering with macrophage polarization, a mechanism that highly pathogenic *M. tb* strains may exploit to suppress macrophage immune activity, thereby ensuring their continued intracellular survival and growth.

In summary, this study is the first to demonstrate that ESAT-6, a protein derived from *M. tb*, suppresses host innate immunity by inhibiting key macrophage functions, including apoptosis, ROS production, phagocytosis, and bactericidal activity. These findings pave the way for further research into the various immunoregulatory mechanisms of ESAT-6 in host defense against *M. tb* and provide new insights into how *M. tb* evades the immune system.

## Materials and methods

### ESAT-6 protein and other reagents

Lyophilized powder of the *M. tb* ESAT-6 virulence protein was obtained from Shanghai Gene-Optimal (Shanghai, China). PMA (#P1585) was purchased from Sigma-Aldrich (Shanghai, China). The apoptosis kit was procured from Beyotime (Shanghai, China). Target and GAPDH gene primers were procured from Shanghai Sangon (China), and RT-qPCR kits were obtained from Beijing TransGen Biotech (Beijing, China). The reactive oxygen species (ROS) synthesis kits were procured from Solarbio (Beijing, China). Primary antibodies against BAX and Bcl2 were procured from Proteintech (Wuhan, China). The Caspase3 primary antibodies and TLR2-specific blocking antibody (AB9100) were procured from Abcam (Cambridge, UK). All additional antibodies were obtained from Cell Signaling Technology (CST, USA). The ladder of the western blot was purchased from Epizyme Biotech (CAT: WJ103, Lot:026951000) (Shanghai, China). Trizol reagent was purchased from ThermoFisher (Waltham, MA, USA). Mitochondrial Membrane Potential Assay Kit (JC-1) was obtained from MedChemExpress(Shanghai, China).

### Cell and bacterial cultures

THP-1 cells were obtained from ATCC (Manassas, USA) and cultured in RPMI 1640 medium (Sigma-Aldrich, Shanghai, China) supplemented with 10% fetal bovine serum (FBS), penicillin, and streptomycin. The cells were grown in 6-well culture plates until they reached a density of 1 × 10^6^ cells/ml. To induce macrophage-like differentiation, the cells were stimulated with PMA for 24 h, and these differentiated cells are referred to as THP-1(A) cells [41]. The H37Ra strain of *M. tb* was acquired from the Chinese Medical Bacterial Conservation and Management Center (Beijing, China; lot number: 9302025) and cultured in 7H9 + OADC liquid medium. The RFP-expressing H37Ra strain, containing the kanamycin resistance gene, was generously provided by Prof. Boqing Li from the Anhui Province Key Laboratory of Basic and Clinical Immunology of Chronic Diseases. This strain was cultured in 7H10 + OADC medium with 100 μg/ml kanamycin. For the phagocytosis assay, an MOI of 50:1 (RFP-H37Ra: THP-1(A)) was used to infect the cells, while an MOI of 10:1 was applied for the remaining experiments.

### Apoptosis detection

To assess apoptosis, THP-1(A) cells (1 × 10^6^) were incubated with three concentrations of ESAT-6 protein (1, 5, and 10 μg/ml) in the presence of *M. tb* for 24 h. The cells were then harvested using trypsin digestion without EDTA, washed with chilled phosphate-buffered saline (PBS), and resuspended in 195 μl of buffer. To stain the cells, 5 μl of Annexin V-FITC and 5 μl of propidium iodide (PI) were added in a light-protected environment. Apoptosis was subsequently measured using DxP Athena flow cytometry (CYTEK, USA), and the apoptosis rate was analyzed using FlowJo software (version FlowJo vX.0.7).

### Mitochondrial staining and Zeiss immunofluorescence microscope

For mitochondrial membrane potential analysis, the cells were washed twice with PBS and resuspended with basal medium containing JC-1 (2 μM), followed by incubation in warm water bath (37℃) for 20 min in a light-proof environment. THP-1(A) cells were further washed with fresh medium to remove excess probes and observed using a Zeiss immunofluorescence microscope (Observer Z1, ZEISS, Deutschland). Images were analyzed and quantified using Image J software (Maryland, USA).

### TLR2 blocking experiments

Macrophages were infected with *M. tb* for one hour and then treated with the blocking anti-TLR2 antibody (2 μg/ml) for 30 min. Afterward, ESAT-6 (5 μg/ml) was added to the medium, and the cells were incubated for 24 h to assess apoptosis as previously described. The supernatants were then collected for ELISA analysis. The IgG control (rabbit) of AB9100 was examined with no effects on ESAT-6 induced apoptosis (Supplementary Fig. S7).

### Immunoblot detection

The cells were lysed using RIPA buffer (high-strength) to extract total protein. To assess the expression levels of target proteins, the proteins were separated via electrophoresis, initially at 80 V for the stacking gel and then at 110 V for the resolving gel for a total duration of 1 h. The separated proteins were transferred onto polyvinylidene fluoride (PVDF) membranes (Millipore, Sigma) at 200 mA for 2 h. The PVDF membranes were then blocked in 5% skim milk (w/v) for 3 h. Following blocking, the membranes were incubated with the primary antibody solution for 14 h at 4 °C with constant shaking. Afterward, the membranes were washed three times with TBS-T buffer (0.5% Tween-20 in TBS) and then incubated with the secondary antibody solution for 2 h. The membranes were again washed three times with TBS-T (5 min per wash) and then treated with Chemiluminescent HRP Substrate (Millipore, Merck, USA) for 1 min. Finally, the target proteins were visualized and quantified using the Molecular Imager ChemiDoc XRS + Imaging System, and the grayscale values of the protein bands were analyzed using ImageJ software.

### Phagocytosis assay

THP-1(A) cells were first co-cultured with 1, 5, or 10 μg/ml of ESAT-6 for 24 h. Following this incubation, the cells were infected with RFP-expressing *M. tb* at a ratio of 50:1 (bacteria to THP-1(A) cells) for 4 h and then maintained in antibiotic-free medium at 37 °C. After the experiment, the THP-1(A) cells were thoroughly washed with chilled PBS containing 50 µg/ml gentamicin to remove any non-engulfed bacteria. The cells were then collected and the percentage of phagocytosis was determined using a DxP Athena flow cytometer (CYTEK, USA). For gating events, the cut-off point for phagocytosis was established using the uninfected control group.

### ROS detection

To quantify ROS levels in the cells, a ROS test kit was used. THP-1(A) cells (1 × 10^6^) were co-incubated with 1 μg/ml, 5 μg/ml, or 10 μg/ml of ESAT-6 and *M. tb* for four hours. The cells were then washed three times with chilled basal medium. After resuspending the cells in 300 μl of PBS, they were treated with 10 nM 2′,7′-dichlorofluorescein diacetate (DCFH-DA) and incubated in an icebox for 20 min at 37 °C. ROS levels were subsequently measured using a DxP Athena flow cytometer (CYTEK, USA), and the mean fluorescence intensity (MFI) of ROS was analyzed using FlowJo software (version Vx.0.7).

### Killing activity of macrophage

First, *M. tb* (1 × 10^9^/ml) was labeled with 1 μg/ml FDA in water at 37 °C for 30 min, resulting in *M. tb*-FDA with a labeling rate greater than 90%. *M. tb*-FDA was then used to infect THP-1(A) cells at an MOI of 50 for 30 min to allow phagocytosis. Following infection, the cells were washed twice with chilled PBS containing 50 µg/ml gentamicin to remove any unengulfed bacteria. The cells were then centrifuged at 800 rpm for 5 min and resuspended in 100 μl of complete RPMI 1640 medium per tube. The macrophages, now containing *M. tb*-FDA, were incubated at 37 °C for 0, 10, 20, or 30 min. To lyse the cells and release the intracellular bacteria, 300 μl of ice-cold ultrapure water was added to each tube. The samples were then analyzed using flow cytometry, and fluorescence percentages were calculated with FlowJo software to generate a time-kill curve for macrophages. The optimal time point for killing was determined from this curve.

To evaluate the effect of ESAT-6 on macrophage-killing activity, macrophages were stimulated with varying concentrations of ESAT-6 for 24 h. Following stimulation, the cells were washed twice with complete RPMI 1640 medium and resuspended in antibiotic-free medium. *M. tb*-FDA was then used to infect THP-1(A) cells at an MOI of 50 for 30 min to allow phagocytosis. The same *M. tb*-FDA infection procedure was repeated. After infection, cells were divided into two groups and incubated at 0 and 37 °C for 20 min. To release intracellular *M. tb*, 300 μl of ice-cold ultrapure water was added to each well for 2 min. The killing rate was then calculated using the formula: Killing rate = live *M. tb*% (0 °C) − live *M. tb*% (37 °C) [42].

### Total RNA extraction and RT-qPCR

To measure the levels of TNF-α, IL-12p40, and IL-10, total RNA was extracted from each cell group using TRIzol. The RNA concentration and purity were immediately assessed using the NanoVue Plus UV nucleic acid and protein detector. RNA samples with appropriate concentration and purity were selected for further analysis. The RNA was then reverse transcribed into cDNA using RT-qPCR kits. Following this, the cDNA was analyzed by qPCR to determine the Ct values for each sample. The relative expression levels were calculated using the 2^−ΔΔCt^ method. Specific primers for the human housekeeping gene GAPDH, as well as for the cytokines IL-12p40, TNF-α, and IL-10, were designed and purchased from Sangon. The primers used are detailed in the table below (Table 1).

**Table 1:** Target genes and housekeeping gene primer sequences

Gene	Primer Sequence (5′–> 3′)
IL-12p40	Forward: CCTGCCCAGAGCAAGATGTG Reverse: AGTTCCCATATGGCCACGAG
TNF-α	Forward: TGCACTTTGGAGTGATCGGC Reverse: GCTTGAGGGTTTGCTACAACA
IL-10	Forward: AACAAGAGCAAGGCCGTGGA Reverse:GAAGATGTCAAACTCACTCATGGC
GAPDH	Forward: TGCACCACCAACTGCTTAGC Reverse: GGCATGGACTGTGGTCATGAG

### ELISA detection

First, the wells of a 96-well ELISA plate were coated, washed, and blocked according to the manufacturer’s instructions (Bio-Techne, R&D Systems). Next, an appropriate volume of cell supernatant was added to the wells as specified by the manufacturer to measure the concentration of various cytokines. If the cytokine concentration in the sample exceeded the detection range, the supernatant was diluted with PBS. Absorbance values were then measured at 450 nm using the Multiskan Sky microplate reader (Thermo Fisher, USA). Cytokine concentrations in each sample were determined by comparing the absorbance values to a standard curve.

### Statistical analysis

The results of each group of experiments are presented using the mean and standard error of the mean (SEM). The computational analysis and visualization of the results were performed using by GraphPad Prism 8.3.0. Statistical significance was determined when the P value was less than 0.05.

### Bioinformatics

The NCBI GEO database (https://www.ncbi.nlm.nih.gov/geo/) was searched for human gene expression datasets with specific criteria: microarray datasets related to ESAT6, *M. tb*, macrophages, or dendritic cells. The GSE62423 dataset was selected, which involved BCG-infected dendritic cells, and the data was divided into control and experimental groups to assess BCG insertion in the RD1 region. Differential gene analysis was conducted using GEO2R, filtering for differentially expressed genes with an adjusted p-value < 0.05 and |log(FC)|> 1. These differentially expressed genes were then analyzed for gene ID conversion (https://www.uniprot.org/), Gene Ontology (GO) enrichment (http://geneontology.org/), and Kyoto Encyclopedia of Genes and Genomes (KEGG) gene enrichment (https://www.kegg.jp/kegg/). Protein–protein interaction networks were constructed using STRING (https://cn.string-db.org/) and visualized with Cytoscape software. Heat maps were generated using http://www.bioinformatics.com.cn.

## Conclusions

In conclusion, this study demonstrates that the *M. tb*-derived virulence factor ESAT-6 impairs host innate immunity by inhibiting macrophage apoptosis, ROS production, phagocytosis, and bactericidal activity. These findings provide new insights into the various immunoregulatory mechanisms of ESAT-6 and its role in *M. tb*’s evasion of host immune defenses, highlighting potential areas for further research on how this pathogen subverts the host immune response.

## Supplementary Information

Additional file 1.

Additional file 2.

## Data Availability

All original research data supporting reported results can be available upon request. The unedited parent image that support the findings of this study are openly available in Science Data Bank at https://www.scidb.cn/en/s/a6zMry. This article has been shown in a pre-print (https://www.authorea.com/doi/full/10.22541/au.167963771.11949216/v1).
