# Supplementary material for: Early secretory antigen target of 6-kDa of Mycobacterium tuberculosis inhibits macrophage apoptosis and host defense via TLR2
Source: Respir Res. 2025 Apr 9;26:131. doi: 10.1186/s12931-025-03210-z (PMC11983766; doi:10.1186/s12931-025-03210-z)
Supplement: Supplementary file 1 — Additional file 1. [file 12931_2025_3210_MOESM1_ESM.docx]

**B**

**A**


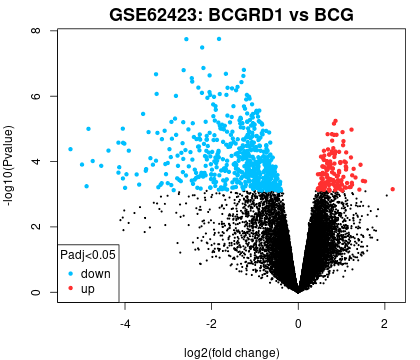

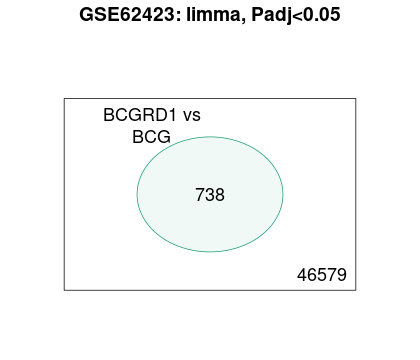

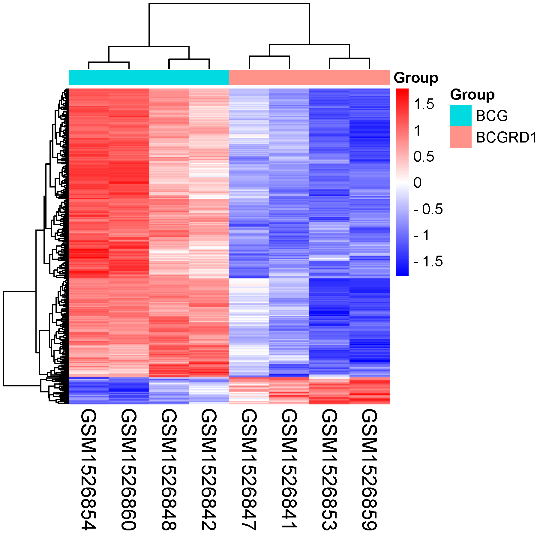


**C**

**D**


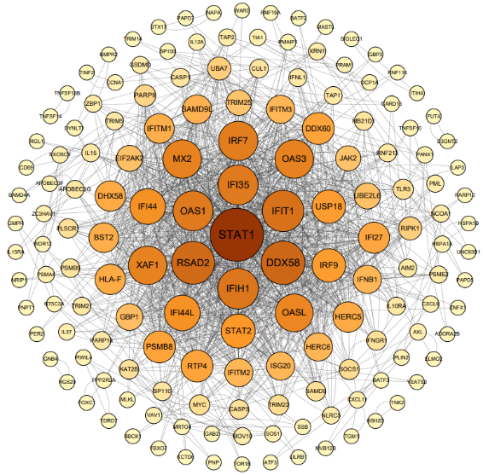

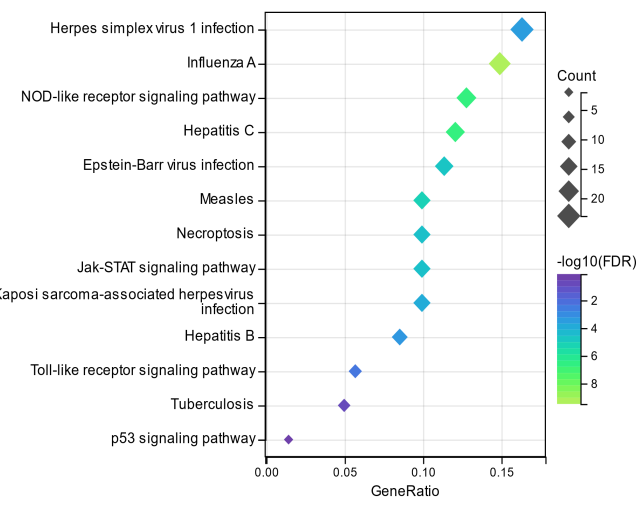

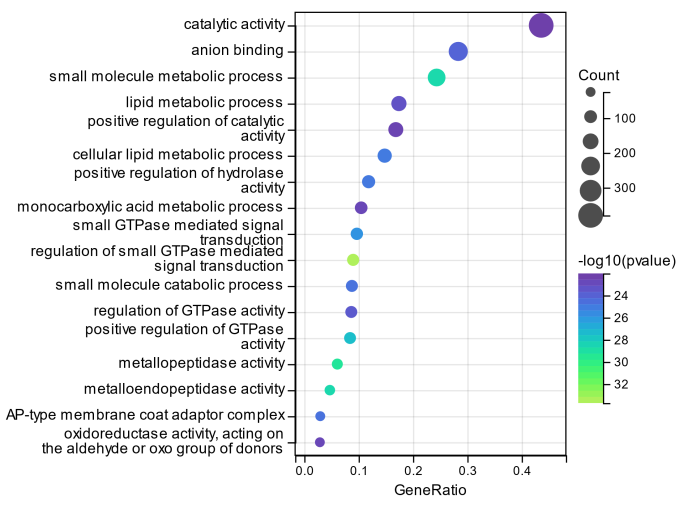


**E**

**F**

**Supplemental Figure S1**. Graph of the analysis results of the GSE62423 dataset. **A.** Venn diagram. **B**. Volcano plot. C. Heat map. **D** PPI prediction analysis plot. **E**. KEGG signaling pathway enrichment analysis plot. A red-marked signaling pathway is the target signal. **F**. GO enrichment analysis plot.


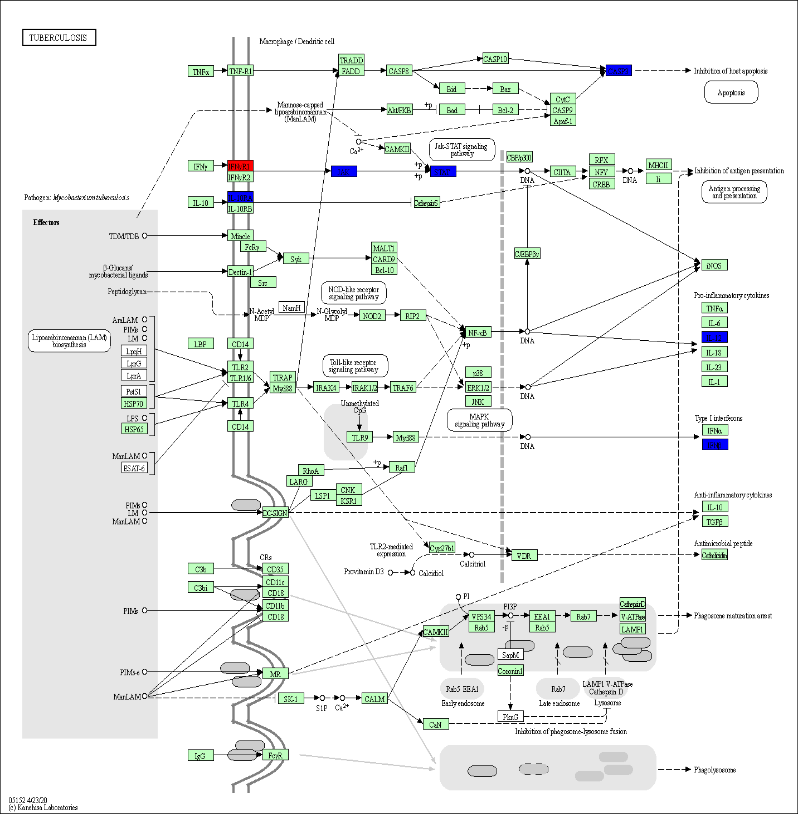


**Supplemental Figure S2**. Tuberculosis signaling pathway map. The orange arrow is the target signaling pathway

**
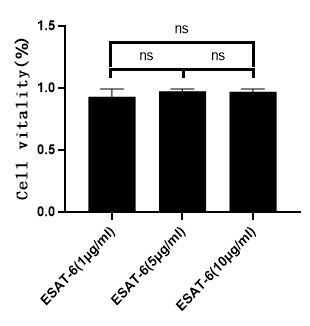
**

**Supplementary Figure S3**. The cytotoxic effects of recombinant ESAT-6 protein on THP-1 macrophages. Cell viability was assessed by a standard CCK-8 assay. The experiments were repeated at least three times and the data represented in the figure are the mean ± SEM of three independent experiments. NS: no significance.


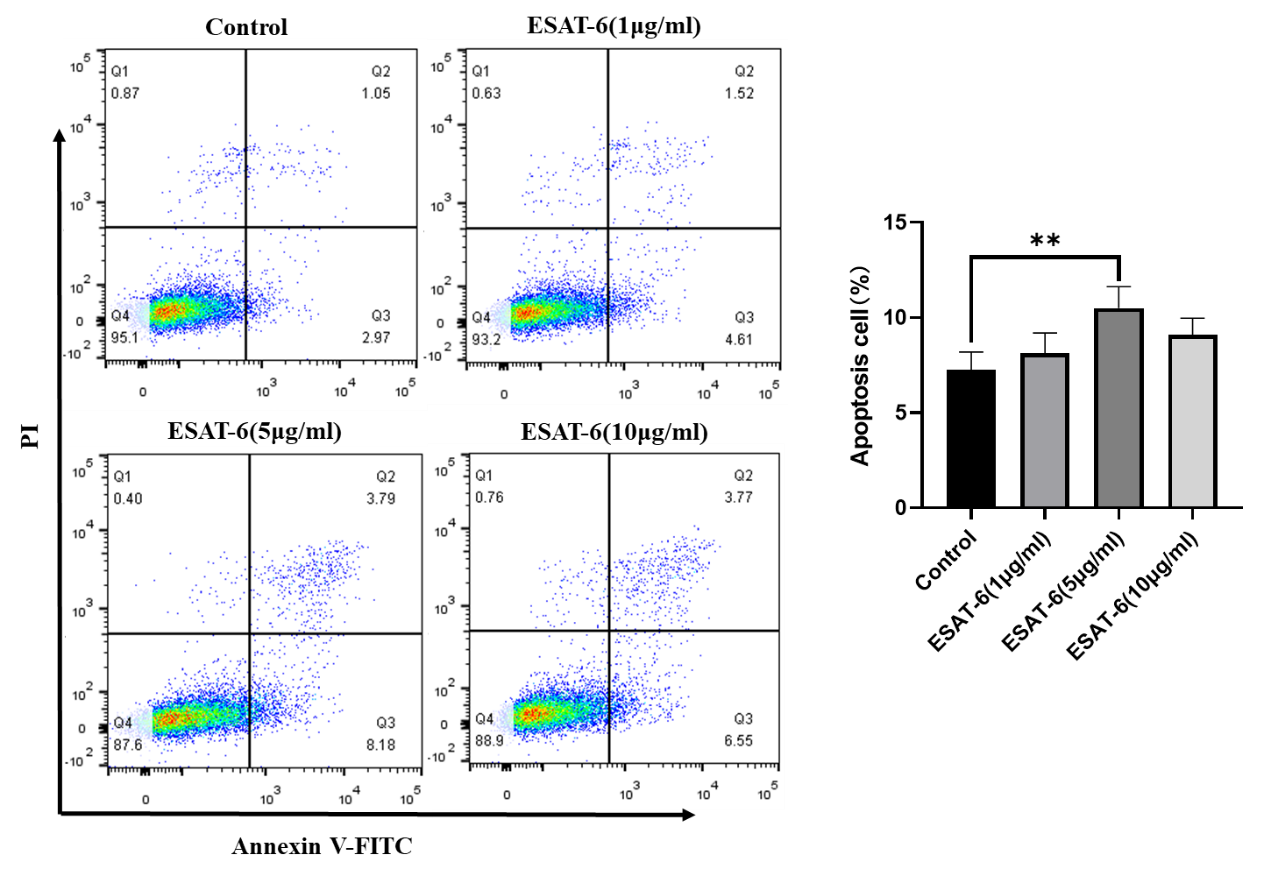


**Supplementary Figure S4.** The effects of Recombinant ESAT-6 protein on apoptosis of THP-1 macrophages. The experiments were repeated at least three times and the data represented in the figure are the mean ± SEM of three independent experiments. * *p* < 0.05, ** *p*<0.01 and *** *p* < 0.001.


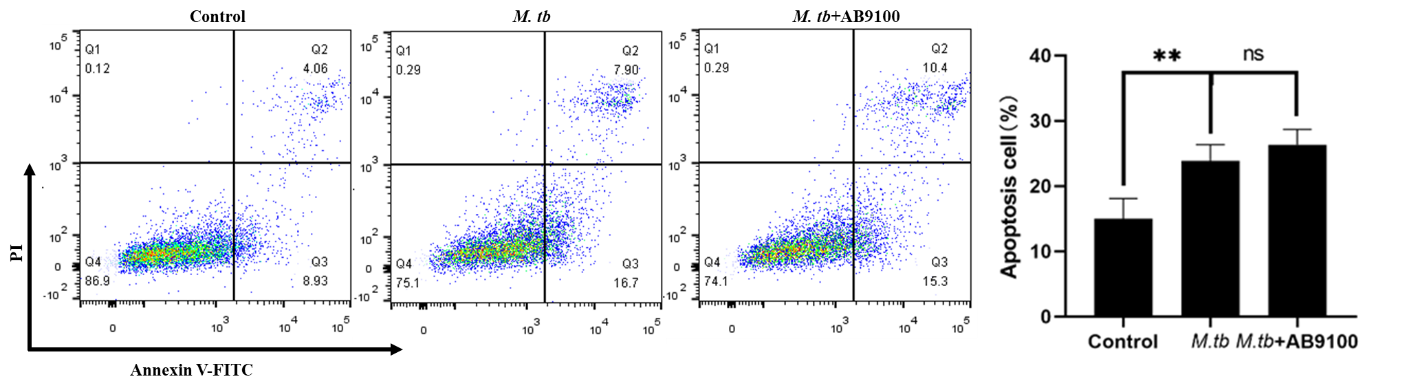


**Supplementary Figure S5.** The Effect of the blocking anti-TLR2 antibody (AB9100) on Apoptosis of THP-1 Macrophages. The experiments were repeated at least three times and the data represented in the figure are the mean ± SEM of three independent experiments. * *p* < 0.05, ** *p*<0.01 and *** *p* < 0.001. ns: no significance.


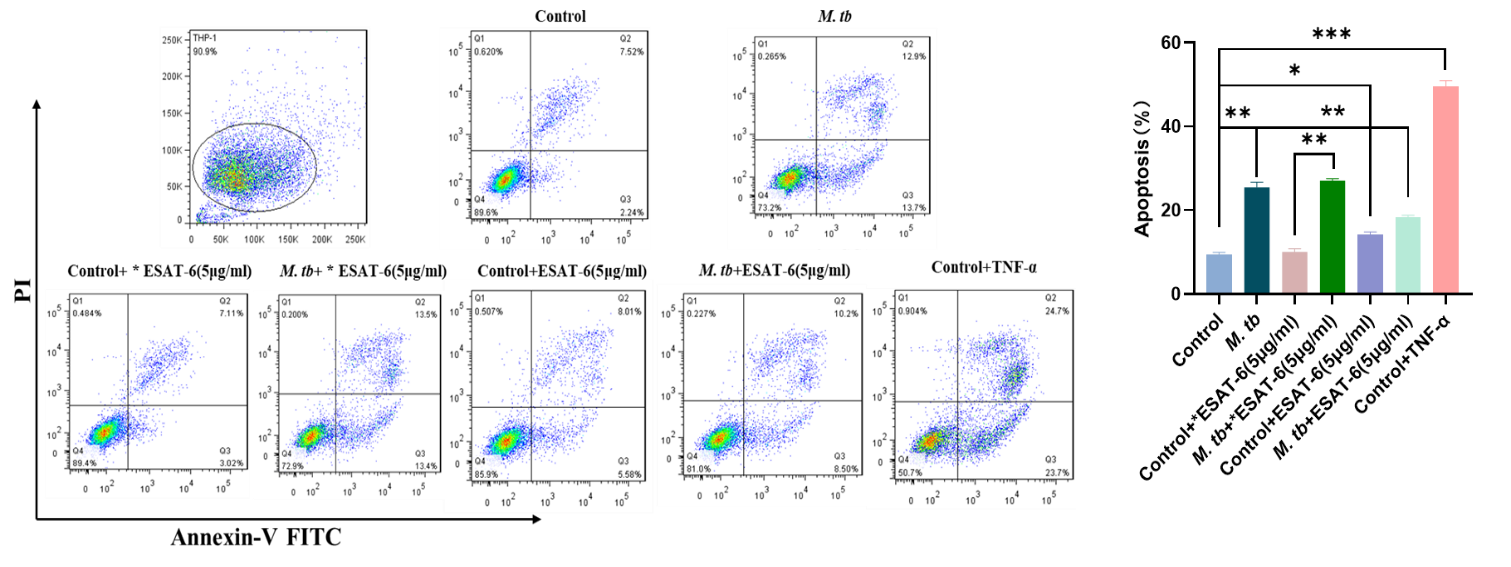


**Supplementary Figure S6.** The Effect of M. tb, inactivated recombinant ESA-6 protein [*ESAT-6, (5μg/ml)], recombinant ESAT-6 protein (5μg/ml), M. tb + inactivated recombinant ESA-6 protein [*ESAT-6, (5μg/ml)], M. tb + recombinant ESA-6 protein(5μg/ml), and TNF-α on macrophage apoptosis. The experiments were repeated at least three times and the data represented in the figure are the mean ± SEM of three independent experiments. * *p* < 0.05, ** *p*<0.01 and *** *p* < 0.001.

*M. tb*, inactivated recombinant ESA-6 protein [*ESAT-6, (5μg/ml)], recombinant ESAT-6 protein(5μg/ml), *M. tb* + inactivated recombinant ESA-6 protein [*ESAT-6, (5μg/ml)], and *M. tb* + recombinant ESA-6 protein (5μg/ml) were tested for their effects on macrophage apoptosis after acting on THP-1 macrophages (1×10^6^) for 24 h, respectively. Among them, a stronger exogenous apoptosis inducer, TNF-a (400ng/ml,48h) was used as a positive control. About 1×10^6^ cells were obtained by digesting with trypsin without EDTA and washing twice with cold PBS. After resuspending with 500 μl binding buffer, the cells were stained with 5 μl Annexin-V-FITC and 5 μl PI at 37°C in the dark for 15 minutes. Finally, cell apoptosis was measured by DxP Athena flow cytometer (CYTEK, USA).


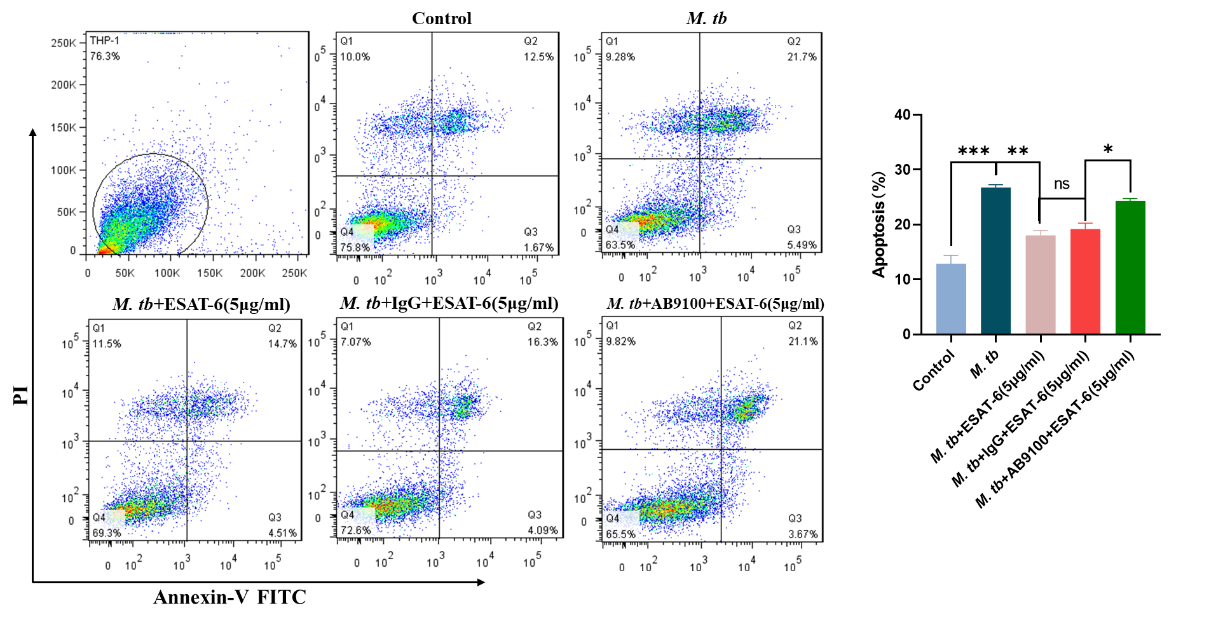


**Supplementary Figure S7. The isotype control experiment of anti-TLR2 antibody (AB9100).** H37Ra was infected with THP-1(A) for one hour then treated with blocking anti-TLR2 antibody (AB9100) for 30 minutes, and finally treated with ESAT-6 (5 μg/ml) for 24 hours. In parallel, the rabbit IgG antibody was used as an isotype control for AB9100 (2 μg/ml). Macrophages apoptosis was detected by Annexin V FITC/PI in flow cytometry. The experiments were repeated at least three times and the data represented in the figure are the mean ± SEM of three independent experiments. * *p* < 0.05, ** *p*<0.01 and *** *p* < 0.001. ns: not significant.
