# Supplementary material for: Early secretory antigen target of 6-kDa of Mycobacterium tuberculosis inhibits macrophage apoptosis and host defense via TLR2
Source: Respir Res. 2025 Apr 9;26:131. doi: 10.1186/s12931-025-03210-z (PMC11983766; doi:10.1186/s12931-025-03210-z)
Supplement: Supplementary file 2 — Additional file 2. [file 12931_2025_3210_MOESM2_ESM.zip › WB RAW DATA - ╕▒▒╛/Original Images for Blots.docx]

Figure 2E


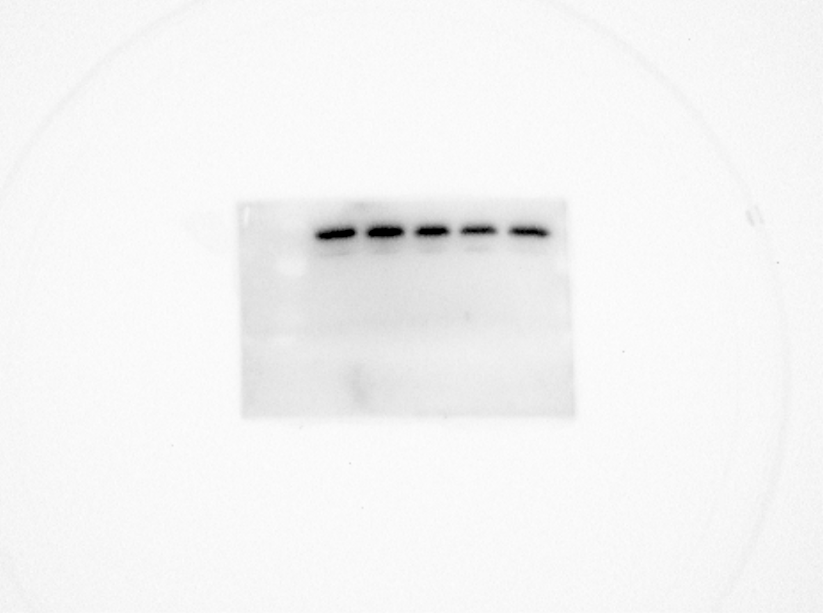

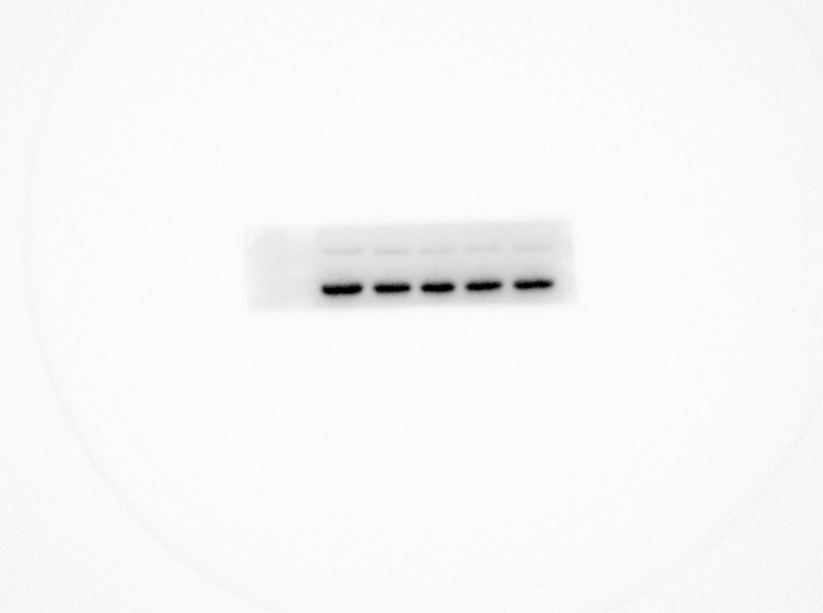


*M. tb+*E6(5μg/ml*)*

*M. tb+*6(10μg/ml)

*M. tb+*E6(1μg/ml)

*M. tb*

Control

*M. tb+*E6(5μg/ml*)*

*M. tb+*6(10μg/ml)

*M. tb+*E6(1μg/ml)

*M. tb*

Control

Caspase3

*β*-Actin


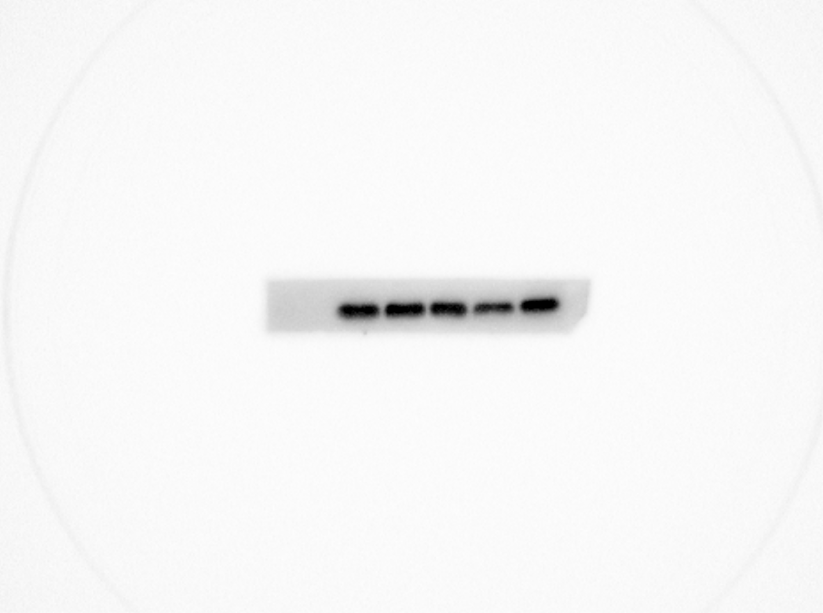

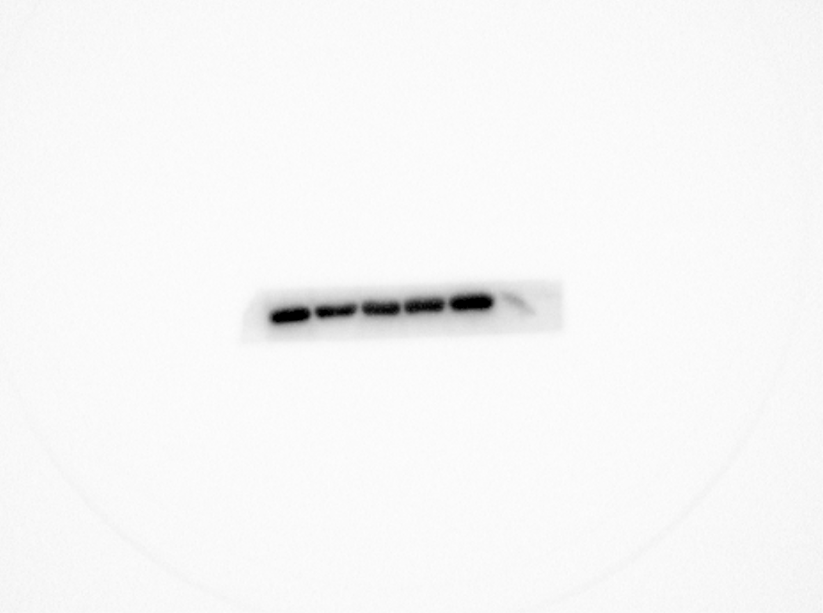


*M. tb+*E6(5μg/ml*)*

*M. tb+*6(10μg/ml)

*M. tb+*E6(1μg/ml)

*M. tb*

Control

*M. tb+*E6(5μg/ml*)*

*M. tb+*6(10μg/ml)

*M. tb+*E6(1μg/ml)

*M. tb*

Control

*β*-Actin

Caspase9


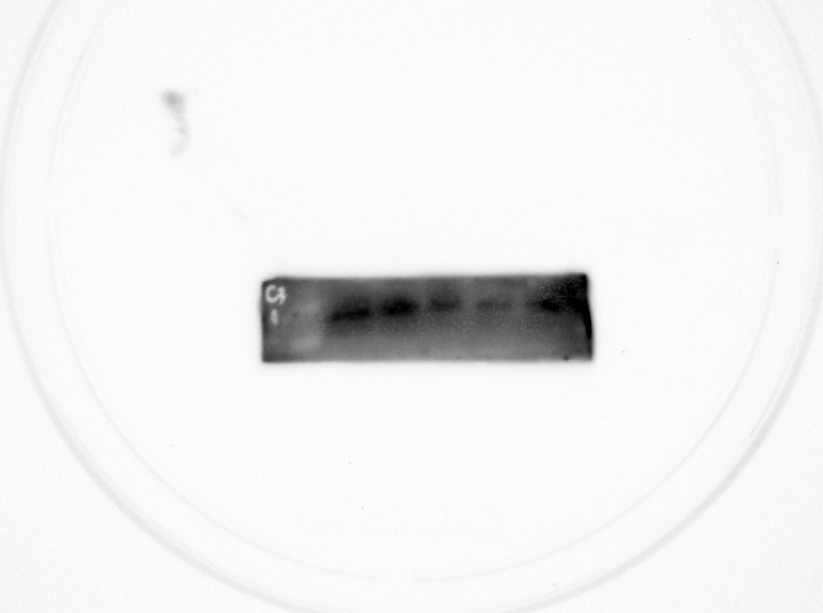

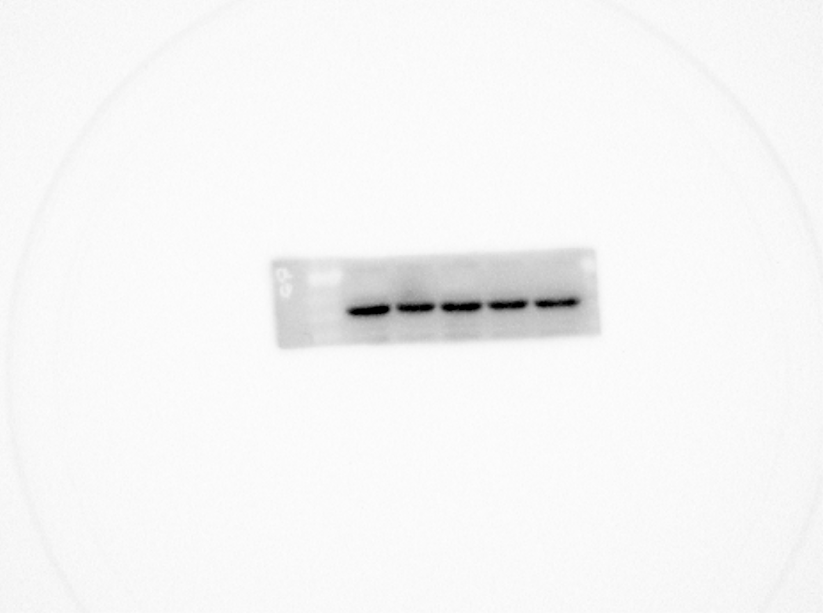


*M. tb+*E6(5μg/ml*)*

*M. tb+*6(10μg/ml)

*M. tb+*E6(1μg/ml)

*M. tb*

Control

*M. tb+*E6(5μg/ml*)*

*M. tb+*6(10μg/ml)

*M. tb+*E6(1μg/ml)

*M. tb*

Control


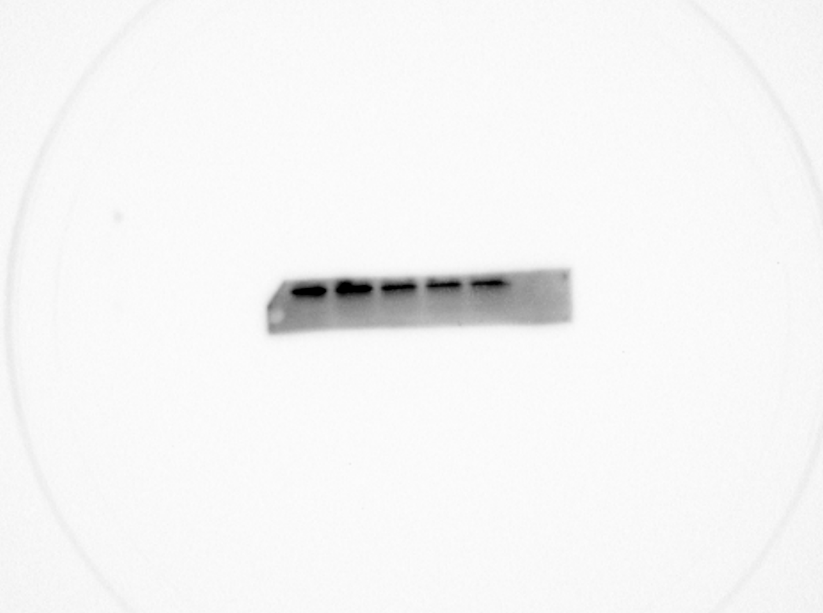

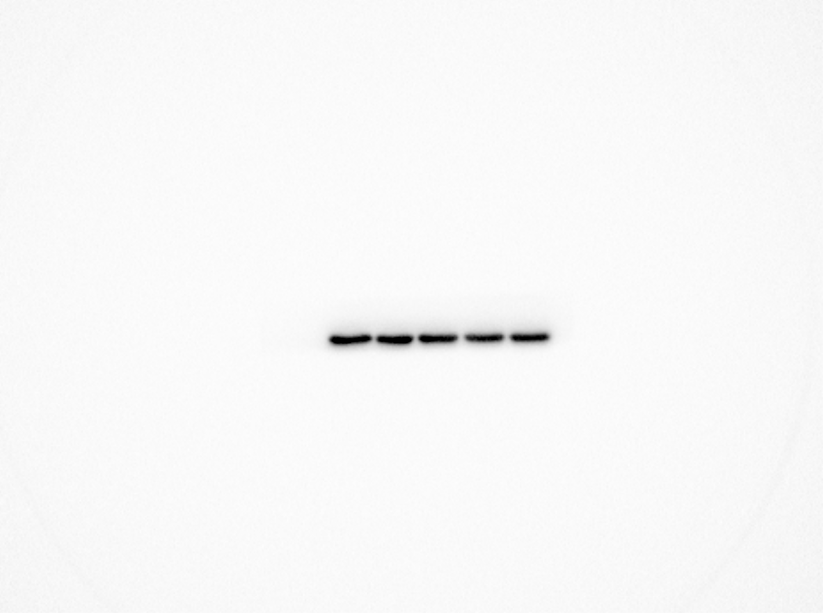


*M. tb+*E6(5μg/ml*)*

*M. tb+*6(10μg/ml)

*M. tb+*E6(1μg/ml)

*M. tb*

Control

*M. tb+*E6(5μg/ml*)*

*M. tb+*6(10μg/ml)

*M. tb+*E6(1μg/ml)

*M. tb*

Control

Cleaved-Caspase3

*β*-Actin

Cleaved-Caspase9

*β*-Actin


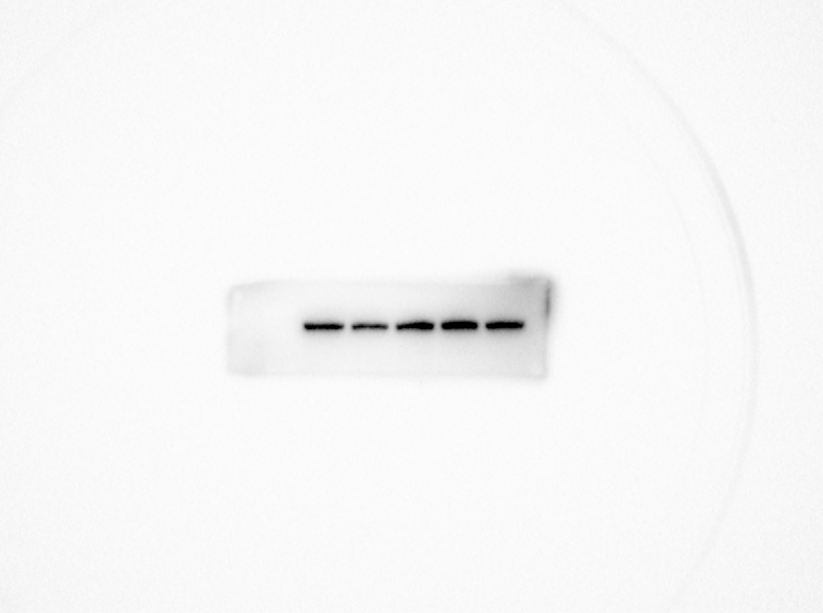

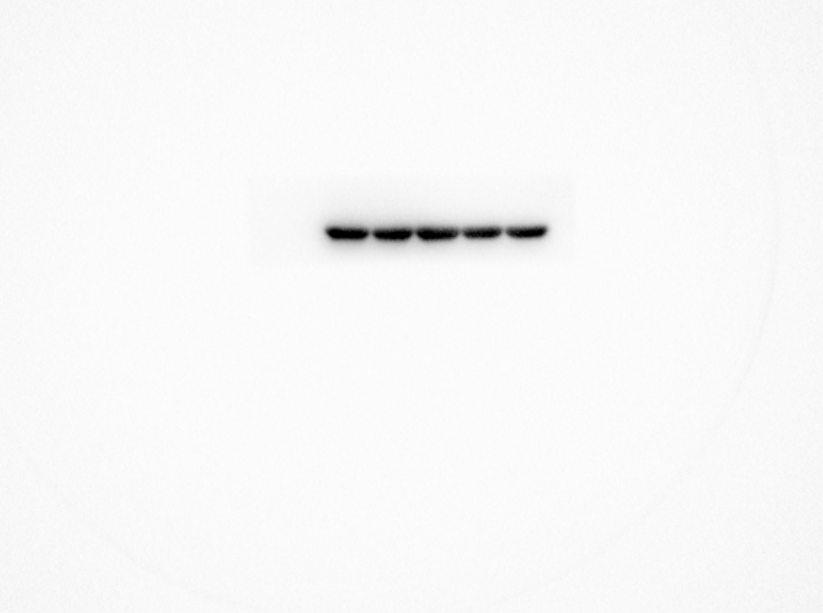
Figure 2F

*M. tb+*E6(5μg/ml*)*

*M. tb+*6(10μg/ml)

*M. tb+*E6(1μg/ml)

*M. tb*

Control

*M. tb+*E6(5μg/ml*)*

*M. tb+*6(10μg/ml)

*M. tb+*E6(1μg/ml)

*M. tb*

Control

*β*-Actin

PARP


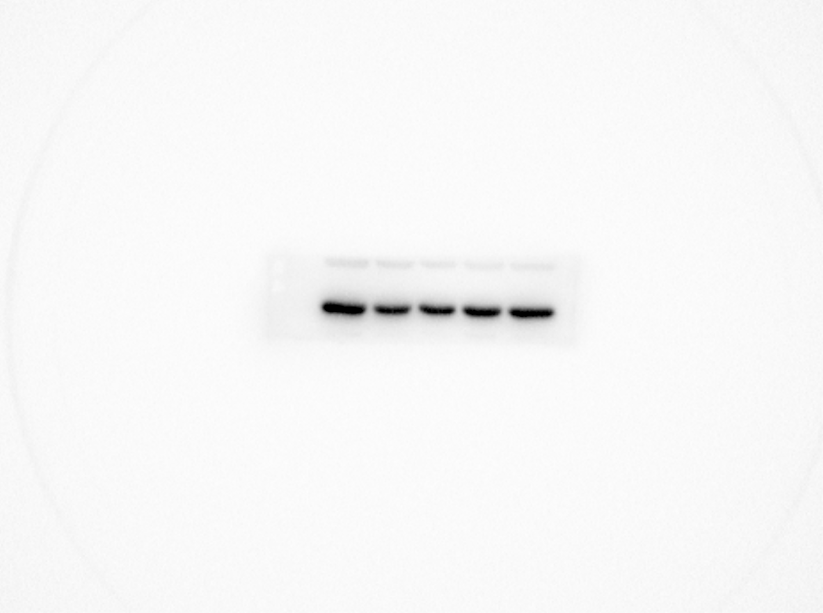

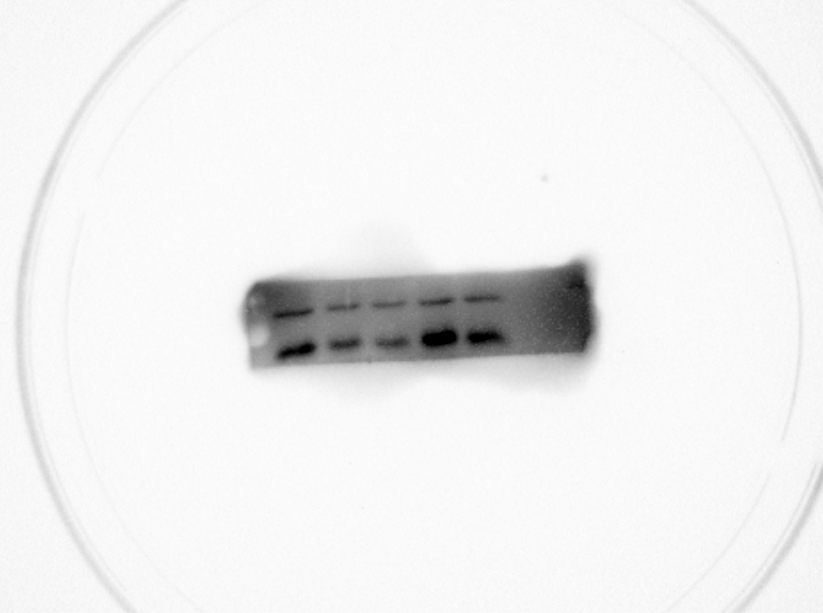


*M. tb+*E6(5μg/ml*)*

*M. tb+*6(10μg/ml)

*M. tb+*E6(1μg/ml)

*M. tb*

Control

*M. tb+*E6(5μg/ml*)*

*M. tb+*6(10μg/ml)

*M. tb+*E6(1μg/ml)

*M. tb*

Control

Cleaved-PARP

*β*-Actin


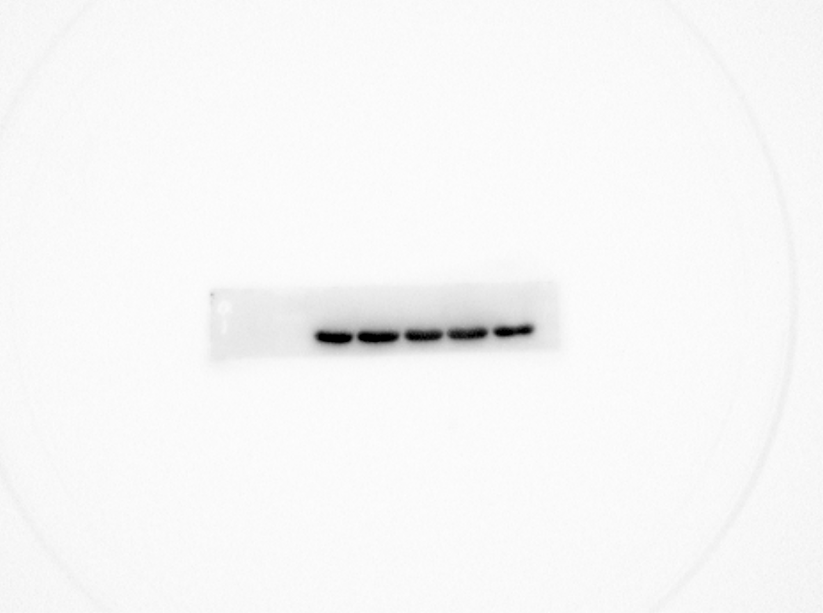

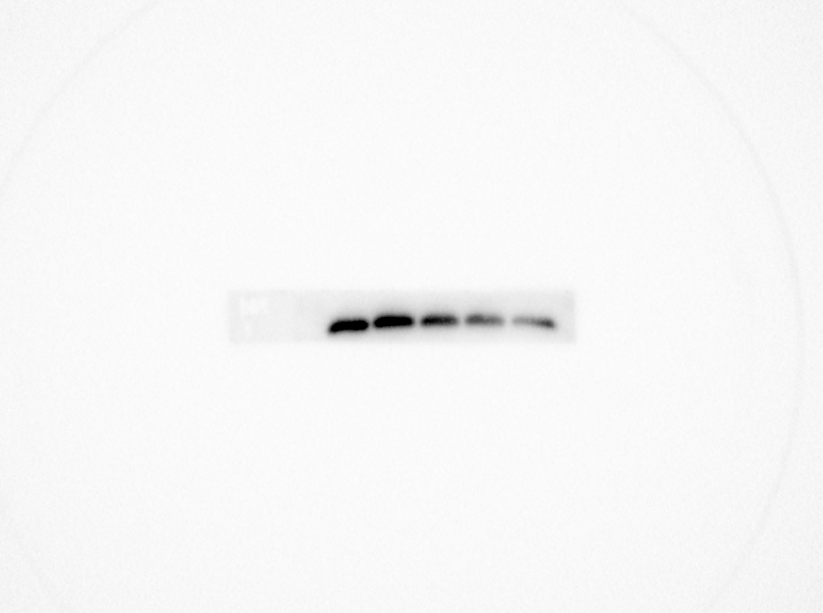


*M. tb+*E6(5μg/ml*)*

*M. tb+*6(10μg/ml)

*M. tb+*E6(1μg/ml)

*M. tb*

Control

*M. tb+*E6(5μg/ml*)*

*M. tb+*6(10μg/ml)

*M. tb+*E6(1μg/ml)

*M. tb*

Control


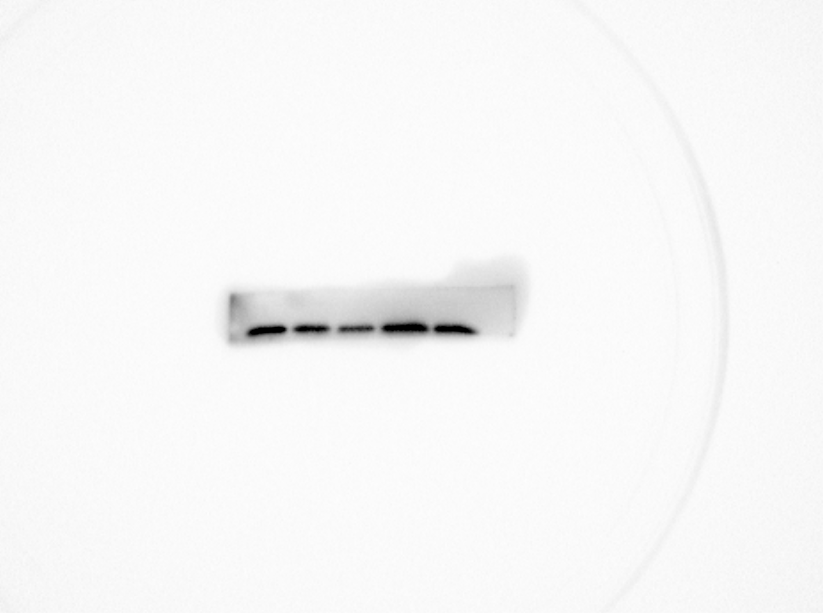

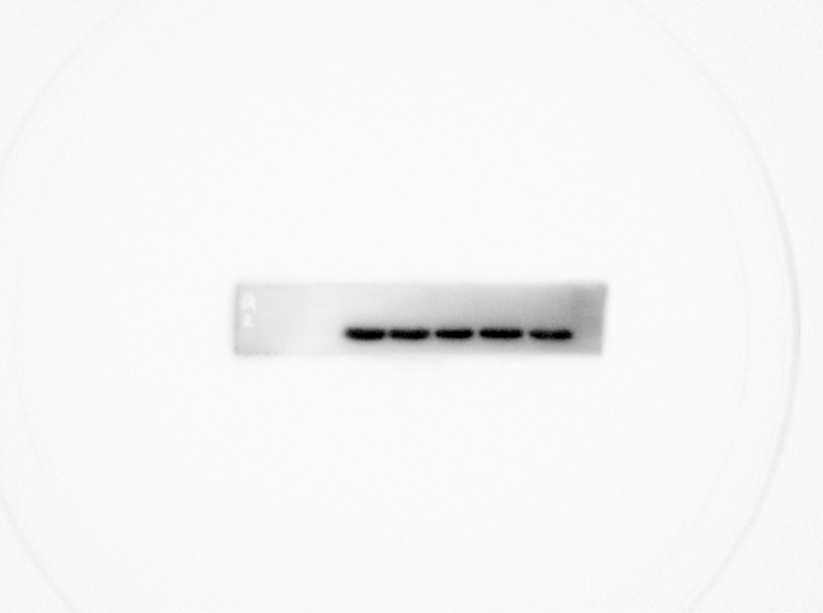


*M. tb+*E6(5μg/ml*)*

*M. tb+*6(10μg/ml)

*M. tb+*E6(1μg/ml)

*M. tb*

Control

*M. tb+*E6(5μg/ml*)*

*M. tb+*6(10μg/ml)

*M. tb+*E6(1μg/ml)

*M. tb*

Control

*β*-Actin

BAX

BCL-2

*β*-Actin

Figure
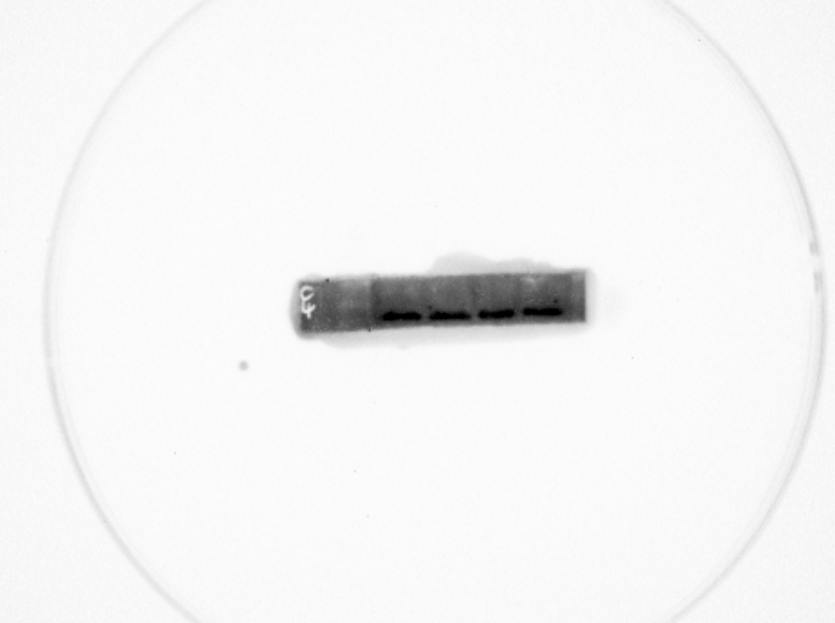
3C


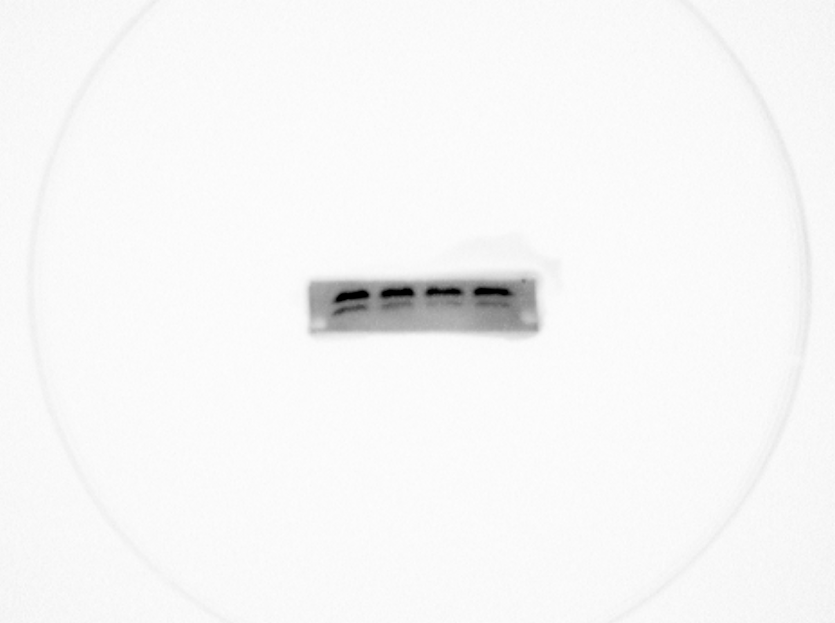


*M. tb+*E6(5μg/ml*)*

AB9100*+M. tb+* E6(5μg/ml*)*

*M. tb*

Control

*M. tb+*E6(5μg/ml*)*

AB9100*+M. tb+* E6(5μg/ml*)*

*M. tb*

Control

*β*-Actin

Caspase3


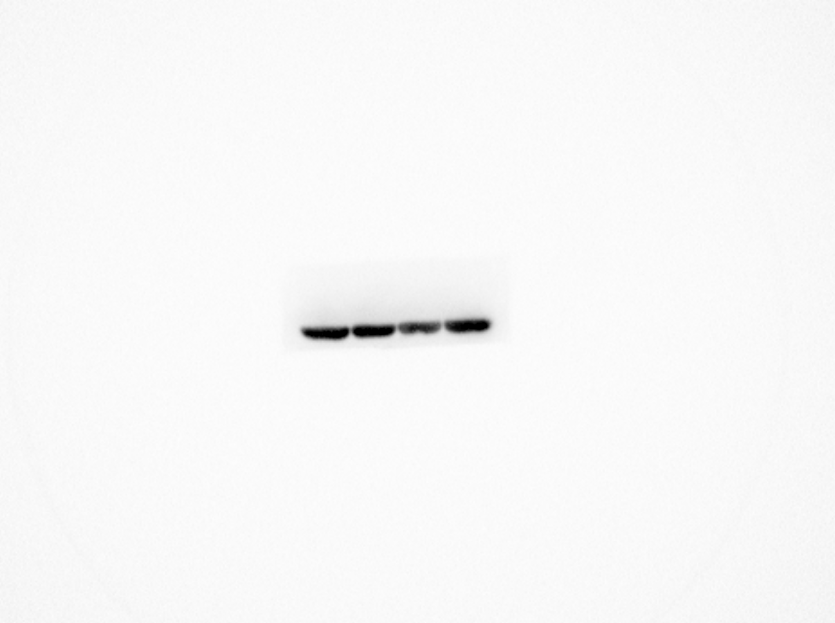

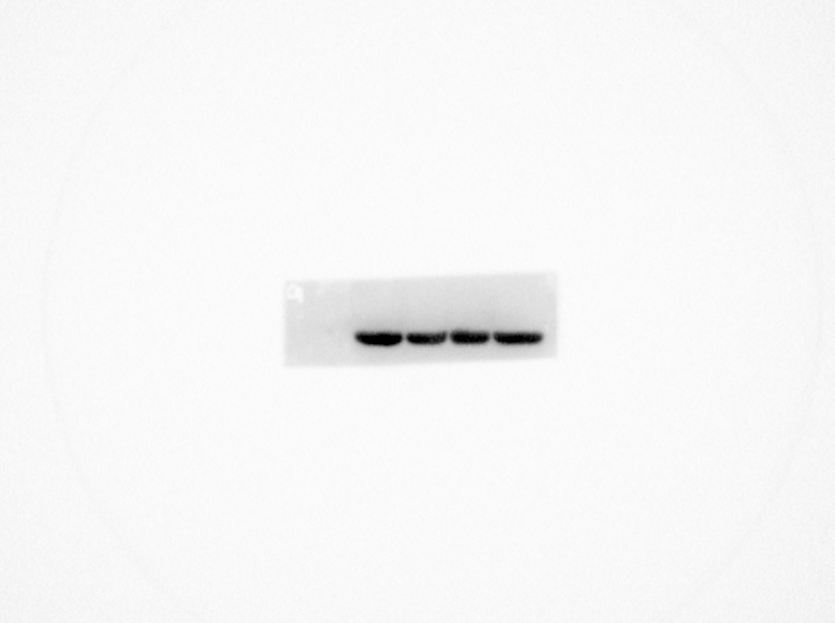


*M. tb+*E6(5μg/ml*)*

AB9100*+M. tb+* E6(5μg/ml*)*

*M. tb*

Control

*M. tb+*E6(5μg/ml*)*

AB9100*+M. tb+* E6(5μg/ml*)*

*M. tb*

Control

*β*-Actin

Caspase9


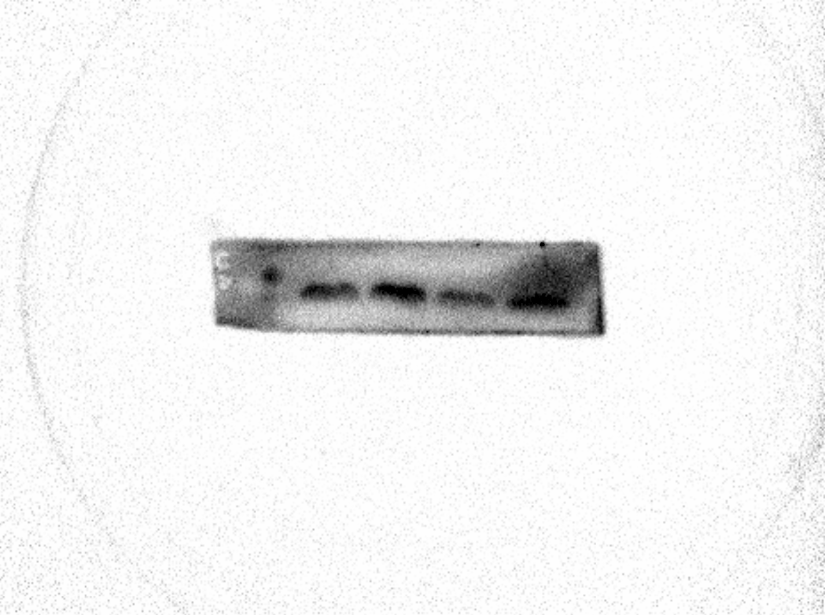

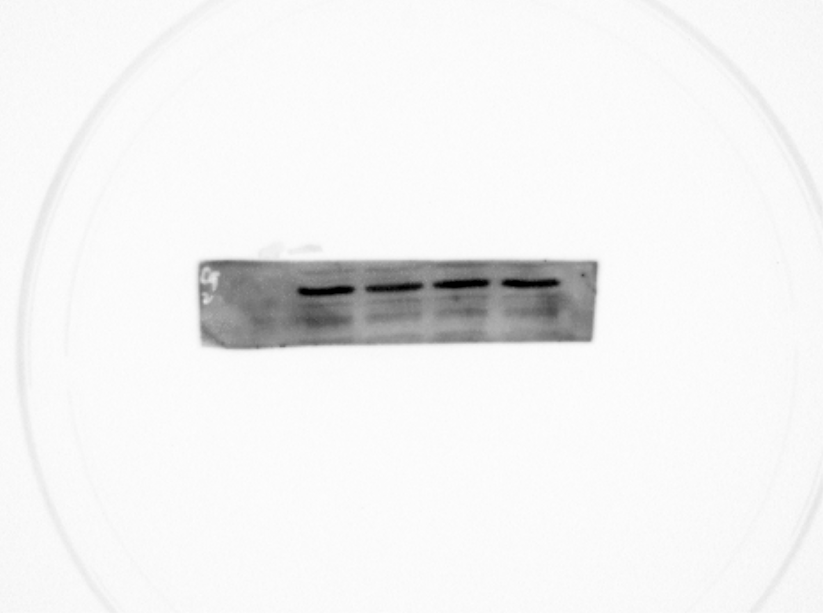

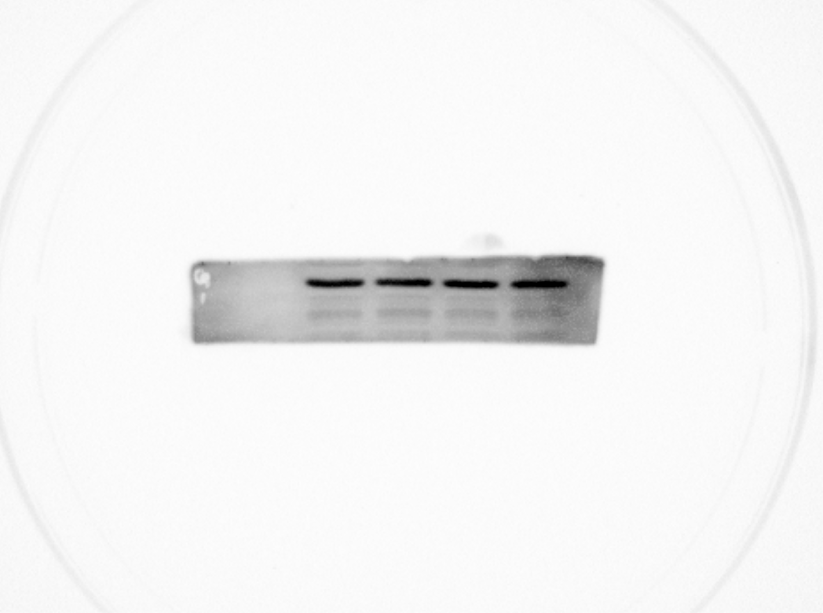

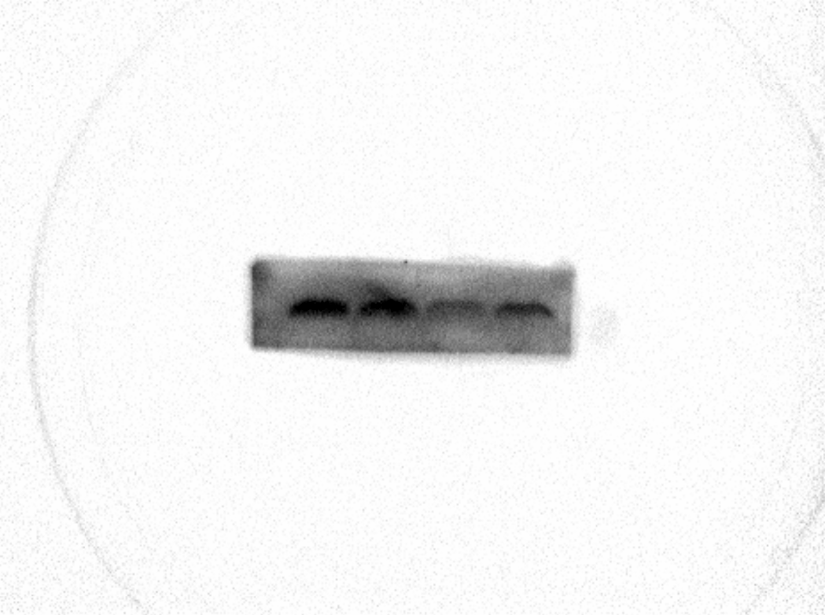


*M. tb+*E6(5μg/ml*)*

AB9100*+M. tb+* E6(5μg/ml*)*

*M. tb*

Control

*M. tb+*E6(5μg/ml*)*

AB9100*+M. tb+* E6(5μg/ml*)*

*M. tb*

Control

*M. tb+*E6(5μg/ml*)*

AB9100*+M. tb+* E6(5μg/ml*)*

*M. tb*

Control

*M. tb+*E6(5μg/ml*)*

AB9100*+M. tb+* E6(5μg/ml*)*

*M. tb*

Control

*β*-Actin

Cleaved-Caspase3


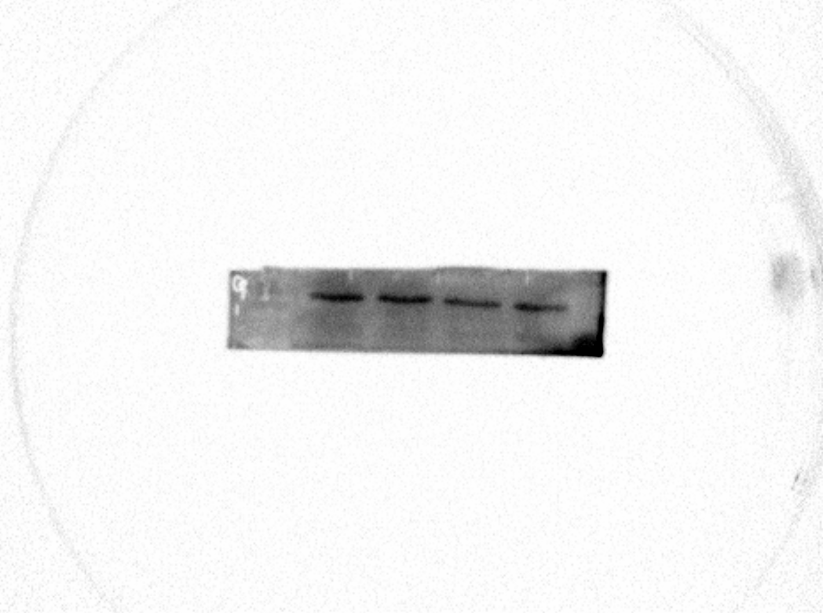

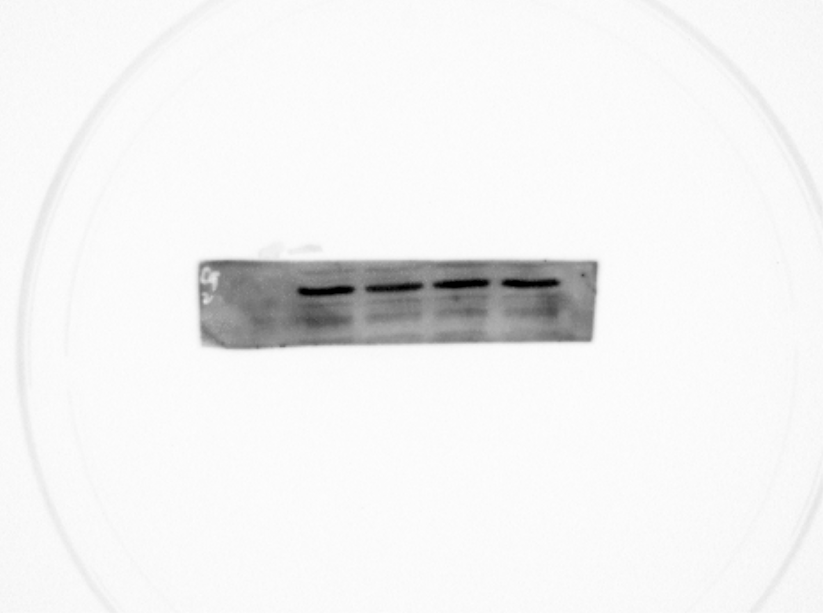


*M. tb+*E6(5μg/ml*)*

AB9100*+M. tb+* E6(5μg/ml*)*

*M. tb*

Control

*M. tb+*E6(5μg/ml*)*

AB9100*+M. tb+* E6(5μg/ml*)*

*M. tb*

Control

Cleaved-Caspase9

*β*-Actin

Figure 4


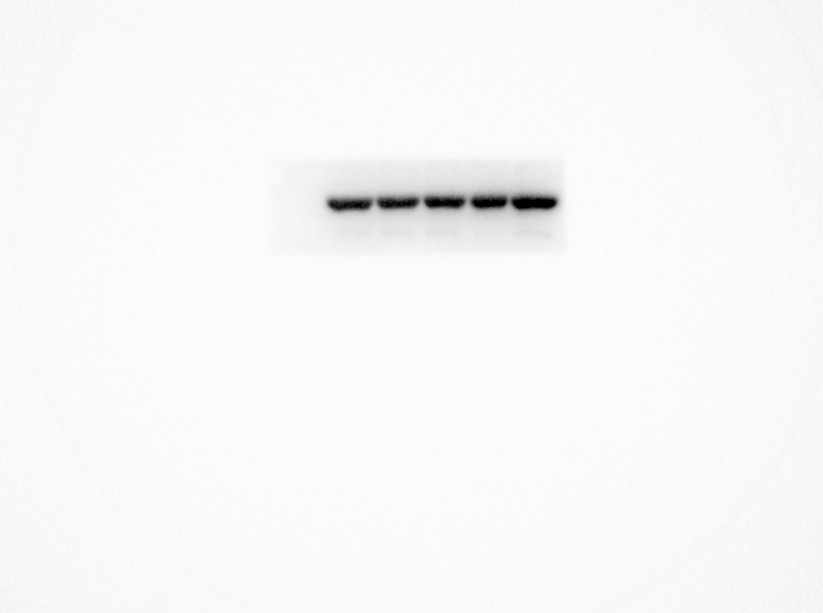

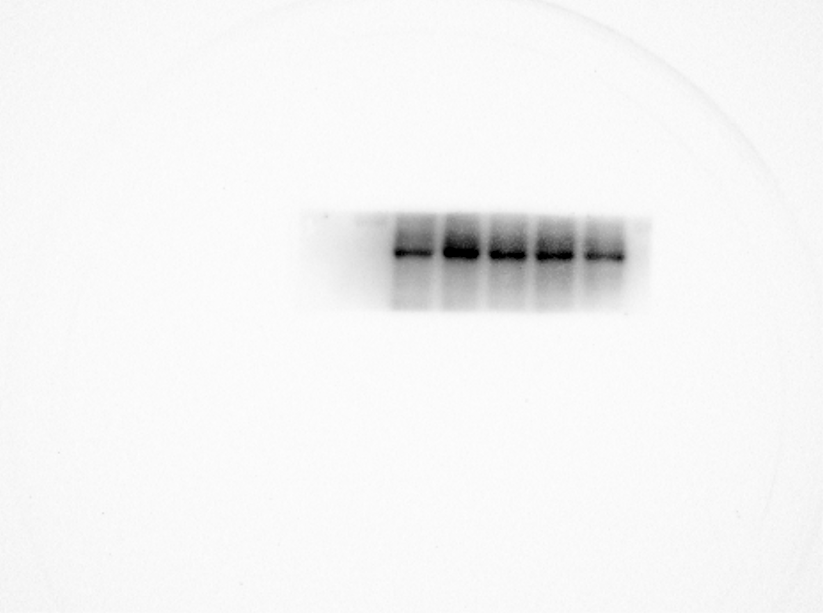


*M. tb+*E6(5μg/ml*)*

*M. tb+*6(10μg/ml)

*M. tb+*E6(1μg/ml)

*M. tb*

Control

*M. tb+*E6(5μg/ml*)*

*M. tb+*6(10μg/ml)

*M. tb+*E6(1μg/ml)

*M. tb*

Control

TLR2

*β*-Actin
