# Supplementary figures and images for: Early secretory antigen target of 6-kDa of Mycobacterium tuberculosis inhibits macrophage apoptosis and host defense via TLR2
Source: Respir Res. 2025 Apr 9;26:131. doi: 10.1186/s12931-025-03210-z (PMC11983766; doi:10.1186/s12931-025-03210-z)

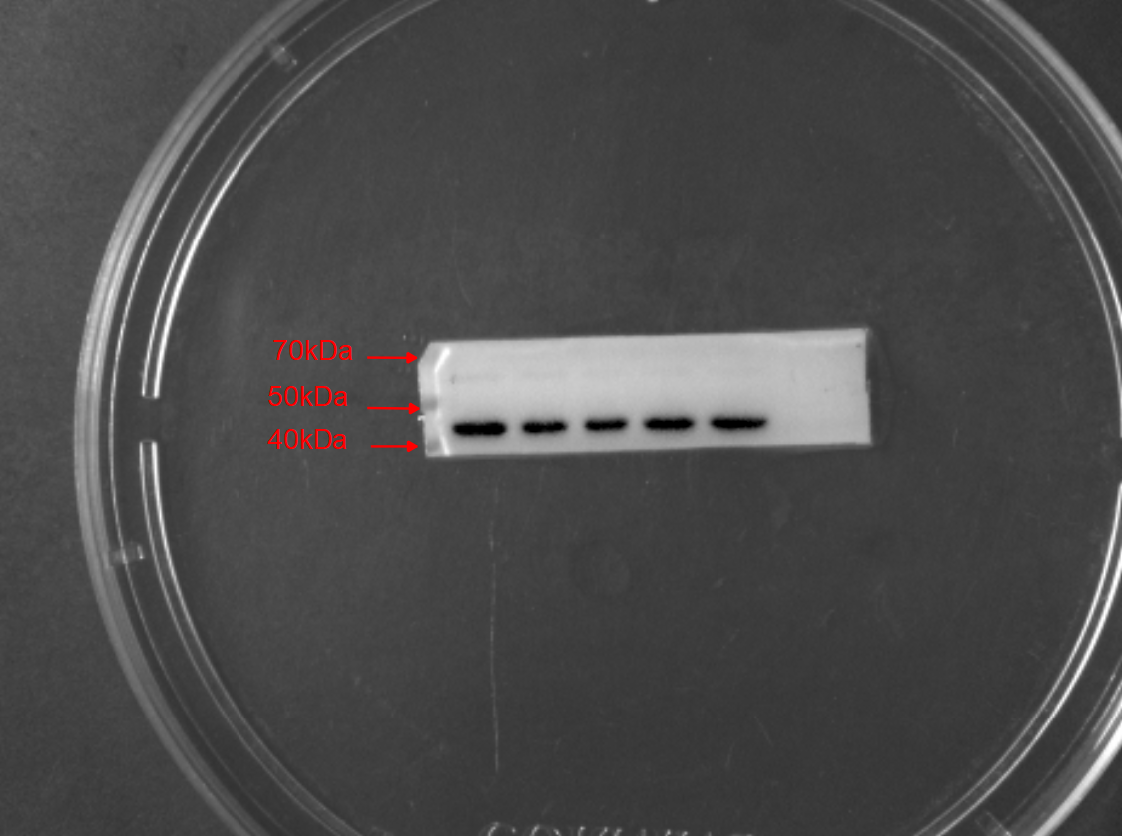

Supplement: Supplementary file 2 — Additional file 2. [file 12931_2025_3210_MOESM2_ESM.zip › WB RAW DATA - ╕▒▒╛/Figure2E/CASP3/lenovo 2021-10-07-beta-actin-1+lenovo 2021-10-07-beta-actin-1-.tif]

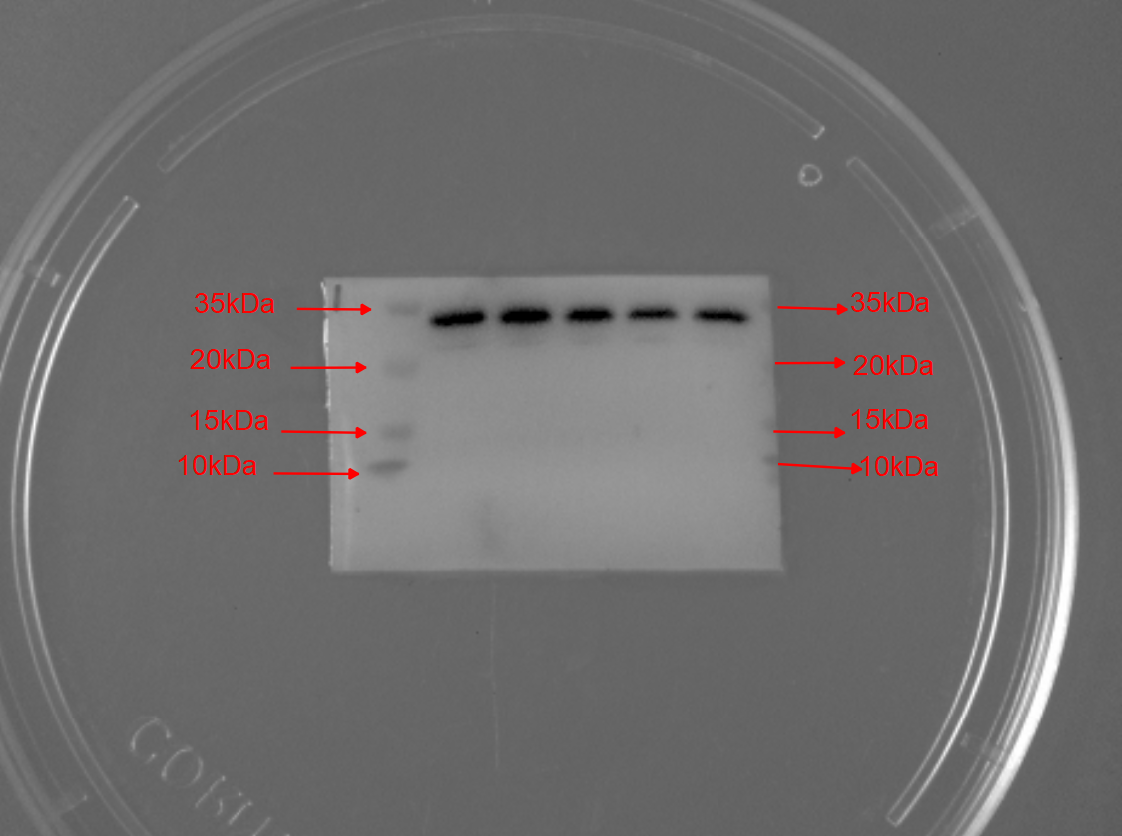

Supplement: Supplementary file 2 — Additional file 2. [file 12931_2025_3210_MOESM2_ESM.zip › WB RAW DATA - ╕▒▒╛/Figure2E/CASP3/lenovo 2021-10-07-caspase3-1+lenovo 2021-10-07_13h41m48s.tif]

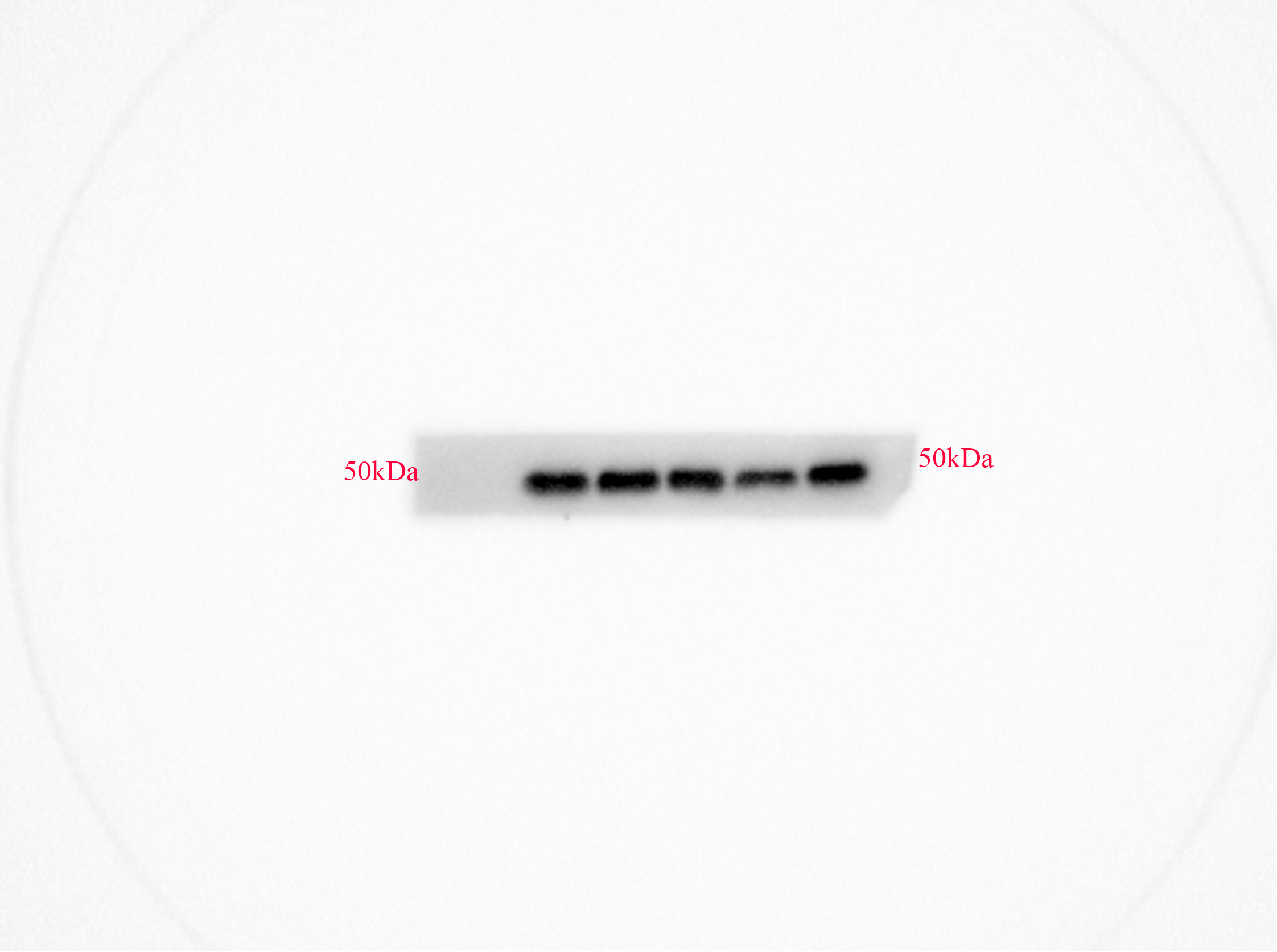

Supplement: Supplementary file 2 — Additional file 2. [file 12931_2025_3210_MOESM2_ESM.zip › WB RAW DATA - ╕▒▒╛/Figure2E/CASP9/lenovo 2022-04-26-Caspase9-6.tif]

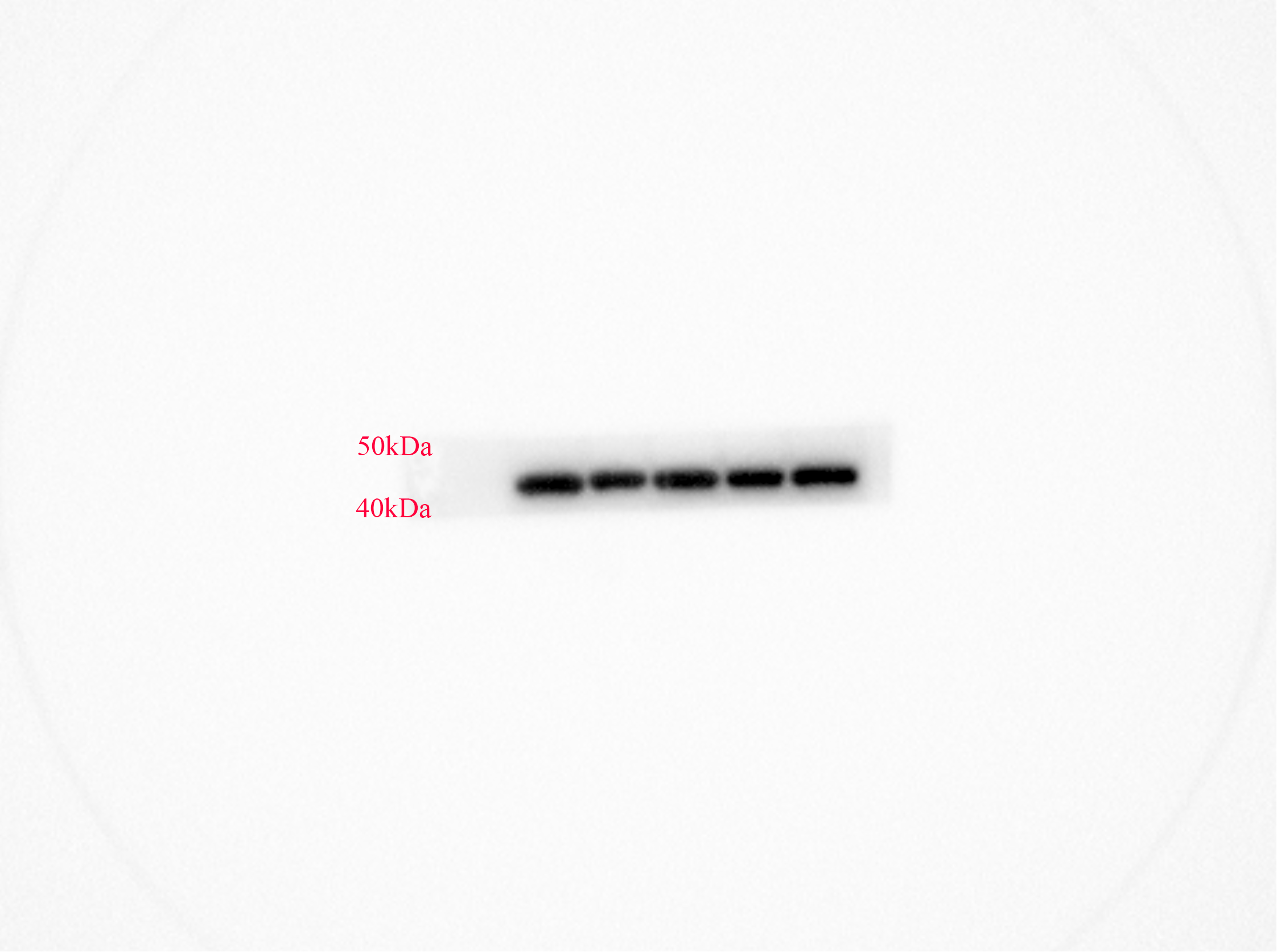

Supplement: Supplementary file 2 — Additional file 2. [file 12931_2025_3210_MOESM2_ESM.zip › WB RAW DATA - ╕▒▒╛/Figure2E/CASP9/lenovo 2022-04-27-beta-actin-6.tif]

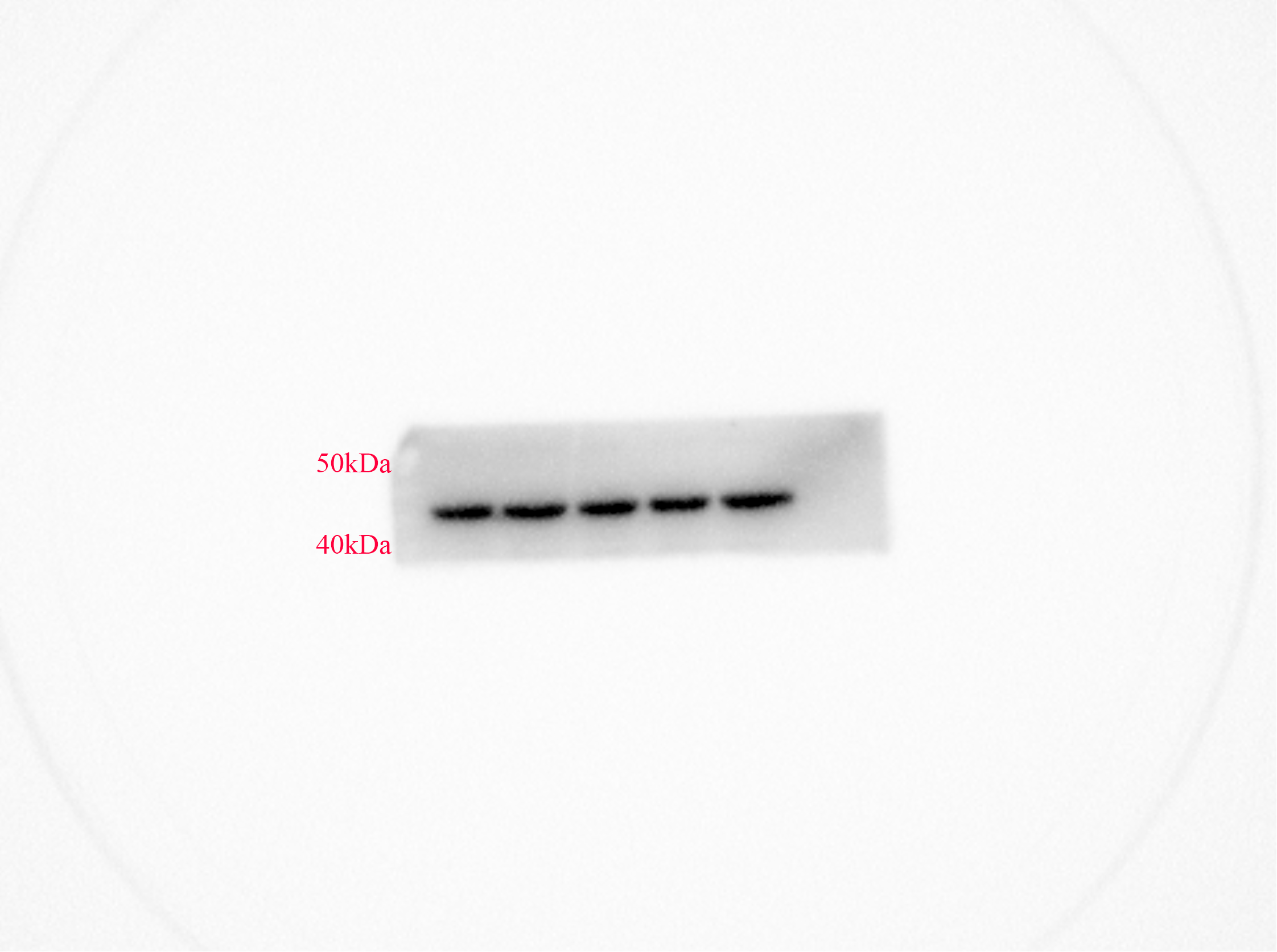

Supplement: Supplementary file 2 — Additional file 2. [file 12931_2025_3210_MOESM2_ESM.zip › WB RAW DATA - ╕▒▒╛/Figure2E/Cleaved-CASP3/lenovo 2022-03-13-beta-actin-1.tif]

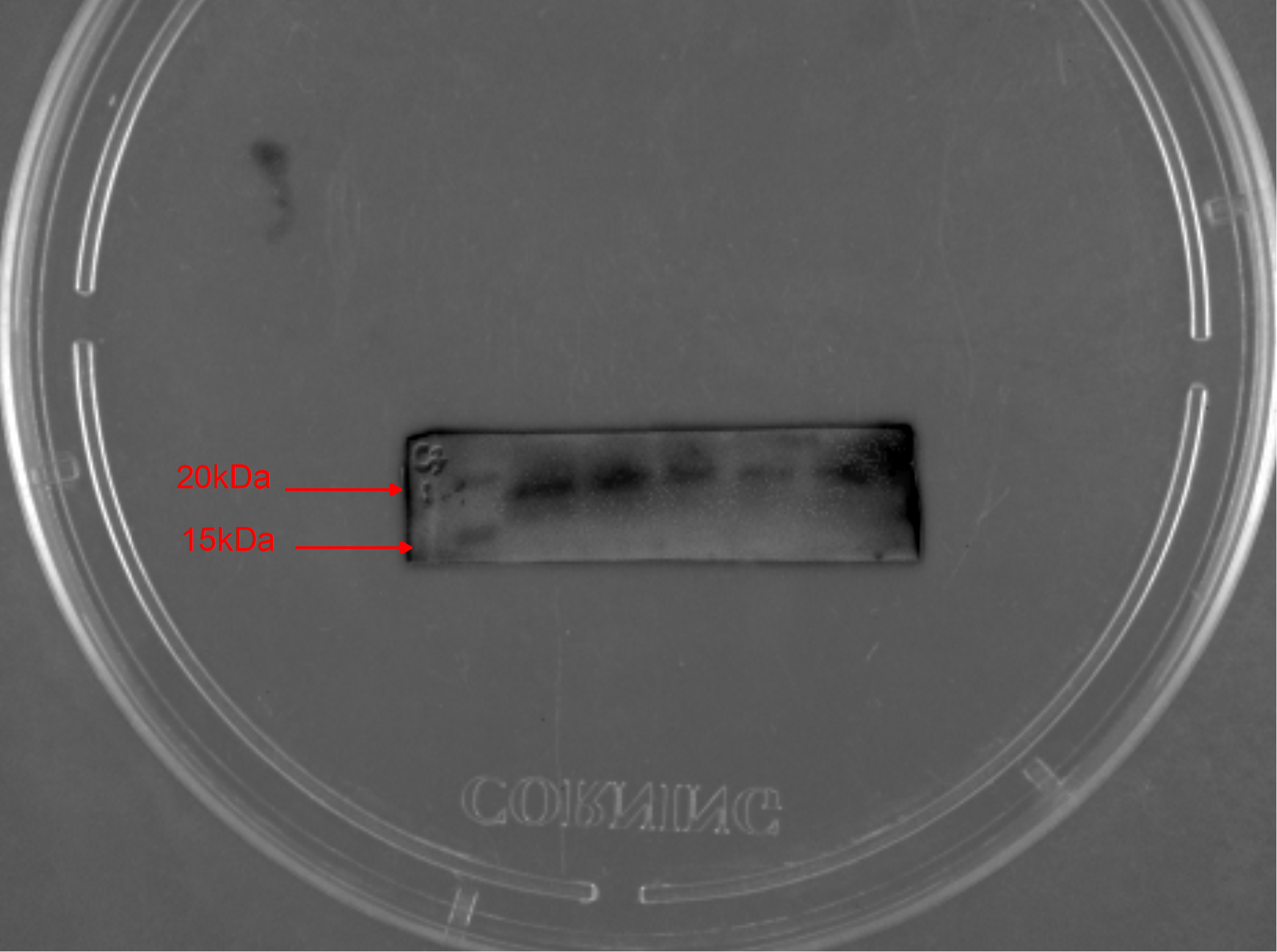

Supplement: Supplementary file 2 — Additional file 2. [file 12931_2025_3210_MOESM2_ESM.zip › WB RAW DATA - ╕▒▒╛/Figure2E/Cleaved-CASP3/lenovo 2022-03-13-Cleaved-caspase3-1+lenovo 2022-03-13-Cleaved-caspase3-1-.tif]

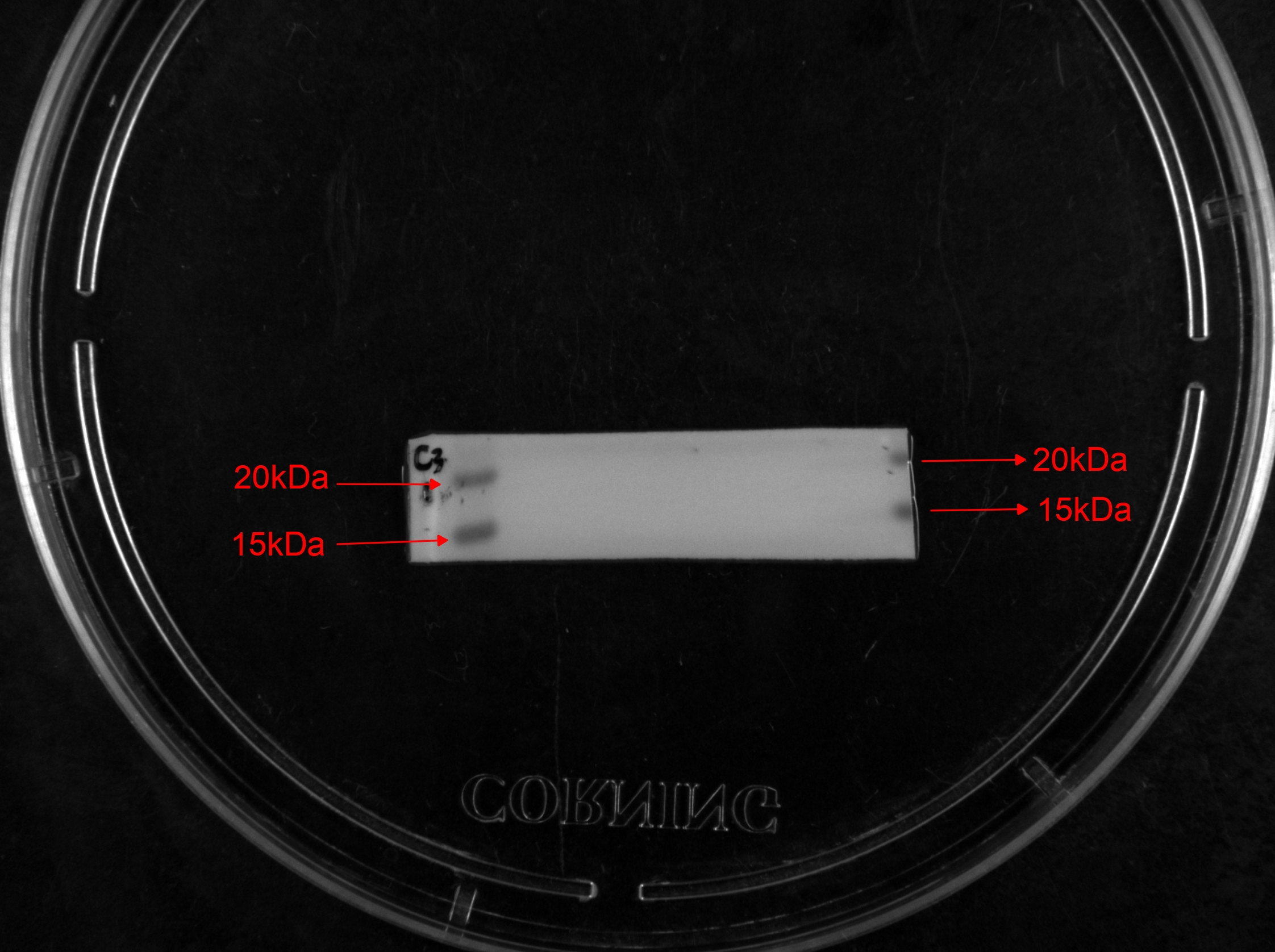

Supplement: Supplementary file 2 — Additional file 2. [file 12931_2025_3210_MOESM2_ESM.zip › WB RAW DATA - ╕▒▒╛/Figure2E/Cleaved-CASP3/lenovo 2022-03-13-Cleaved-caspase3-1-.tif]

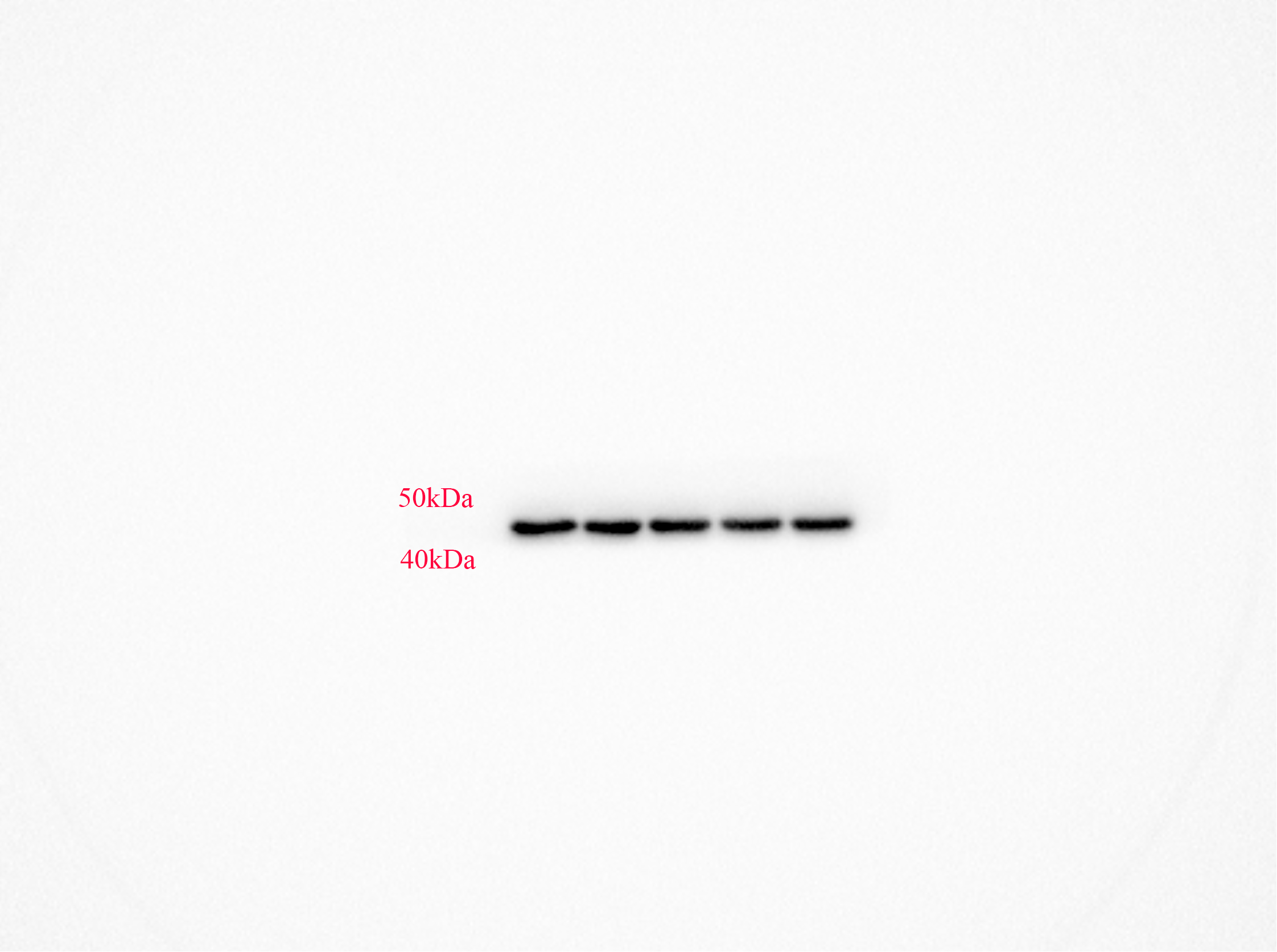

Supplement: Supplementary file 2 — Additional file 2. [file 12931_2025_3210_MOESM2_ESM.zip › WB RAW DATA - ╕▒▒╛/Figure2E/Cleaved-CASP9/lenovo 2022-03-09-beta-actin-4.tif]

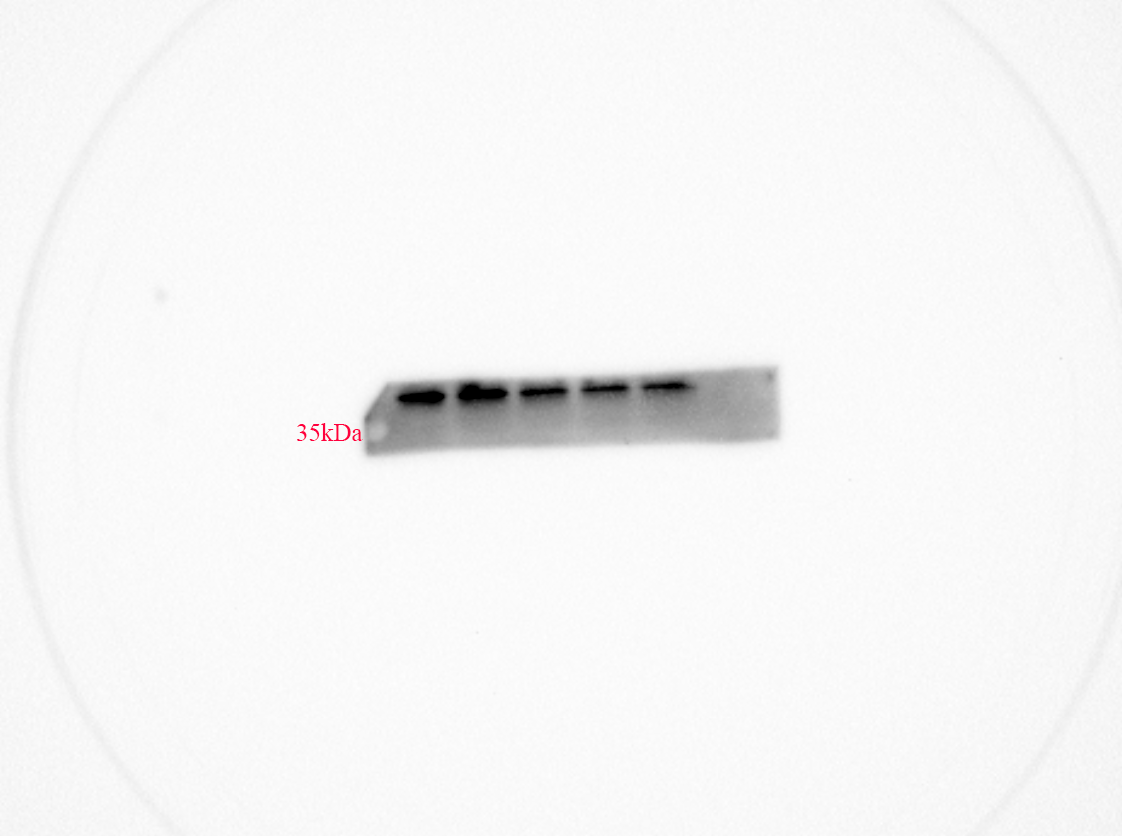

Supplement: Supplementary file 2 — Additional file 2. [file 12931_2025_3210_MOESM2_ESM.zip › WB RAW DATA - ╕▒▒╛/Figure2E/Cleaved-CASP9/lenovo 2022-03-09-Cleaved-Caspase9-4-1.tif]

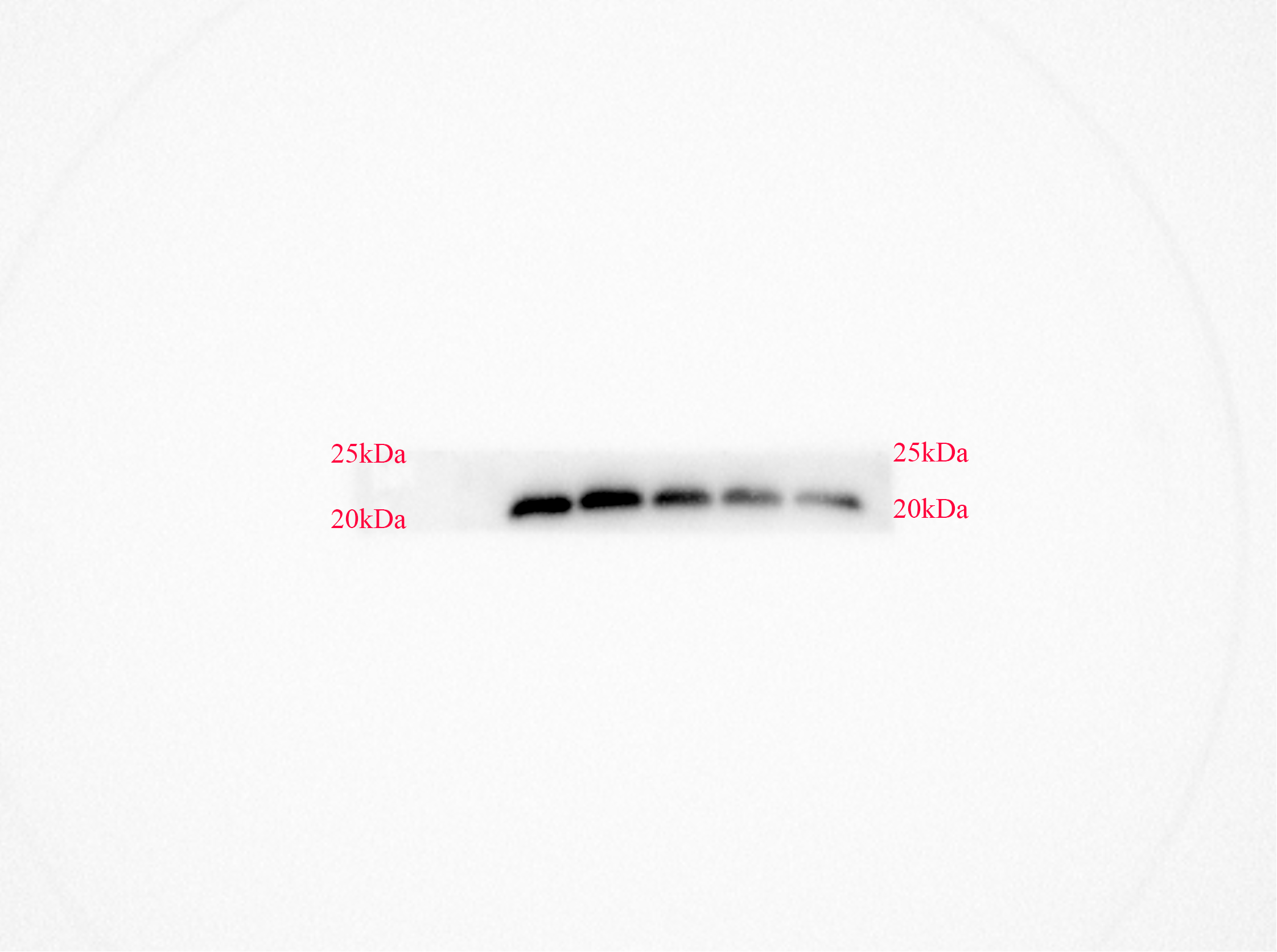

Supplement: Supplementary file 2 — Additional file 2. [file 12931_2025_3210_MOESM2_ESM.zip › WB RAW DATA - ╕▒▒╛/Figure2F/BAX/lenovo 2022-03-29_bax-1-3.tif]

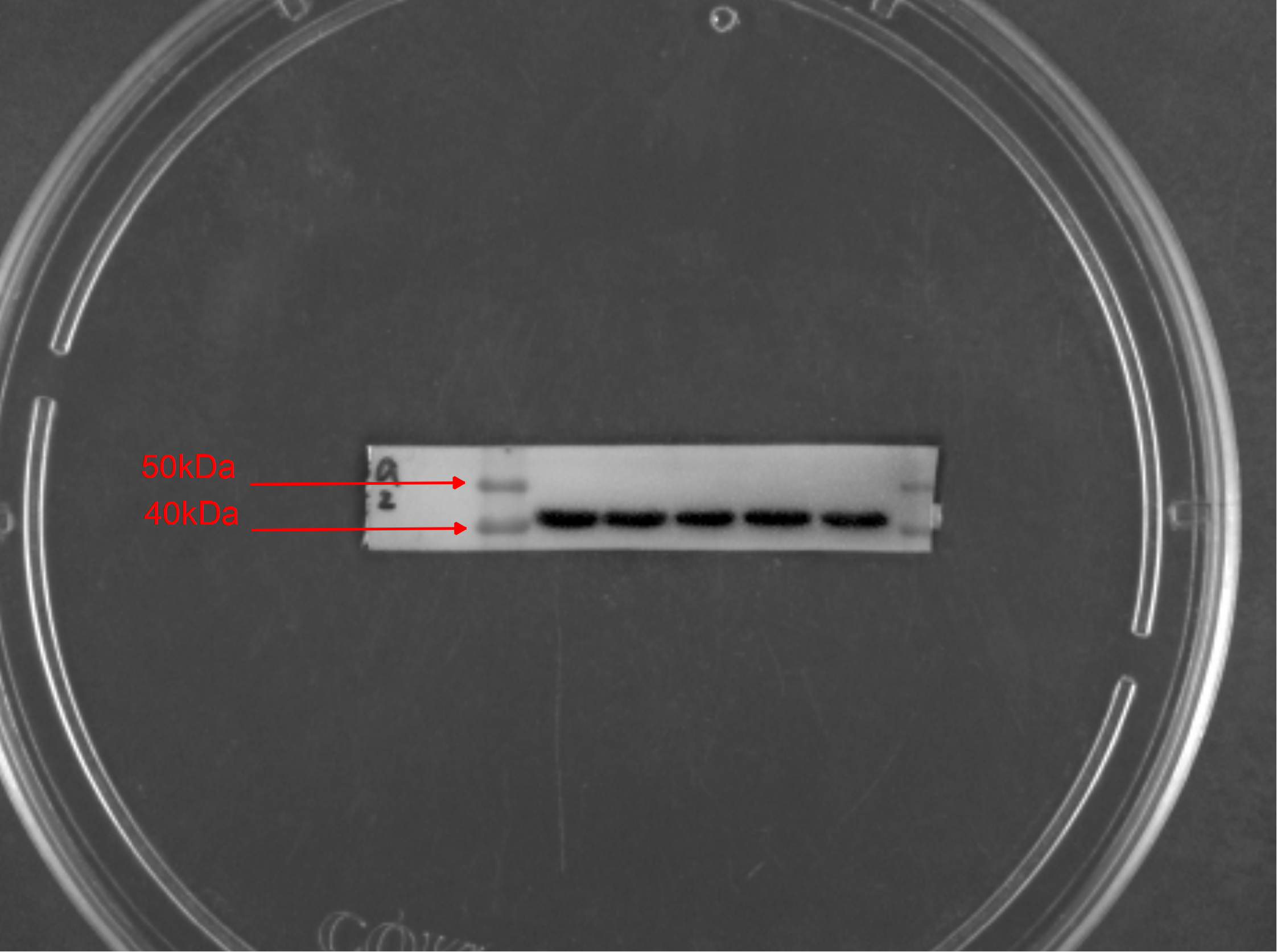

Supplement: Supplementary file 2 — Additional file 2. [file 12931_2025_3210_MOESM2_ESM.zip › WB RAW DATA - ╕▒▒╛/Figure2F/BAX/lenovo 2022-03-29_beta-actin-1-3-+lenovo 2022-03-29_beta-actin-1-3.tif]

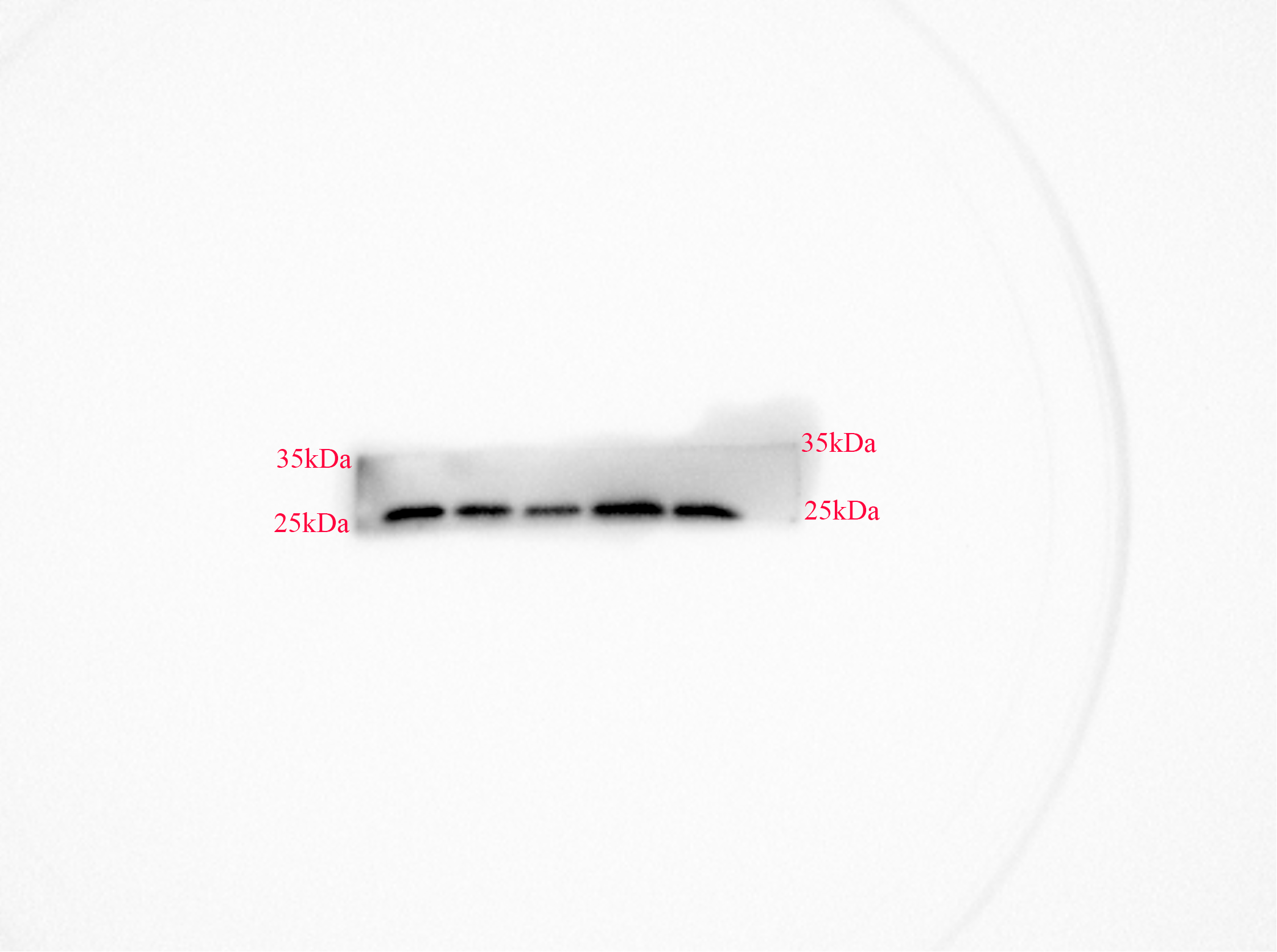

Supplement: Supplementary file 2 — Additional file 2. [file 12931_2025_3210_MOESM2_ESM.zip › WB RAW DATA - ╕▒▒╛/Figure2F/BCL2/lenovo 2022-03-29_bcl-2-2-2.tif]

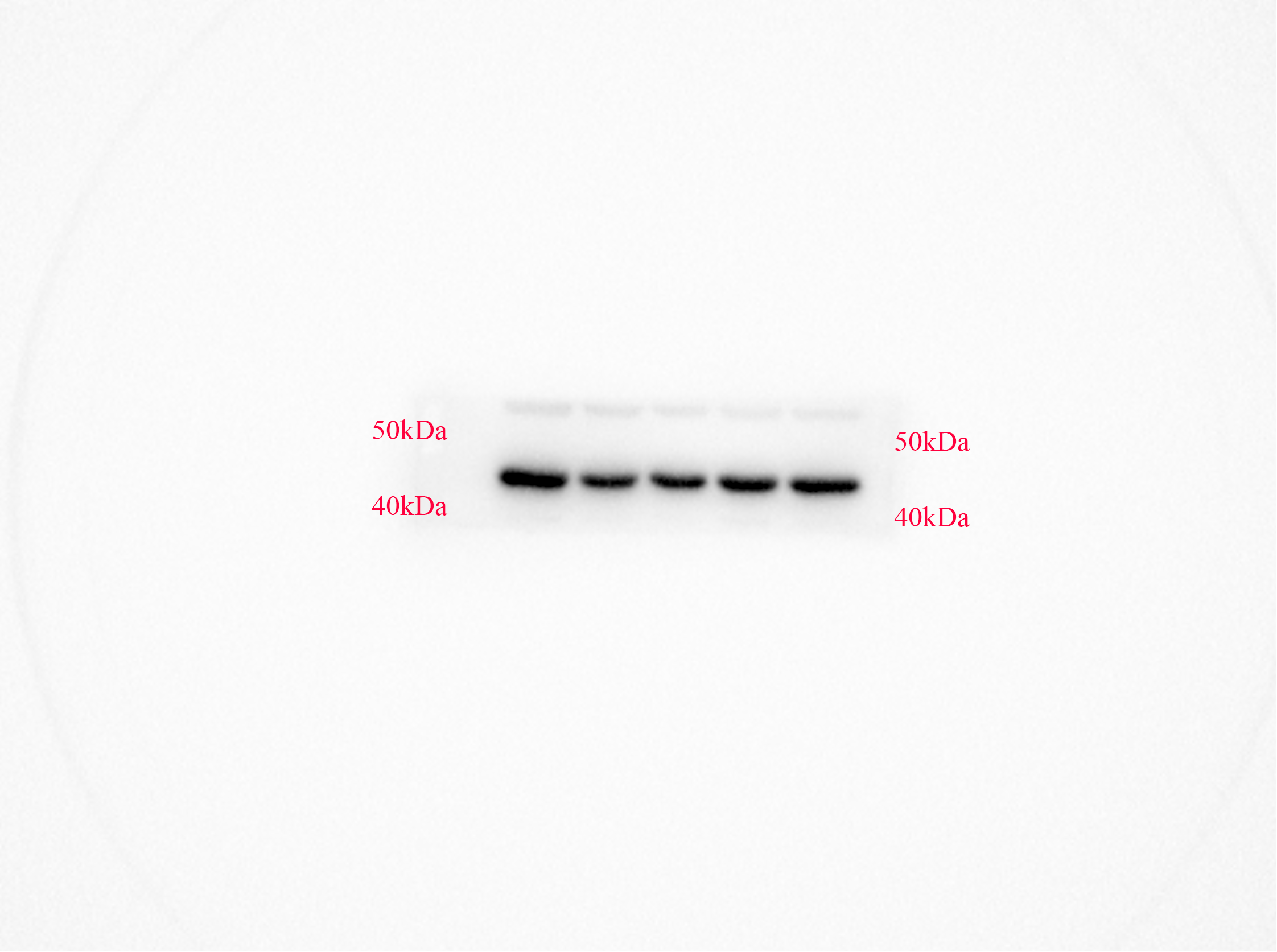

Supplement: Supplementary file 2 — Additional file 2. [file 12931_2025_3210_MOESM2_ESM.zip › WB RAW DATA - ╕▒▒╛/Figure2F/Cleaved-PARP/lenovo 2021-12-25-beta-actin-2.tif]

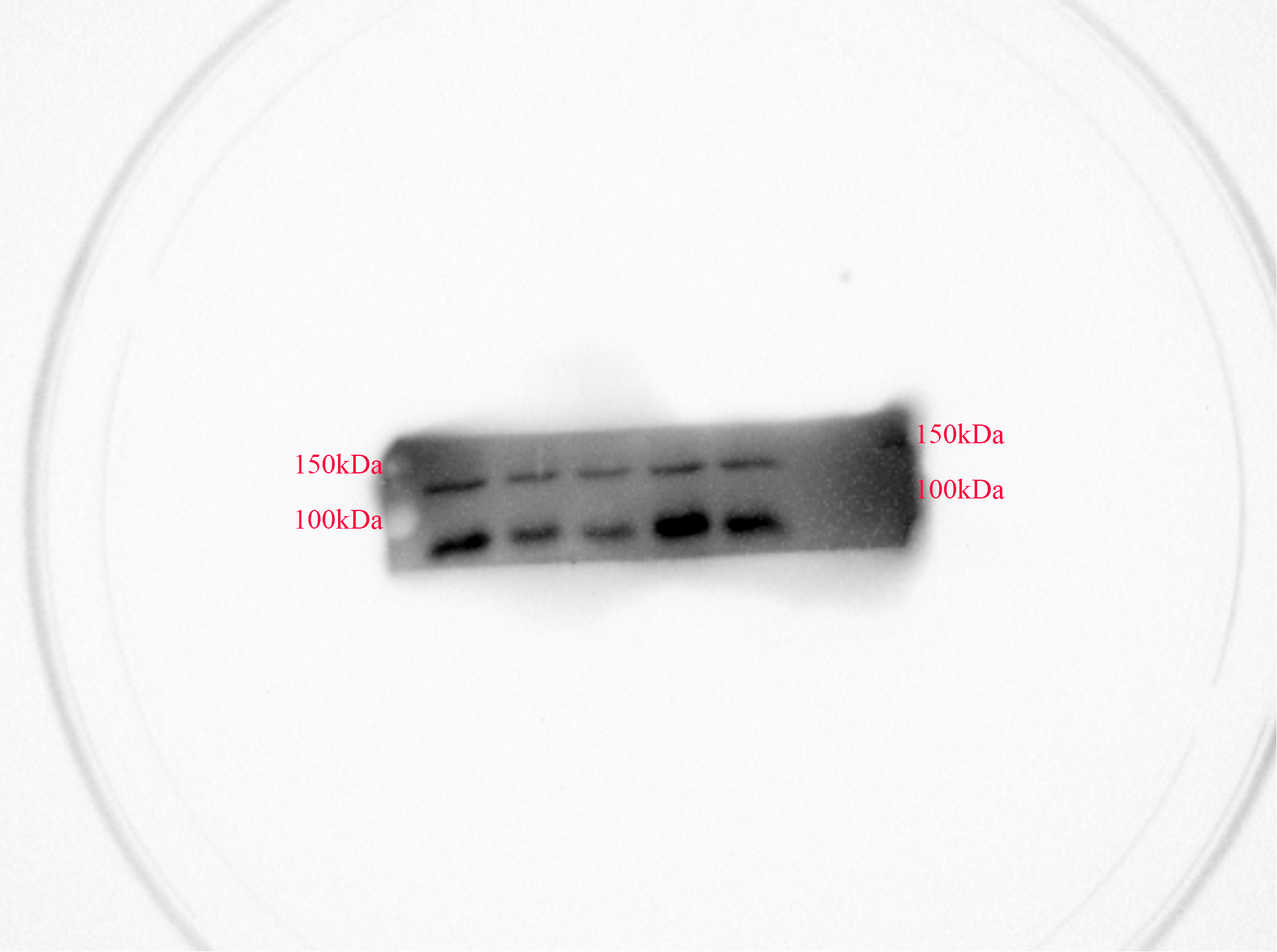

Supplement: Supplementary file 2 — Additional file 2. [file 12931_2025_3210_MOESM2_ESM.zip › WB RAW DATA - ╕▒▒╛/Figure2F/Cleaved-PARP/lenovo 2021-12-25-Cleaved-PARP-2.tif]

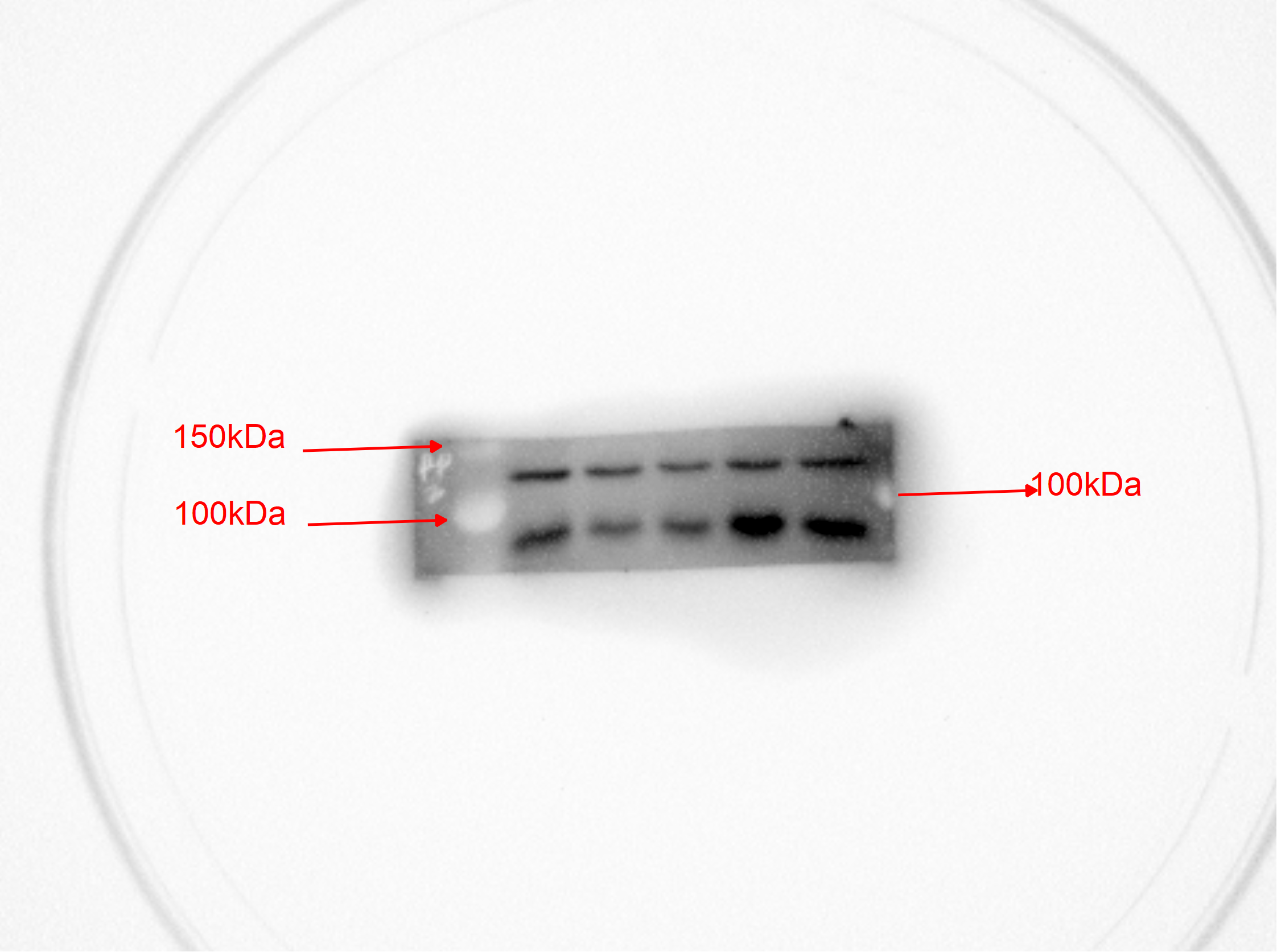

Supplement: Supplementary file 2 — Additional file 2. [file 12931_2025_3210_MOESM2_ESM.zip › WB RAW DATA - ╕▒▒╛/Figure2F/Cleaved-PARP/lenovo 2021-12-25-Cleaved-PARP-3-2.tif]

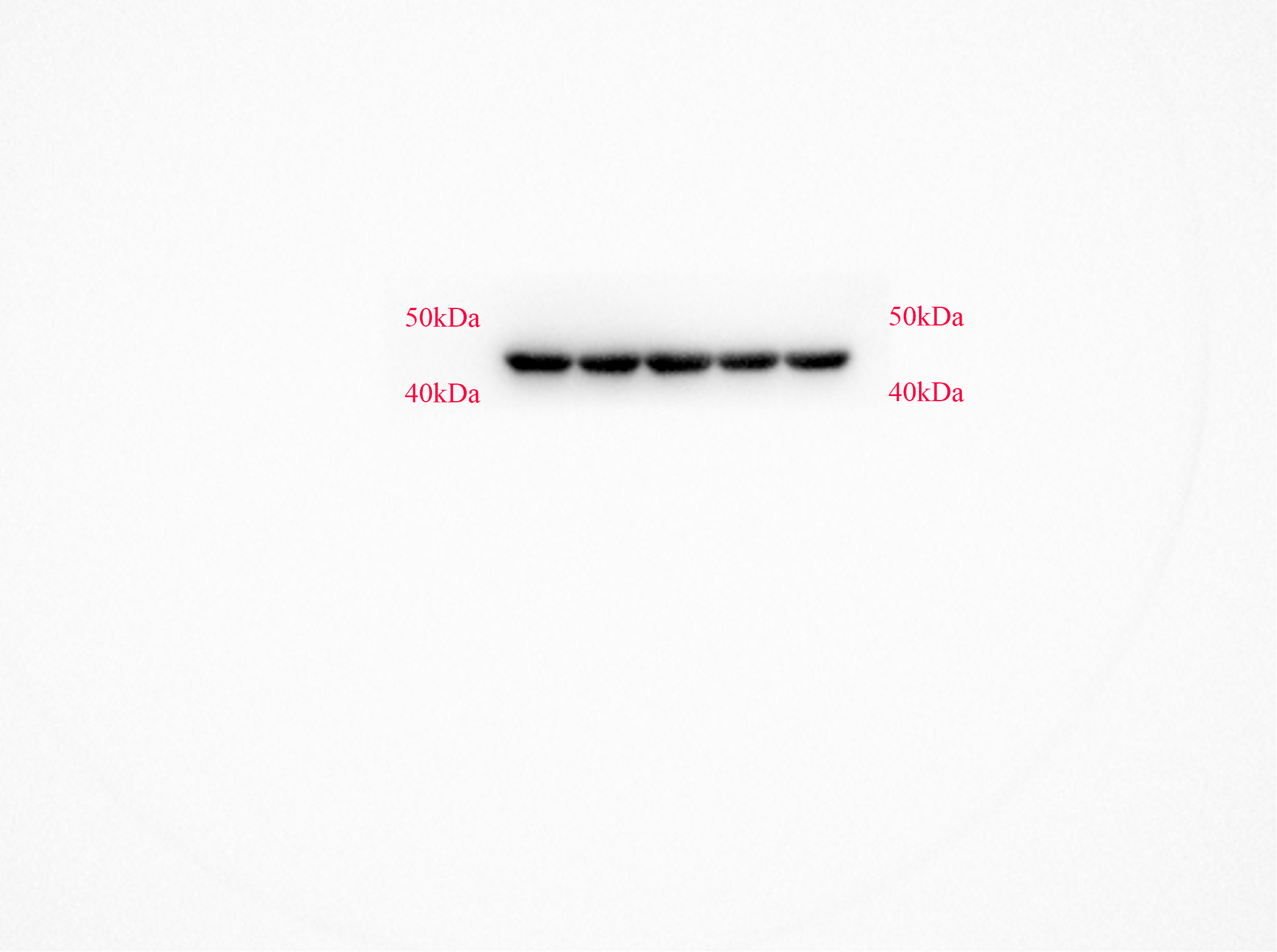

Supplement: Supplementary file 2 — Additional file 2. [file 12931_2025_3210_MOESM2_ESM.zip › WB RAW DATA - ╕▒▒╛/Figure2F/PARP/lenovo 2021-12-05-beta-actin-1.tif]

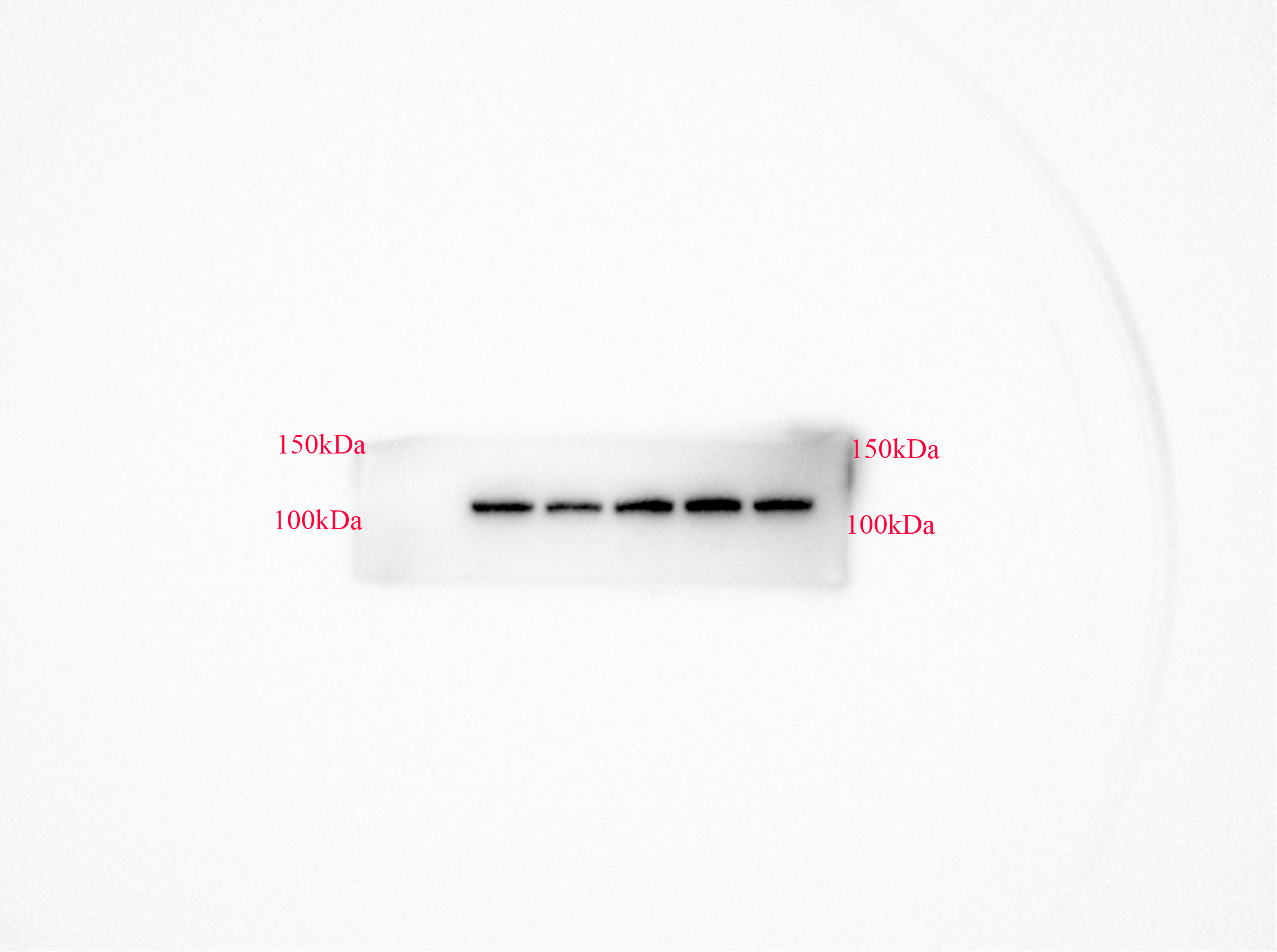

Supplement: Supplementary file 2 — Additional file 2. [file 12931_2025_3210_MOESM2_ESM.zip › WB RAW DATA - ╕▒▒╛/Figure2F/PARP/lenovo 2021-12-05-PARP-1.tif]

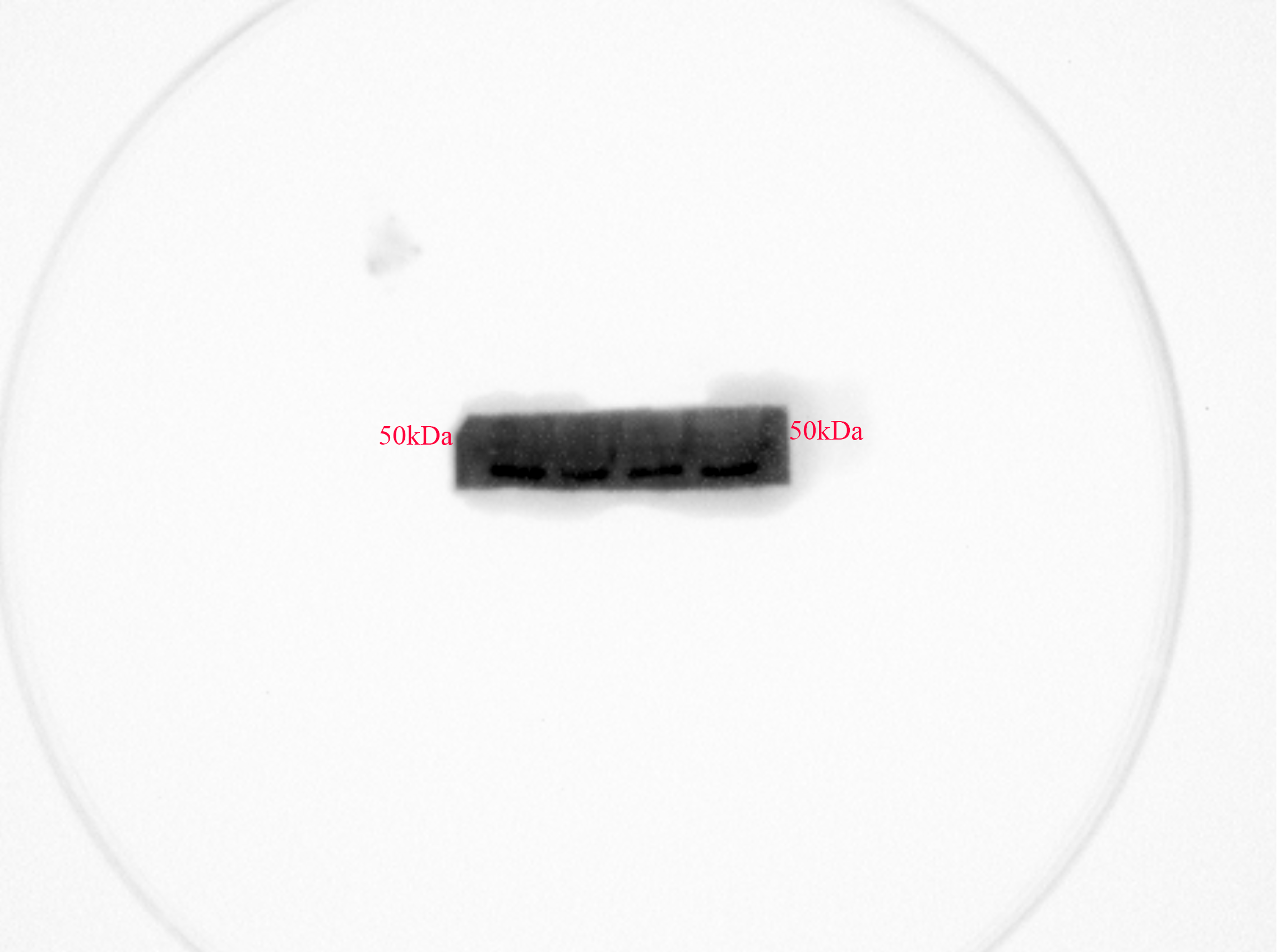

Supplement: Supplementary file 2 — Additional file 2. [file 12931_2025_3210_MOESM2_ESM.zip › WB RAW DATA - ╕▒▒╛/Figure3C/CASP3/lenovo 2022-10-05-beta-actin-9.tif]

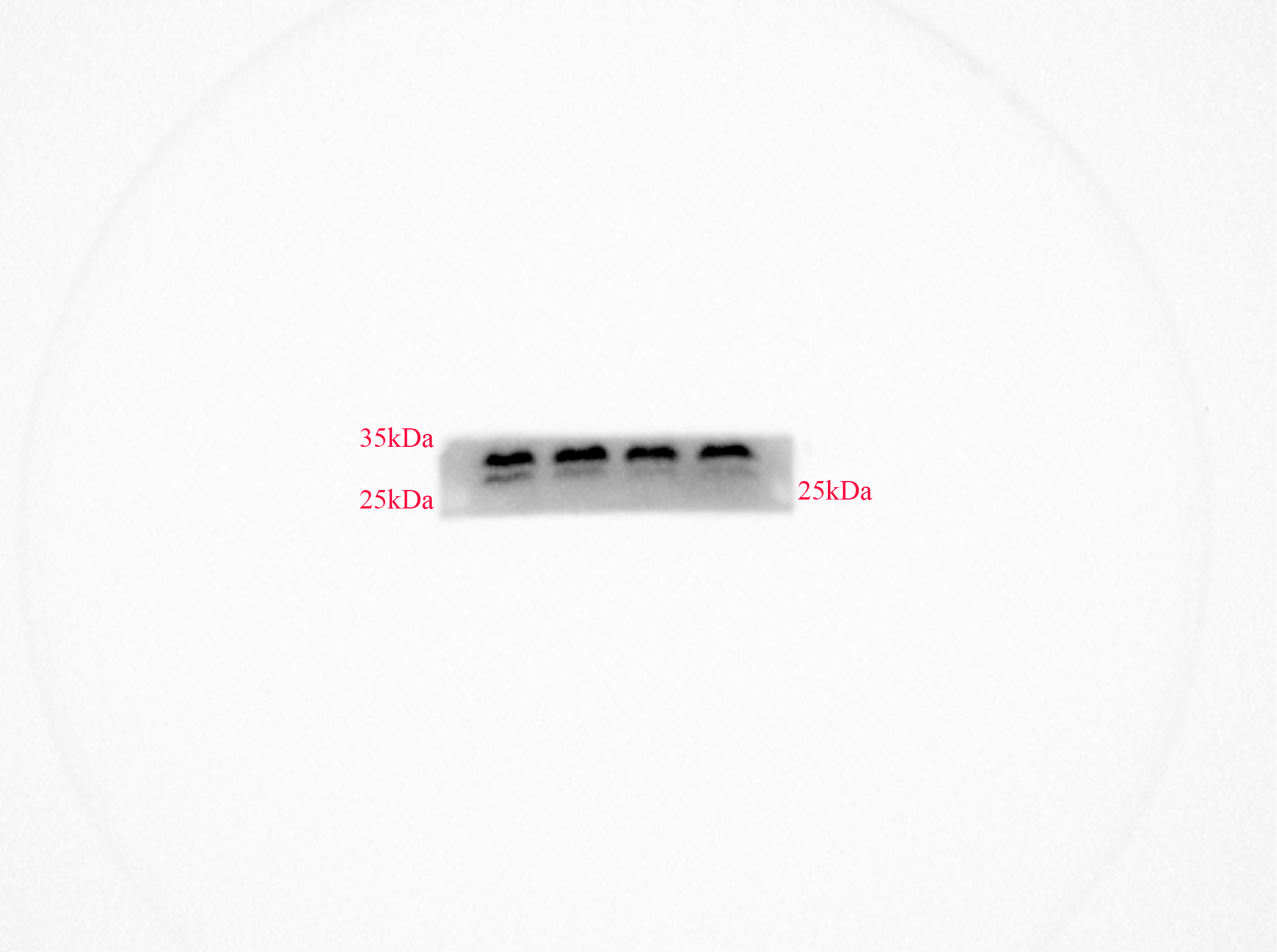

Supplement: Supplementary file 2 — Additional file 2. [file 12931_2025_3210_MOESM2_ESM.zip › WB RAW DATA - ╕▒▒╛/Figure3C/CASP3/lenovo 2022-10-05-Caspase-3-9-2.tif]

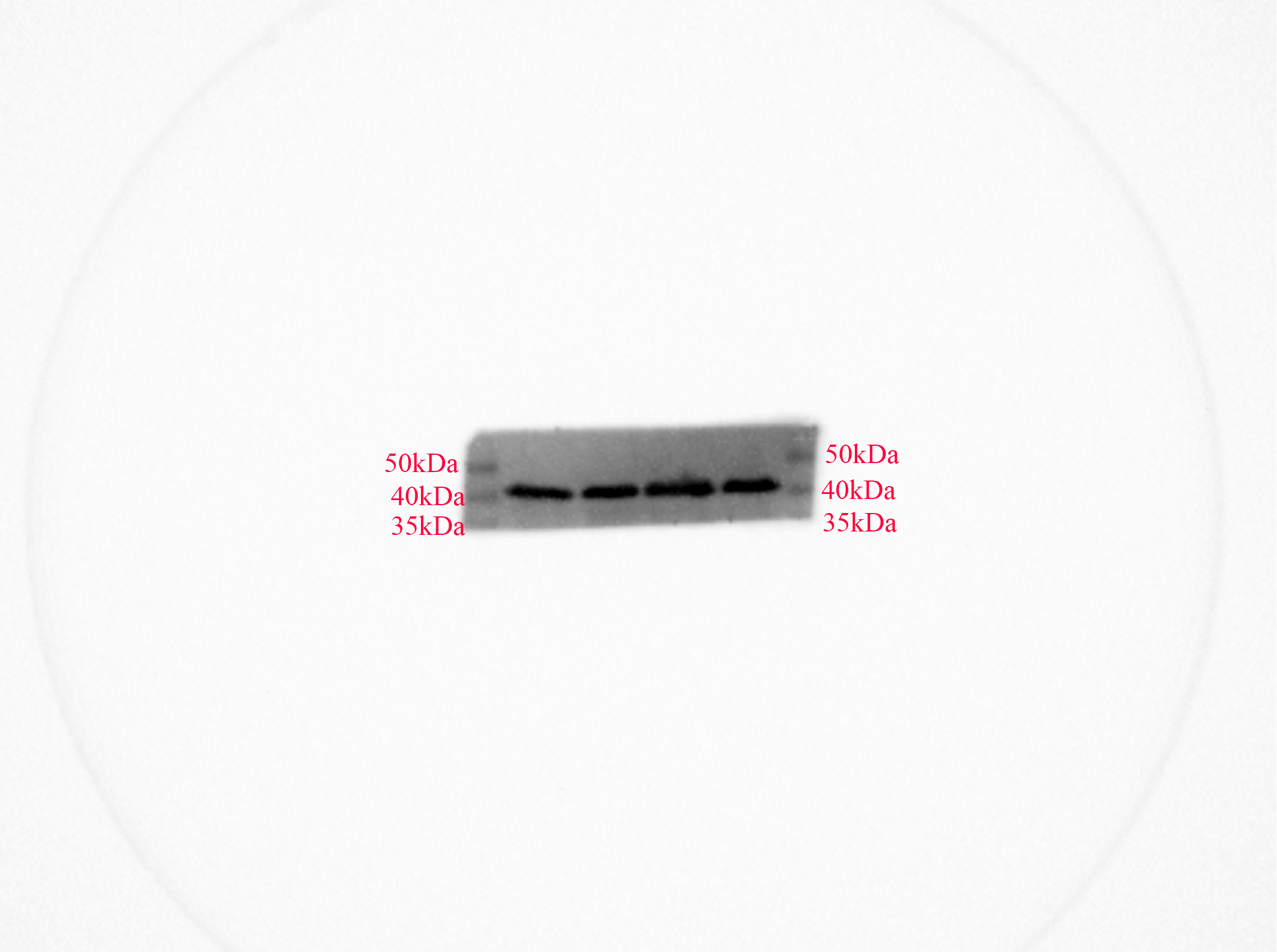

Supplement: Supplementary file 2 — Additional file 2. [file 12931_2025_3210_MOESM2_ESM.zip › WB RAW DATA - ╕▒▒╛/Figure3C/CASP9/lenovo 2022-10-20-beta-actin-9.tif]

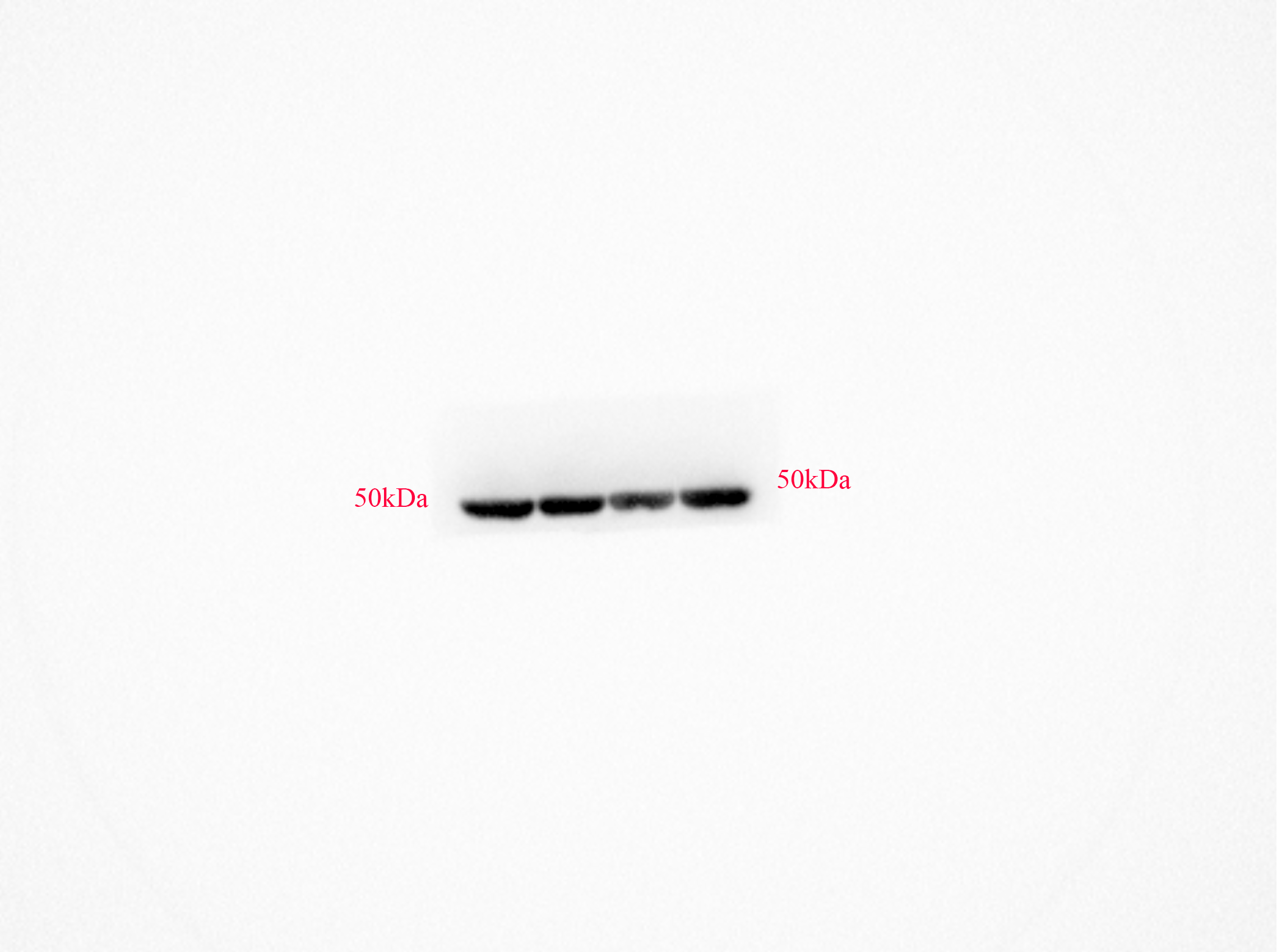

Supplement: Supplementary file 2 — Additional file 2. [file 12931_2025_3210_MOESM2_ESM.zip › WB RAW DATA - ╕▒▒╛/Figure3C/CASP9/lenovo 2022-10-22-Caspase9-2.tif]

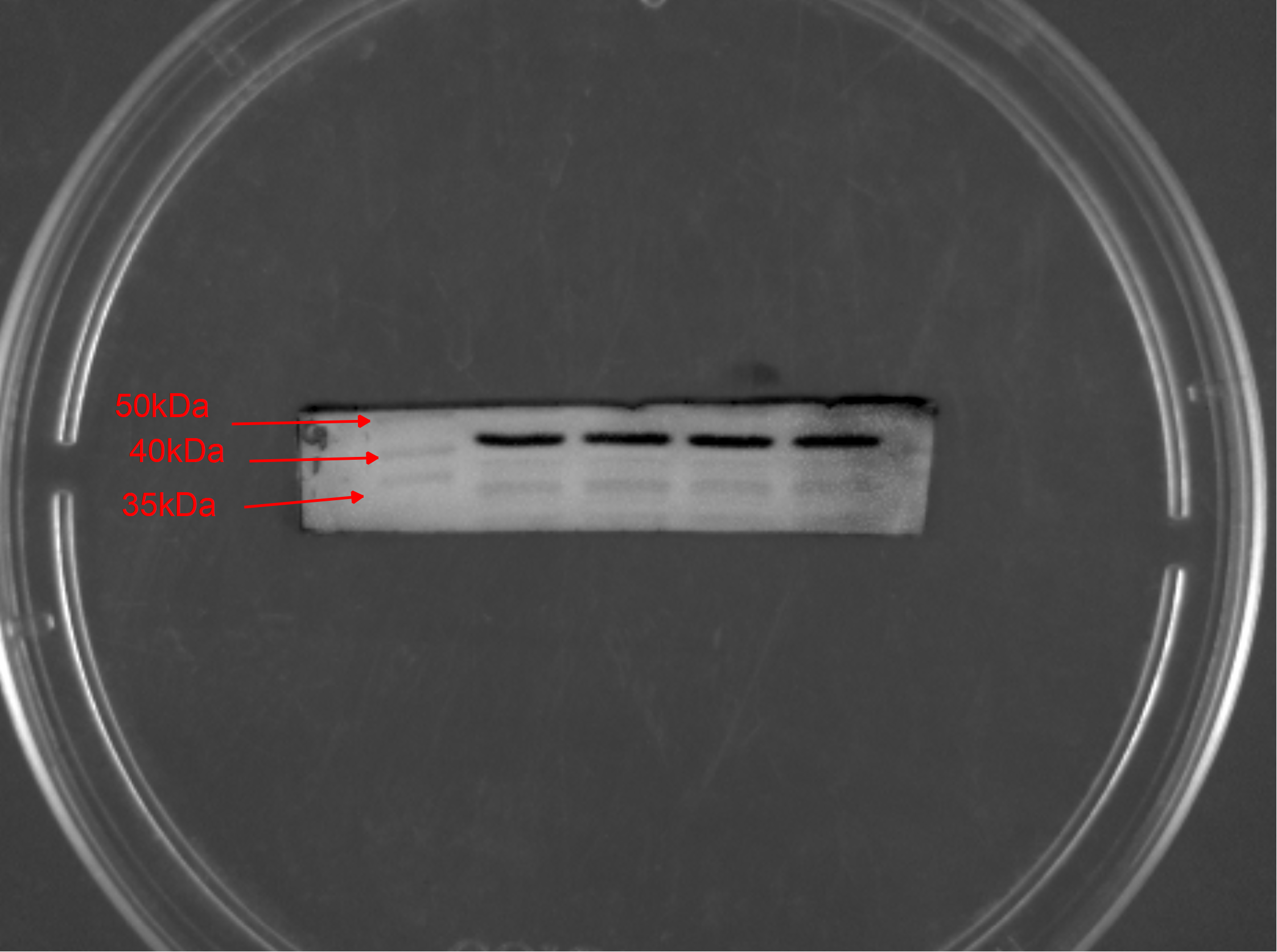

Supplement: Supplementary file 2 — Additional file 2. [file 12931_2025_3210_MOESM2_ESM.zip › WB RAW DATA - ╕▒▒╛/Figure3C/Cleaved-CASP3/lenovo 2022-09-05-beta-actin-1+lenovo 2022-09-05_12h56m07s.tif]

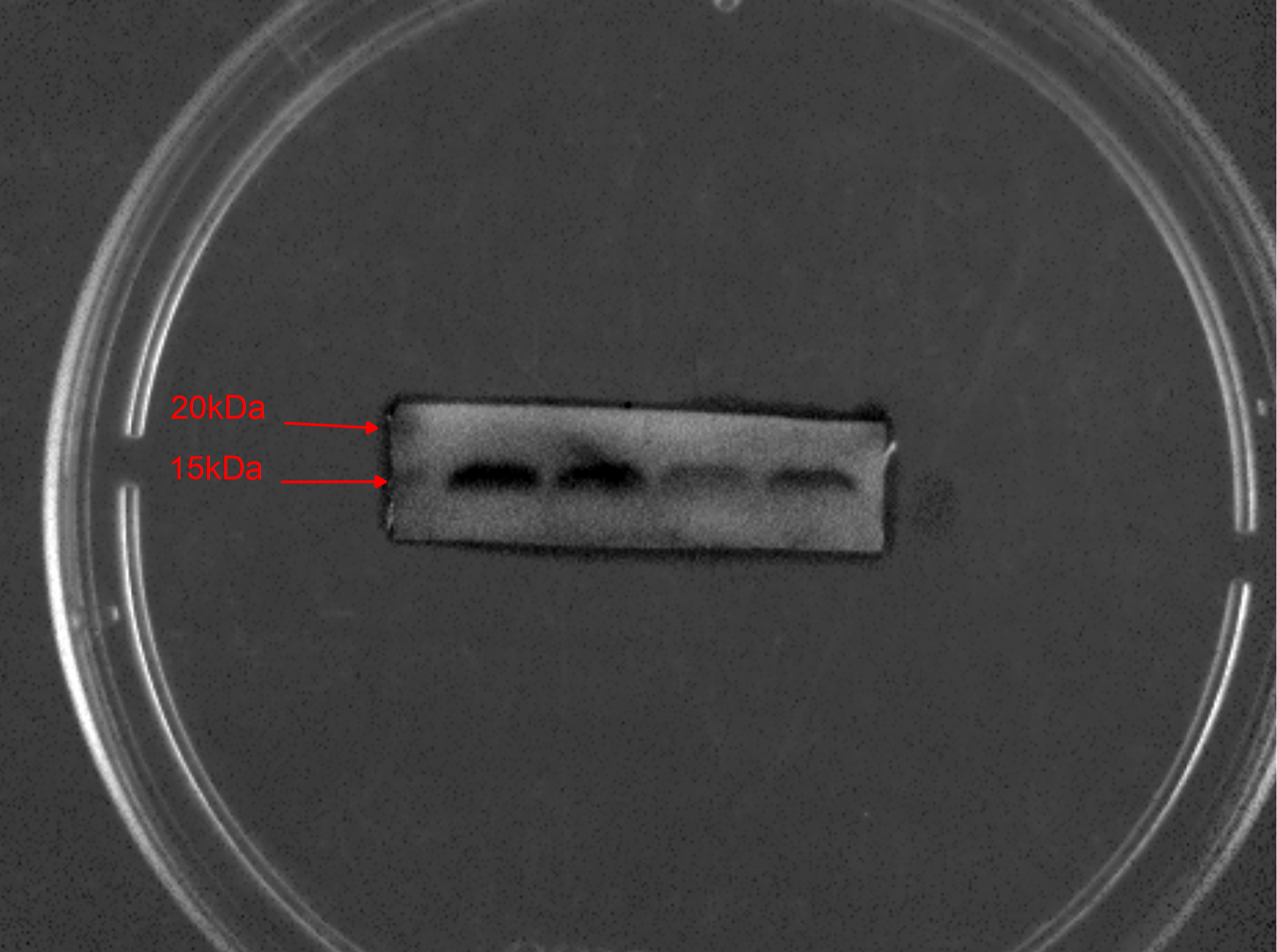

Supplement: Supplementary file 2 — Additional file 2. [file 12931_2025_3210_MOESM2_ESM.zip › WB RAW DATA - ╕▒▒╛/Figure3C/Cleaved-CASP3/lenovo 2022-09-06-Caspase3-3+lenovo 2022-09-06_12h43m43s.tif]

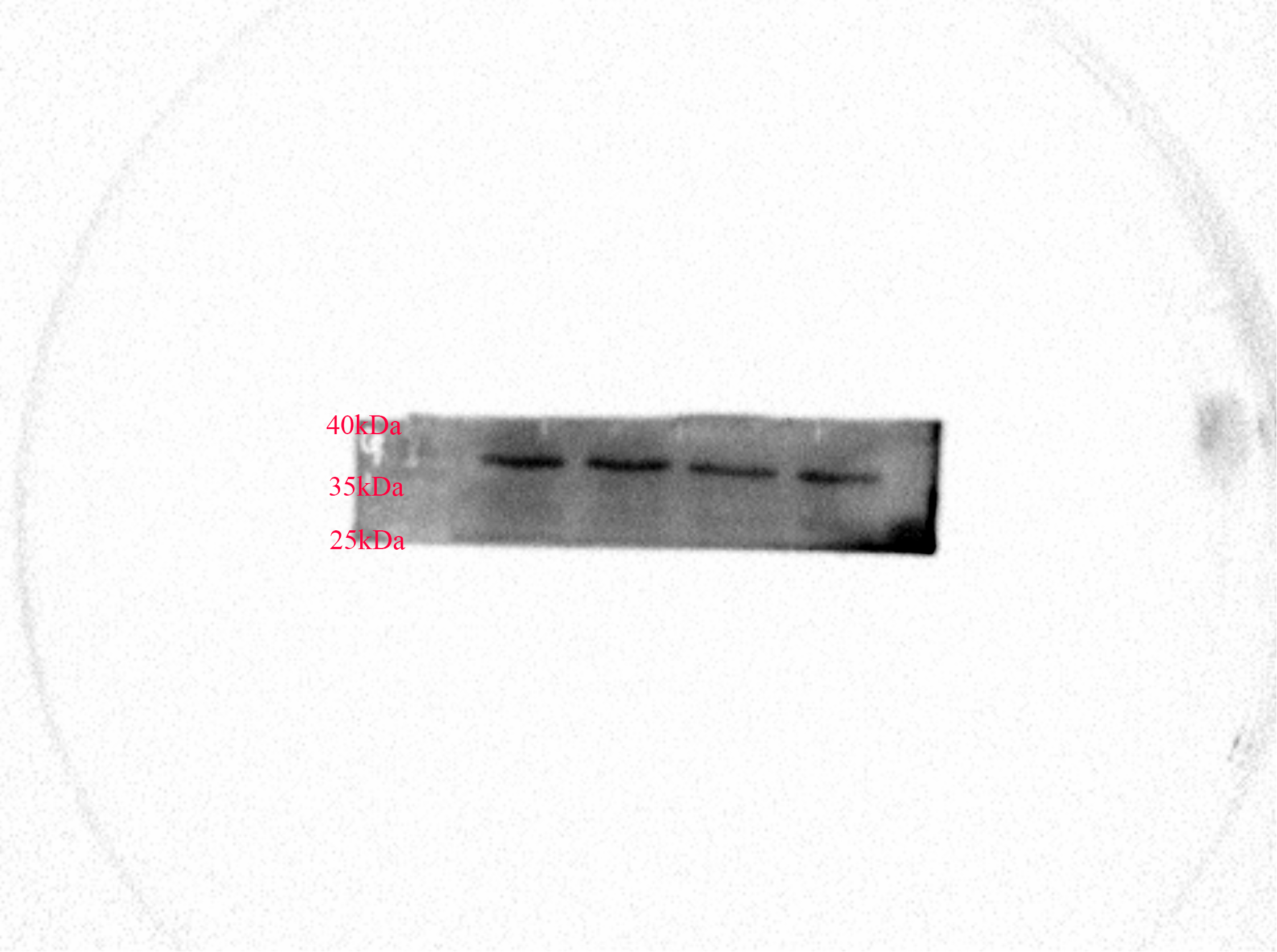

Supplement: Supplementary file 2 — Additional file 2. [file 12931_2025_3210_MOESM2_ESM.zip › WB RAW DATA - ╕▒▒╛/Figure3C/Cleaved-CASP9/lenovo 2022-09-06-Cleaved-Caspase9-1-2.tif]

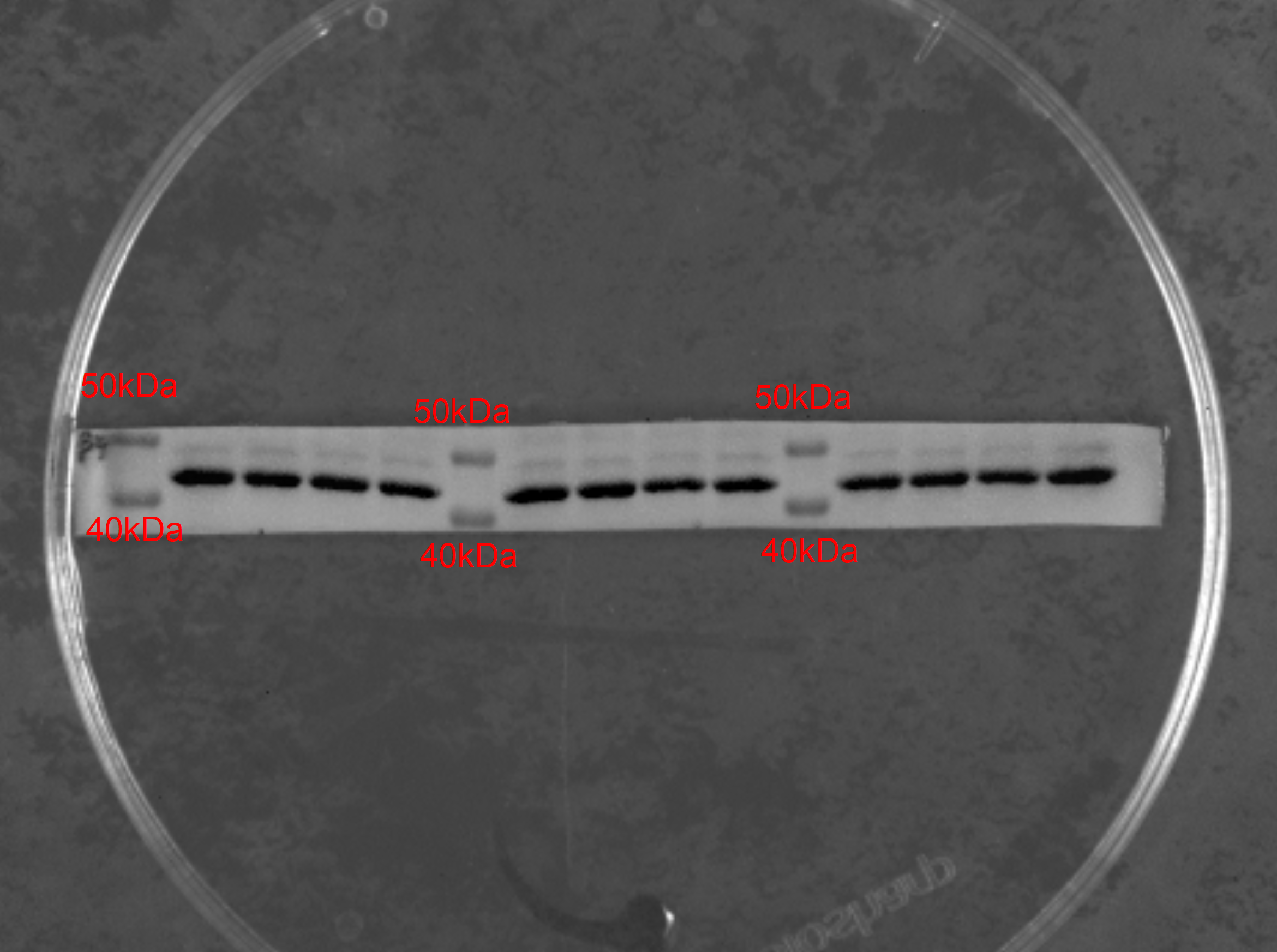

Supplement: Supplementary file 2 — Additional file 2. [file 12931_2025_3210_MOESM2_ESM.zip › WB RAW DATA - ╕▒▒╛/Figure4A/lenovo 2022-07-31_13h29m31s+lenovo 2022-07-31-Beta-actin-4.tif]

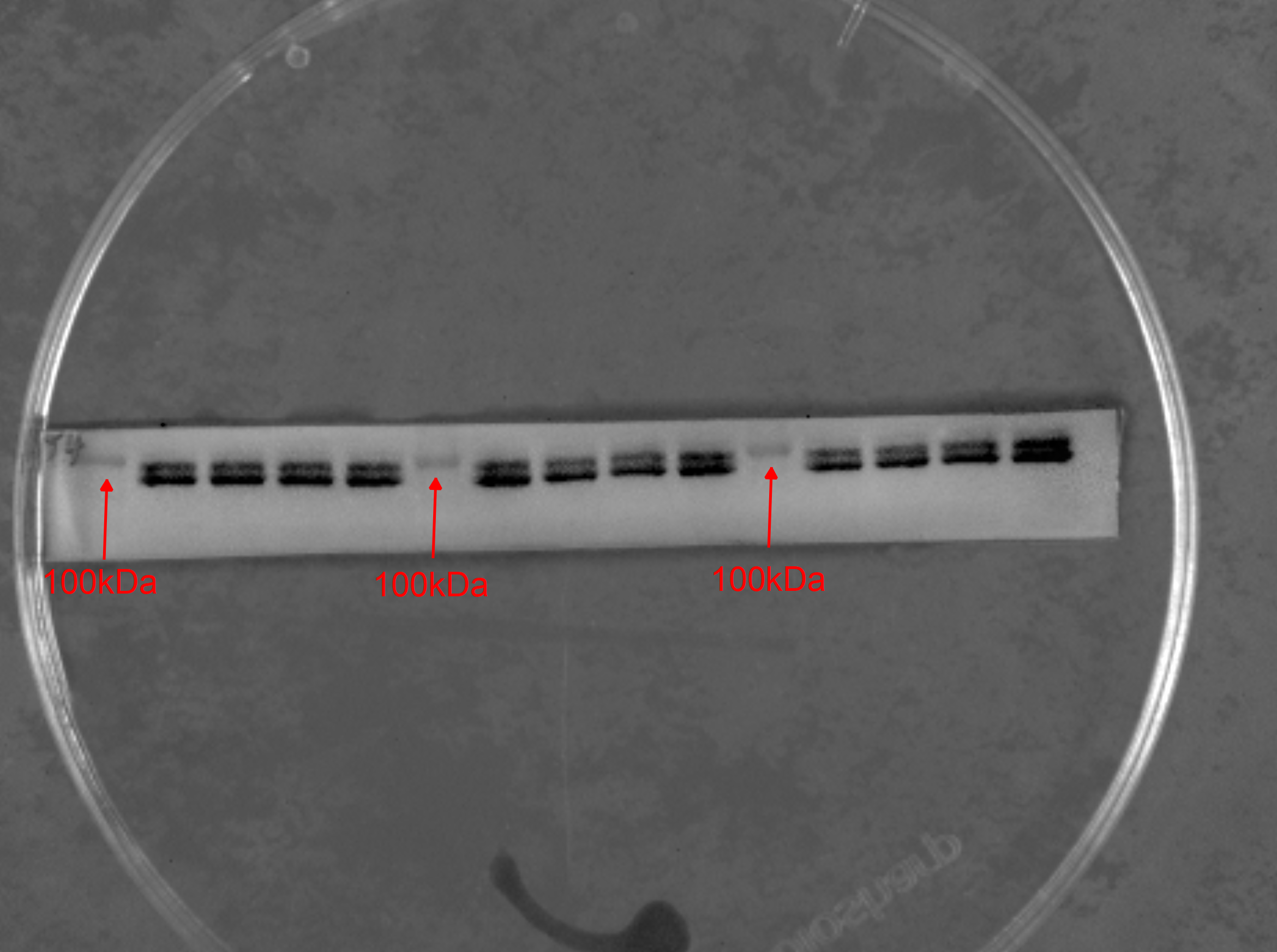

Supplement: Supplementary file 2 — Additional file 2. [file 12931_2025_3210_MOESM2_ESM.zip › WB RAW DATA - ╕▒▒╛/Figure4A/lenovo 2022-07-31_13h37m24s+lenovo 2022-07-31-TLR2-4.tif]

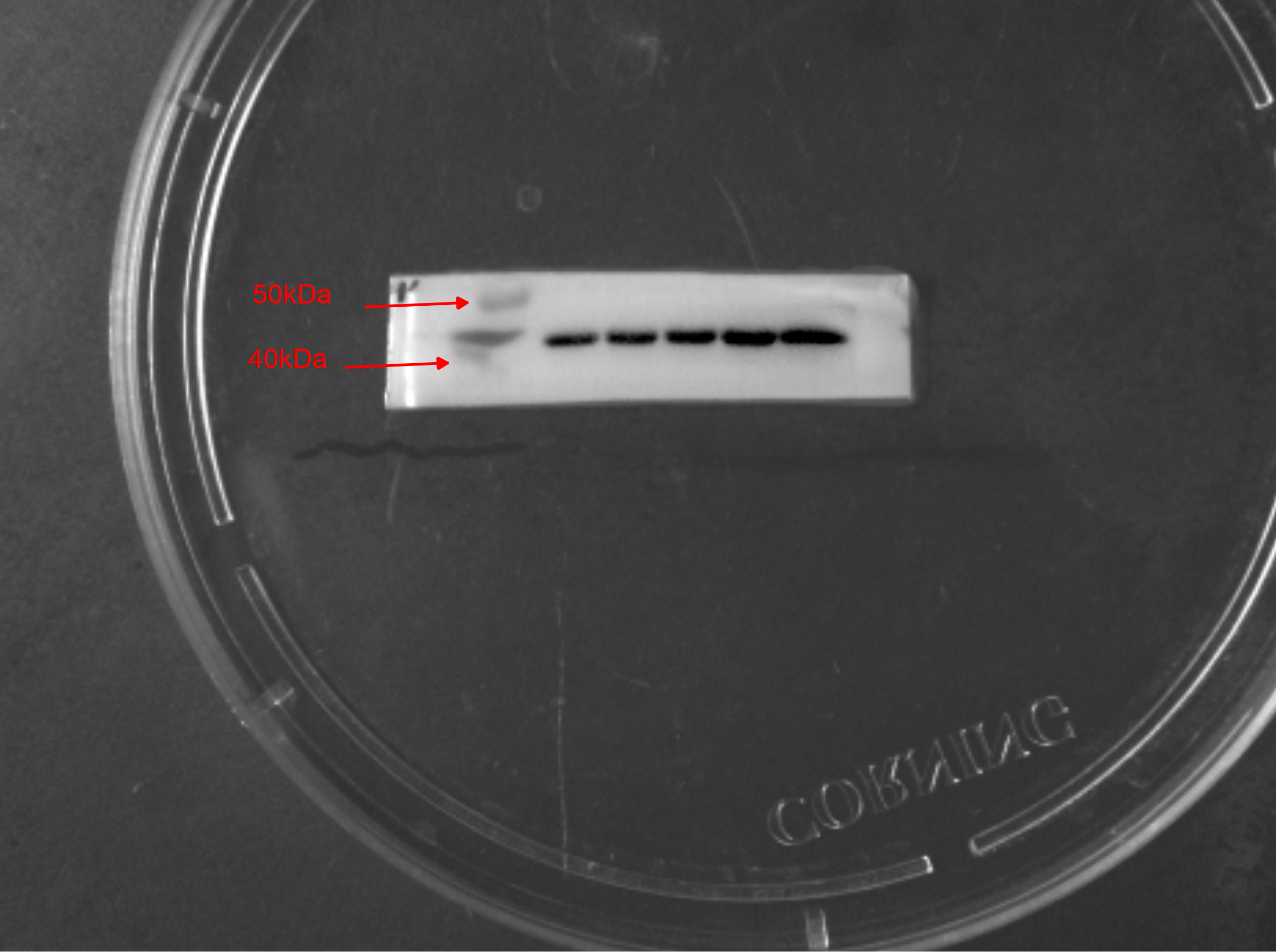

Supplement: Supplementary file 2 — Additional file 2. [file 12931_2025_3210_MOESM2_ESM.zip › WB RAW DATA - ╕▒▒╛/Figure4C/TLR2/lenovo 2021-08-13-beta-actin-1+lenovo 2021-08-13-beta-actin-1-marker.tif]

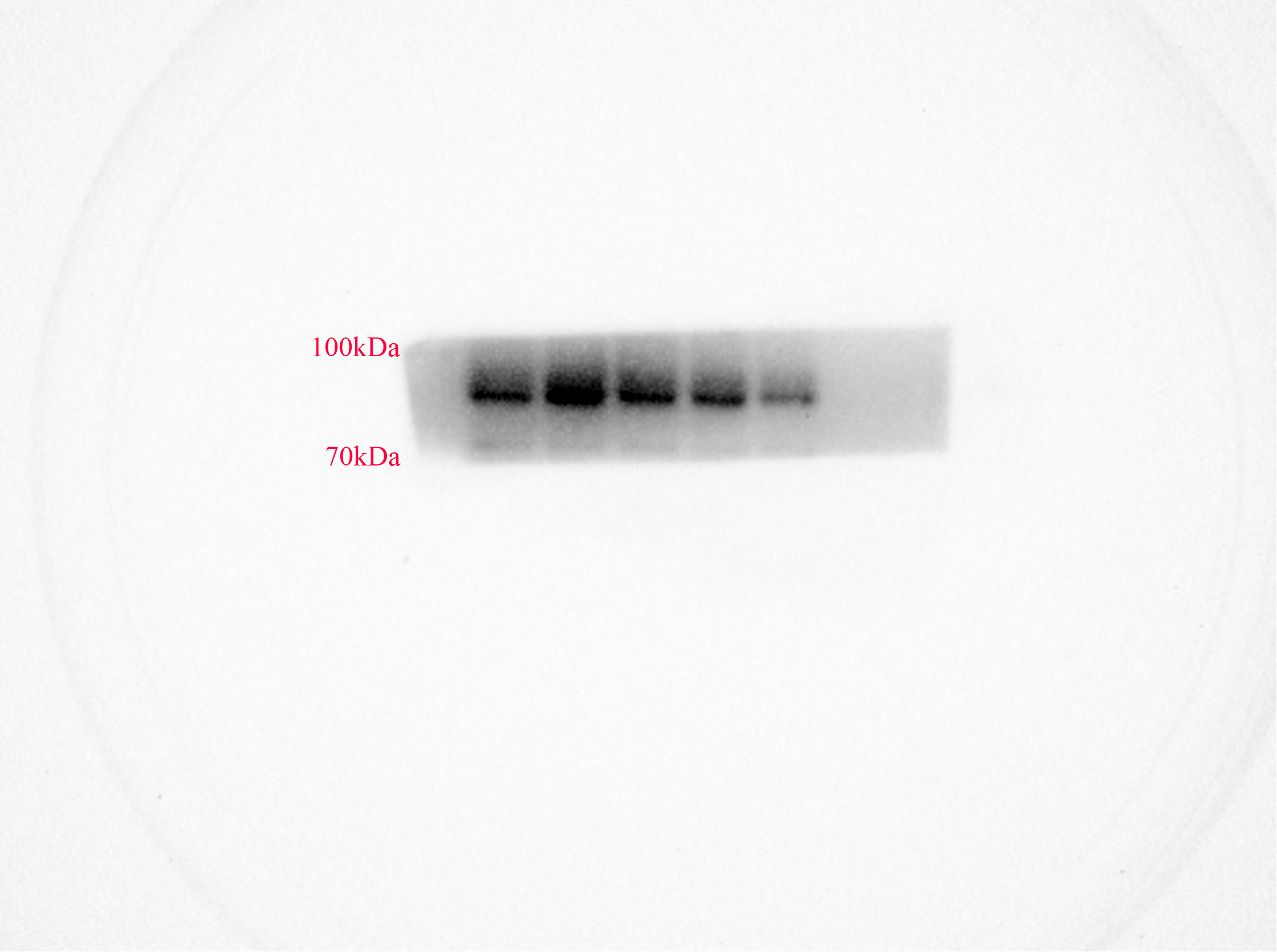

Supplement: Supplementary file 2 — Additional file 2. [file 12931_2025_3210_MOESM2_ESM.zip › WB RAW DATA - ╕▒▒╛/Figure4C/TLR2/lenovo 2021-08-13-TLR2-2-2.tif]

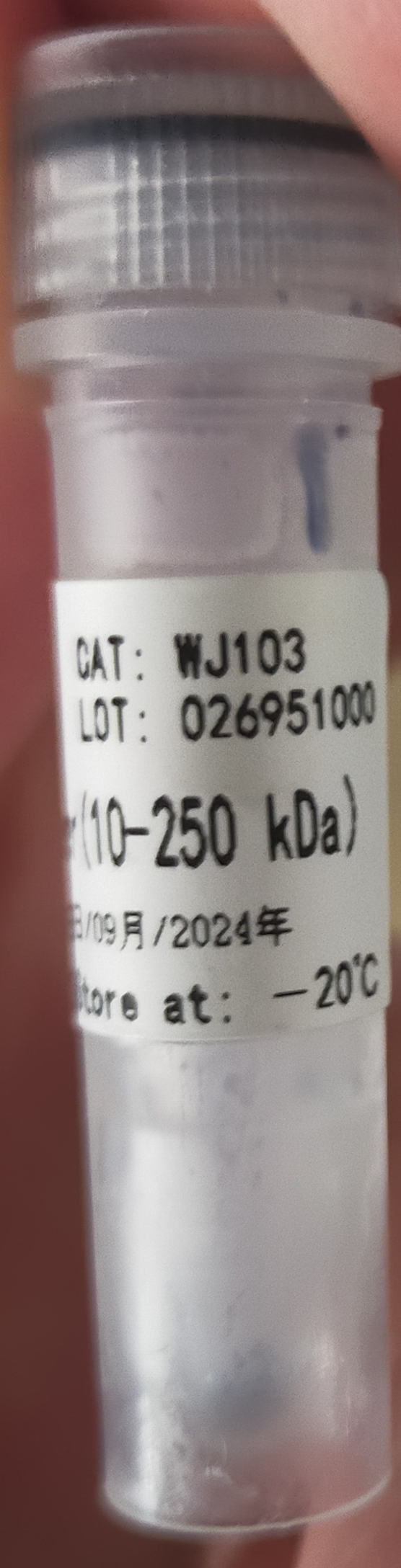

Supplement: Supplementary file 2 — Additional file 2. [file 12931_2025_3210_MOESM2_ESM.zip › WB RAW DATA - ╕▒▒╛/Ladder's Informatioans/Epizyme Biotech Ladder.tif]

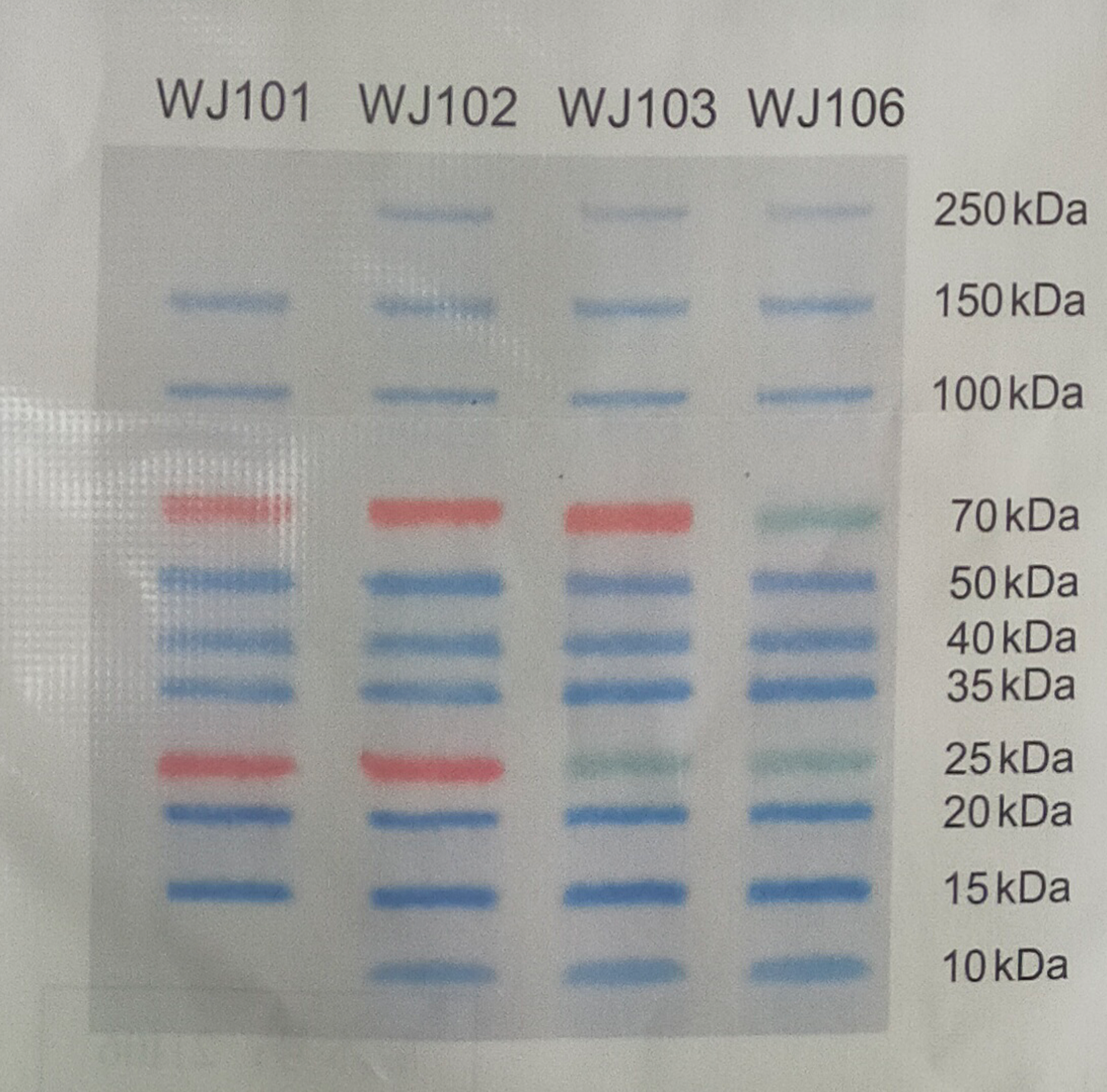

Supplement: Supplementary file 2 — Additional file 2. [file 12931_2025_3210_MOESM2_ESM.zip › WB RAW DATA - ╕▒▒╛/Ladder's Informatioans/Schema.tif]
